# Deletion of the sodium leak channel NALCN in dopamine neurons alters D2R‐mediated signalling and psychostimulant responsiveness

**DOI:** 10.1113/JP289809

**Published:** 2026-10-06

**Authors:** Angelina Rogliardo, Tinaig Le Borgne, Aylin Gulmez, Adeline Cathala, Claire Naon, Audrey Mignon, Maëlle Avrillon, Nejmeh Mashhour, Laia Castell, Claire Bernat, Jean‐Michel Revest, Arnaud Monteil, Philippe Faure, Federica Bertaso, Giuseppe Gangarossa, Fabio Marti, Emmanuel Valjent

**Affiliations:** ^1^ INM University Montpellier, Inserm Montpellier France; ^2^ Plasticité du Cerveau CNRS UMR8249 École supérieure de physique et de chimie industrielles de la Ville de Paris (ESPCI) Paris France; ^3^ Neurocentre Magendie, U1215 University Bordeaux, INSERM Bordeaux France; ^4^ Unité de Biologie Fonctionnelle et Adaptative Université Paris Cité, CNRS Paris France; ^5^ IGF University Montpellier, CNRS, Inserm Montpellier France; ^6^ Department of Physiology, Faculty of Medicine Siriraj Hospital Mahidol University Bangkok Thailand; ^7^ Institut Universitaire de France (IUF) Paris France; ^8^ Neuroscience Paris Seine CNRS UMR 8246 INSERM U1130, Institut de Biologie Paris Seine Sorbonne Université Paris France

**Keywords:** defensive behaviours, dopaminergic neurons, NALCN channel, pacemaker activity, psychostimulant responses

## Abstract

**Abstract:**

Midbrain dopamine (DA)‐neurons are slow pacemakers that continuously fire action potentials, maintaining basal extracellular DA levels in target regions. The sodium leak channel NALCN has been identified as a key contributor in the pacemaker control of these neurons. However, the consequences of a disrupted DA pacemaker activity remain largely unexplored. By generating mice lacking NALCN selectively in midbrain DA‐neurons, we investigated the impact of this deletion on the activity of ventral tegmental area DA‐neurons, D2R‐mediated signalling, reactivity to cues predicting threat and psychostimulant responsiveness in both males and females. Selective deletion of *NALCN* in midbrain DA‐neurons dampens their tonic activity, without reducing the basal extracellular DA levels in the nucleus accumbens. Mice with a loss of NALCN in DA‐neurons exhibit no impairments of basal motor responses, unaltered reactivity to cues predicting threat and risk assessment, while the neurochemical and behavioural responsiveness to psychostimulant drugs are enhanced and presynaptic D2R‐mediated signalling at DA terminals is altered. Together, our results emphasize the important role of NALCN conductance in controlling DA pacemaker activity, D2R‐mediated signalling and in modulating motor responses to psychostimulants.

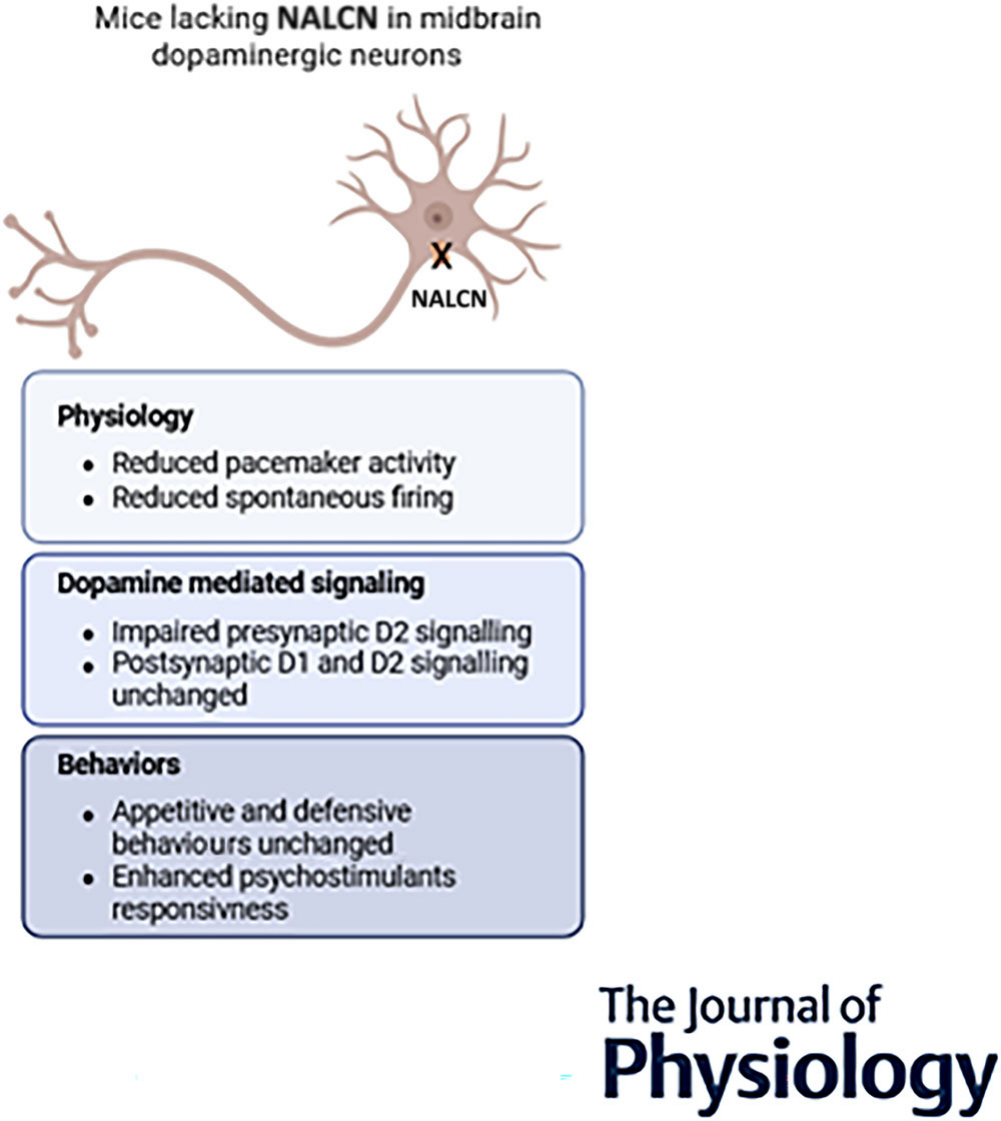

**Key points:**

- The sodium leak channel NALCN is an important contributor to the spontaneous pacemaker activity in dopamine neurons, but the consequences of its loss in these neurons remain largely unknown.
- We investigated how selective deletion of NALCN in dopamine neurons affects neuronal activity, dopamine receptor signalling and behavioural responses in male and female mice.
- Loss of NALCN markedly reduced the spontaneous activity of dopamine neurons, yet basal motor function, reward‐related responses and reactivity to threat‐predicting cues remained largely preserved.
- In contrast, neurochemical and behavioural responsiveness to psychostimulant drugs are enhanced, probably through an altered presynaptic D2R‐mediated signalling at DA terminals.
- NALCN emerges as a key regulator of dopamine neuron activity, with disrupted pacemaker firing selectively enhancing psychostimulant sensitivity.

## Graphical abstract

**Abstract figure legend** The sodium leak channel NALCN is an important contributor to the spontaneous pacemaker activity of midbrain dopamine neurons and is required for proper presynaptic D2 autoreceptor signalling at dopaminergic terminals. Conditional deletion of NALCN in dopamine neurons enhances psychomotor sensitization to amphetamine and cocaine, without affecting appetitive and defensive behaviours. These findings reveal NALCN as a novel regulator of dopaminergic transmission that gates the responsiveness of the mesolimbic system to psychostimulant drugs.

## Introduction

Dopamine (DA)‐neurons play a central role in coding reward prediction errors and motivational salience of stimuli regardless of their positive or negative values (Bromberg‐Martin et al., 2010; Schultz, 1998). These functions are sustained by the ability of DA‐neurons to switch their modality of discharge from slow irregular tonic activity, thought to set the basal levels of DA at synaptic targets, to rapid burst firing, which conveys reward and/or cues predicting reward of salient events (Bromberg‐Martin et al., 2010; Schultz, 2016). In contrast to phasic activation, which is synaptically driven (Roeper, 2013), tonic firing mainly relies on the autonomous pacemaker activity generated by the coordinated action of distinct classes of ion channels including voltage‐dependent calcium and sodium channels (Khaliq & Bean, 2010; Puopolo et al., 2007), A‐type potassium channels (Juarez et al., 2023; Khaliq & Bean, 2008; Liss et al., 2001) and calcium‐activated potassium (SK) channels (Wolfart et al., 2001).

Among the different ion conductance that tune the intrinsic rhythmicity of DA‐neurons, those mediating the slow subthreshold depolarization during interspike intervals have been proposed as central to sustaining their robust pacemaker activity (Khaliq & Bean, 2010). Recent pharmacological and genetic studies have demonstrated that this feature is supported by the synergistic activation of the transient receptor potential cation channel subfamily C member 3 (TRPC3) and the non‐selective sodium leak channel NALCN (Cobb‐Lewis et al., 2023; Philippart & Khaliq, 2018; Um et al., 2021). Despite this evidence, the influence of altered pacemaker activity resulting from the invalidation of the non‐selective sodium leak channel NALCN in midbrain DA‐neurons on DA‐related behaviours remains unexplored.

Here, we reveal the importance of NALCN conductance in regulating the tonic activity of DA‐neurons of the ventral tegmental area (VTA), one of the major sources of DA in the brain. Despite this, mice lacking NALCN in DA‐neurons display normal basal extracellular DA levels in the nucleus accumbens (Acb). Moreover, basal locomotor activity, risk assessment or defensive behaviours engaged in response to threat‐associated stimuli were not impaired. In contrast, we report that NALCN in midbrain DA‐neurons is a fundamental regulator of the psychomotor responsiveness to psychostimulant drugs. These findings identify NALCN as a key modulator of both intrinsic DA‐neuron excitability and sensitivity to psychostimulants.

## Materials and methods

### Ethical approval

All animal experiments were conducted in accordance with the European Directive 2010/63/EU and the French Ministry of Agriculture and Forestry regulations governing the care and use of laboratory animals (authorization number/licence B34‐172‐41). Experimental procedures were approved by the relevant local ethical committees and by the French Ministry of Higher Education and Research (APAFIS#38912, APAFIS#22635, APAFIS#32638 and APAFIS#24037). The authors understand the ethical principles under which *The Journal of Physiology* operates and confirm that the work complies with the journal's animal ethics checklist.

### Animals

Male and female C57BL/6J mice (3 months old; Charles River Laboratories, France) were used for RNAscope experiments.

The deletion of *Nalcn* from DA‐neurons was carried out by crossing Slc6a3‐Cre mice with *Nalcn^loxP/loxP^* mice (Flourakis et al., 2015) (kindly provided by Prof. D. Ren). The generated male and female *Nalcn^Slc6a3^* mice were compared to littermate controls (controls: Cre‐negative Nalcn^f/f^ mice). Mice were housed in groups of two to five per cage, maintained on a 12:12 h light/dark cycle in stable conditions of temperature (22°C) and humidity (60%) with food and water provided *ad libitum*.

Animals were included only when genotype was confirmed and all experimental procedures were completed successfully. Exclusion criteria were predefined and included misplaced microdialysis probes, failed viral expression, inadequate histological verification or poor‐quality electrophysiological recordings.

### Drugs

Cocaine hydrochloride (15 mg/kg) and methylphenidate hydrochloride (7.5 and 10 mg/kg) were purchased from Sigma‐Aldrich (St. Louis, MO, USA). Eticlopride hydrochloride (0.5 and 1 mg/kg), d‐amphetamine sulphate (1.5 mg/kg), GBR12783 dihydrochloride (15 and 30 mg/kg), SKF81297 hydrobromide (5 mg/kg) and quinpirole hydrochloride (0.05, 0.5 and 1 mg/kg) were purchased from Tocris (Bristol, UK). Except GBR12783, which was dissolved in H_2_O, all drugs were dissolved in a saline solution (0.9% NaCl) and administered intraperitoneally (i.p.) or intravenously (i.v.) in a body volume of 10 ml/kg.

### Histology

#### Immunofluorescence

Tissue preparation and immunofluorescence analyses were performed as previously described (Cutando et al., 2021). Mice were anaesthetized with Euthasol® (360 mg/kg, i.p., TVM lab, France) and transcardially perfused with 4% (w/v) paraformaldehyde in 0.1 m sodium phosphate buffer (pH 7.5). Brains were post‐fixed overnight in the same solution and stored at 4°C. Free‐floating sections (40 µm) were washed in PBS, and incubated for 15 min in 0.2% Triton X‐100 in PBS. Slices were then incubated in PBS for 72 h at 4°C with the primary antibodies (Table 1). Slices were rinsed three times in PBS and incubated for 45 min with secondary fluorescent antibodies (Table 1). Sections were rinsed twice in PBS before mounting in 1,4‐diazabicyclo‐[2.2.2]‐octane (DABCO, Sigma‐Aldrich). Images were captured using sequential laser scanning confocal microscopy (Leica SP8) at the Montpellier RIO imaging facility and pseudocoloured in orange or yellow. Images used for quantification of pS10‐H3 and FOS‐positive cells were all single confocal sections.

**Table 1:** Detailed antibody information

Antigen	Species	Dilution	Supplier/catalogue no./references
**Primary antibodies**			
DAT	Guinea pig	1:1000 (IF)	Frontier Institute (#Af660)
DAT	Rat	1:1000 (WB)	Millipore (#mab369)
TH	Rabbit	1:1000 (IF); 1:2000 (IF)	Millipore (#AB152)
TH	Mouse	1:200 (IF)	Sigma (T1299)
VMAT2	Rabbit	1:500 (WB)	Synaptic System (#138302)
pS31‐TH	Rabbit	1:1000 (WB)	Cell Signaling (#3370S)
pS40‐TH	Rabbit	1:500 (WB)	Cell Signaling (#2791S)
D2R	Guinea pig	1:1000 (IF)	Frontier Institute (#D2R‐GP‐Af500)
pS10‐H3	Rabbit	1:1000 (WB); 1:500 (IF)	Millipore (#06‐570)
FOS	Rat	1:5000 (IF)	Synaptic System (#226017)
D1R	Rat	1:1000 (IF)	Sigma‐Aldrich (#D2944)
D1R	Mouse	1:1000 (WB)	Homemade (R.R. Luedtke)
GluA1	Rabbit	1:500 (WB)	Millipore (#AB1504)
DARPP‐32	Rabbit	1:1000 (IF); 1:1000 (WB)	Cell Signaling Technology (#2306)
Gαolf	Rabbit	1:1000 (IF); 1:1000 (WB)	Homemade (Girault/D. Hervé)
D2R	Rabbit	1:1000 (WB)	Millipore (#AB5084P)
β‐actin	Mouse	1:2000 (WB)	Sigma Aldrich (#A1978)
**Secondary antibodies**			
HRP	Rabbit	1:10,000 (WB)	Cell Signaling (#7074S)
HRP	Mouse	1:10,000 (WB)	Cell Signaling (#7076S)
A647	Rat	1:1000 (IF); 1:500(IF)	Invitrogen (#A21247)
A488	Rabbit	1:1000 (IF)	Lift Tech (#A11034)
CY3	Rabbit	1:500 (IF)	Invitrogen (#A10520)
A488	Goat	1:500 (IF)	Invitrogen (#A11039)
A647	Guinea Pig	1:500 (IF)	Invitrogen (#A21450)

#### Immunofluorescence after electrophysiological slice recording

After patch‐clamp recordings (see below), the neurochemical phenotype of biocytin‐filled VTA DA‐neurons was confirmed by immunoreactivity to tyrosine hydroxylase (TH). To this end, recorded slices were fixed in 4% PFA (paraformaldehyde) overnight. Free‐floating VTA brain sections were incubated for 3 h at 4°C in a blocking solution of phosphate‐buffered saline (PBS) containing 3% bovine serum albumin (BSA, Sigma; A4503) (v/v) and 0.2% Triton X‐ (v/v), and then incubated for 72 h at 4°C with a mouse anti‐TH antibody (Table 100 (v/v), and then incubated for 72 h at 4°C with a mouse anti‐TH antibody (Table 1) in PBS containing 1.5% BSA and 0.2% Triton X‐100. After three nights, sections were rinsed with PBS, and then incubated for 6 h at room temperature with secondary antibody Cy3‐conjugated anti‐mouse and AMCA‐conjugated streptavidin (Jackson ImmunoResearch, UK) both at 1:200 dilution in a solution of 1.5% BSA in PBS. After three rinses in PBS, slices were wet‐mounted using Prolong Gold Antifade Reagent (Invitrogen, France; P36930). Microscopy was carried out with a fluorescence microscope, and images were captured using a camera and analysed with ImageJ.

#### *In situ* hybridization

Brains were rapidly extracted, placed on dry ice for 5 min and stored at −80°C. Tissue was included in an embedding medium to ensure optimal cutting temperature and then sectioned on a cryostat at 16 µm. Striatal and VTA coronal sections were collected onto Superfrost Plus slides (Fisher Scientific). *In situ* hybridization was carried out using the RNAscope Fluorescent Multiplex labelling kit (ACDBio, Biotechne, France; catalogue no. 323110) and the protocol previously described (Castell et al., 2024). Probes used include Mm‐Drd2‐C3 (ACDBio catalogue no. 406501‐C3), Mm‐Slc32a1‐C2 (ACDBio catalogue no. 319191‐C2), MM‐Th‐C2 (ACDBio catalogue no. 317621‐C2), Mm‐Slc6a3‐C3 (ACDBio catalogue no. 315441‐C3) and Mm‐Nalcn‐C1 (ACDBio catalogue no. 415165). Hybridized slices were counterstained with DAPI and mounted with ProLong Diamond Antifade mounting medium (Thermo Fisher Scientific, France; catalogue no. P36961). Imaging was carried out at the Montpellier RIO imaging facility. Triple‐ and double‐labelled images from the striatum and the VTA were single confocal sections captured using sequential laser scanning confocal microscopy (Leica SP8).

### Western blotting

Western blotting was performed as previously described (Puighermanal et al., 2020). Briefly, the brain was rapidly extracted and the striatum dissected on an ice‐cold surface. Tissues were sonicated in 300 µL of lysis buffer (1% SDS‐PBS containing 0.2% phosphatase inhibitors and 1% protease inhibitors) and boiled for 10 min. Aliquots (2.5 µL) were used for protein quantification using a BCA kit (BC Assay Protein Quantitation Kit, Interchim Uptima, Montluçon, France). Equal amounts of proteins (15 µg), supplemented with Laemmli buffer, were loaded onto 10% polyacrylamide gels. Proteins were separated by SDS‐PAGE and transferred (Trans‐Blot® TurboTM, Bio‐Rad, France) to 0.2 µm PVDF membranes (Bio‐Rad, no. 1704156). Membranes were immunoblotted with primary antibodies described in Table 1. Detection was based on HRP‐coupled secondary antibody binding using ECL. The secondary antibodies are listed in Table 1. Membranes were imaged using the Amersham Imager 680. Quantifications were performed using ImageJ software.

### Microdialysis and dopamine assay

Surgery and perfusion procedures were performed as previously described (Cathala et al., 2022) with minor modifications. For all experiments, the microdialysis probe (CMA/11, cuprophan, 240 µm outer diameter, Carnegie Medicin, Phymep, France) was 1 mm in length for the Acb. Mice were first injected with meloxicam (1 mg/kg, s.c.) 30 mins before being anaesthetized with 3% isoflurane, and placed in a stereotaxic frame. A local anaesthetic (lidocaine) was applied to the incision site, and mice were then injected with saline at the end of the surgery to maintain their hydration throughout the experiment. The microdialysis probe was implanted according to the atlas of Franklin and Paxinos (2008) into the Acb (coordinates, in mm, relative to the Bregma point, AP = 1.35, L = −0.5, V = −5.2). After the surgery, the percentage of isoflurane was adjusted to 1.5% until the end of the experiment. Probes were perfused at a constant flow rate (1 µL/min), by means of a microperfusion pump (CMA 111, Carnegie Medicin, Phymep), with artificial cerebrospinal fluid (ACSF) containing (in mM): 147 NaCl, 2.7 KCl, 1.2 CaCl_2_ and 1 MgCl_2_ in phosphate buffer, pH 7.4. Dialysates were collected in a refrigerated fraction collector (MAB 85 Microbiotech, Phymep) 120 min after the beginning of the perfusion (stabilization period) and every 15 min. DA outflow was monitored during 60 min. After collection, dialysate samples were immediately analysed with a high‐performance liquid chromatography apparatus (Alexys UHPLC/ECD Neurotransmitter Analyzer, Antec, The Netherlands), equipped with an autosampler (AS 110 UHPLC cool 6‐PV, Antec), as previously described (Cathala et al., 2022). The mobile phase [containing (in mM) 100 phosphoric acid, 100 citric acid, 0.1 EDTA.2H_2_O, 4.6 octanesulfonic acid.NaCl plus 5% acetonitrile, adjusted to pH 6.0 with NaOH solution (50%)] was delivered at a flow rate of 0.070 ml/min with an LC 110S pump (Antec, The Netherlands) through an Acquity UPLC BEH column (C18; 1 × 100 mm, particle size 1.7 µm; Waters, Saint‐Quentin en Yvelynes, France). Detection of DA was carried out with an electrochemical detector (DECADE II, Antec) with a VT‐03 glassy carbon electrode (Antec) set at +460 mV *versus* Ag/AgCl. Output signals were recorded on a computer (Clarity, Antec). Under these conditions, the retention time for DA was 3–3.5 min and the sensitivity was 50 pM with a signal/noise ratio of 3:1. At the end of each experiment, the animal was deeply anaesthetized with a pentobarbital overdose (Exagon, 200 mg/kg, Centravet, France), and its brain was removed and fixed in NaCl (0.9%)/paraformaldehyde solution (10%). Probe location into the Acb was determined histologically on serial coronal sections (60 µm), and only data obtained from animals with correctly implanted probes were included in the results. DA content in each sample was expressed as the percentage of the average baseline level calculated from the three fractions preceding drug administration (i.e, cocaine, d‐amphetamine, eticlopride or control vehicle). Data correspond to the mean ± SEM of the percentage obtained in each experimental group.

### Electrophysiology

#### *In vivo* electrophysiology on anaesthetized animals: juxtacellular recordings

Adult male mice (8–12 weeks) were deeply anaesthetized with chloral hydrate (8%) (400 mg/kg, i.p.), supplemented as required to maintain optimal anaesthesia throughout the experiment. Mice were then placed in a stereotaxic frame (David Kopf), the scalp was opened and a cranial window was drilled in the skull above the location of the VTA (coordinates: 3.1 ± 3 mm posterior to bregma, 0.4–0.5 mm lateral to the midline, 3.9–5 mm ventral from the brain). Recording electrodes were pulled from borosilicate glass capillaries (Harvard Apparatus, Les Ulis, France; outer and inner diameters of 1.50 and 1.17 mm, respectively) with a Narishige electrode puller (Narishige, London, UK). The tips were broken under microscope control and filled with 0.5% sodium acetate. Electrodes had tip diameters of 1–2 µm and impedances of 6–9 MΩ. A reference electrode was placed in the subcutaneous tissue. The recording electrodes were lowered vertically through the hole with a micro drive. Electrical signals were amplified by a high‐impedance amplifier (Axon Instruments, Molecular Devices, UK) and monitored through an audio monitor (A.M. Systems Inc.). The unit activity was digitized at 12.5 kHz and recorded using Spike2 software (Cambridge Electronic Design, Cambridge, UK). Individual electrode tracks were separated from one another by at least 0.1 mm in the medio‐lateral axis. The electrophysiological characteristics of DA‐neurons were analysed in the active cells encountered when passing the microelectrode in a stereotaxically defined block of brain tissue corresponding to the coordinates of the VTA. Extracellular identification of putative DA‐neurons was based on their location as well as on the set of unique electrophysiological properties that distinguish DA from non‐DA neurons *in vivo*: (i) a typical triphasic action potential (AP) with a marked negative deflection; (ii) a long duration (>2.0 ms); (iii) an action potential width from start to negative trough > 1.1 ms; and (iv) a slow firing rate (<10 and >1 Hz). Electrophysiological recordings were analysed using the R software. DA cell firing was analysed with respect to the average firing rate and the percentage of spikes within bursts (% SWB, number of spikes within a burst divided by the total number of spikes). Bursts are classically identified as discrete events consisting of a sequence of spikes with (i) a burst onset defined by two consecutive spikes within an interval <80 ms and (ii) the end of a burst defined by an inter‐spike interval >160 ms. Experimenters were blinded to the mice's genotype during recording. To study the response of DA‐neurons to nociceptive stimuli, three tail pinches (duration 5 s, interval 1 min) were applied manually. To record the response of silent, putative DA‐neurons to nociceptive stimuli, microelectrodes were slowly lowered (4 µm steps) into the VTA until an increase in resistance indicated proximity to a cell. Next, three manual tail pinches (5 s duration, 1 min intervals) were applied to induce AP. For each neuron, the average number of APs was calculated across the three tail‐pinch trials. Neurons exhibiting a response at the onset of the pinch were classified as Type 1, whereas neurons responding at the end of the pinch were classified as Type 2. Experimenters were blinded to the mice's genotype during recording.

#### *Ex vivo* electrophysiology patch‐clamp recordings

Male mice were deeply anaesthetized by an i.p. injection of a mix of ketamine (150 mg/kg Virbac 1000) and xylazine (60 mg/kg, Rompun 2%, Elanco, France). Coronal midbrain sections (250 µm) were sliced with a Compresstome (VF‐200, Precisionary Instruments, Allauch, France) after intracardial perfusion of cold (4°C) sucrose‐based ACSF containing (in mM): 125 NaCl, 2.5 KCl, 1.25 NaH_2_PO_4_, 26 NaHCO_3_, 5.9 MgCl_2_, 25 sucrose, 2.5 glucose and 1 kynurenate (pH 7.2, 325 mOsm). After 8 mins at 37°C for recovery, slices were transferred into oxygenated ACSF containing (in mM): 125 NaCl, 2.5 KCl, 1.25 NaH_2_PO_4_, 26 NaHCO_3_, 2 CaCl_2_, 1 MgCl_2_, 15 sucrose and 10 glucose (pH 7.2, 325 mOsm) at room temperature for the rest of the day. Slices were individually transferred to a recording chamber continuously perfused at 2 mL/min with oxygenated ACSF. Patch pipettes (4–6 MΩ) were pulled from thin wall borosilicate glass (G150TF‐3, Warner Instruments) with a micropipette puller (P‐87, Sutter Instruments Co., WPI, UK) and filled with a potassium gluconate‐based intracellular solution containing (in mM): 135 K‐gluconate, 10 HEPES, 0.1 EGTA, 5 KCl, 2 MgCl_2_, 2 ATP‐Mg, 0.2 GTP‐Na, and biocytin 2 mg/mL (pH adjusted to 7.2). Neurons were visualized using an upright microscope coupled with a dodt gradient contrast imager, and illuminated with a white light source (Scientifica, UK). Whole‐cell recordings were performed with a patch‐clamp amplifier (Axopatch 200B, Molecular Devices) connected to a Digidata (1550 LowNoise acquisition system, Molecular Devices). Signals were low‐pass filtered (Bessel, 2 kHz) and collected at 10 kHz using the data acquisition software pClamp 10.5 (Molecular Devices). VTA location was identified under a microscope. Identification of DA‐neurons was performed by location and by their electrophysiological properties [width and shape of AP and after‐hyperpolarization (AHP)]. Recorded neurons in patch‐clamp experiments were filled with biocytin in order to validate the presence of TH by immunofluorescence as an indicator of DA‐neurons.

### Fibre photometry

#### Stereotaxic surgeries

Mice received s.c. buprenorphine (0.1 mg/kg) and lidocaine before surgery and were anaesthetized with an isoflurane/oxygen gas mixture (induction: 3% isoflurane, 1 L/min O_2_; maintenance: 1% isoflurane) and placed in a stereotaxic frame (David Kopf Instruments, Phymep, France). Viral injections were performed using a glass micropipette (10 µL borosilicate glass capillary, Drummond, MultiChannelSystems, Germany) prefilled with mineral oil and mounted on a one‐axis oil hydraulic micromanipulator (MO‐10, Narishige). Mice received a unilateral injection of 0.4 µL AAV‐hSyn‐GRAB_DA3m_ (Sun et al., 2018) into the lateral shell of the Acb (coordinates relative to bregma: AP +1.45 mm, ML ±1.75 mm, DV −4.0 mm). An optical fibre (200 µm core diameter, NA = 0.38, Thorlabs, NJ, USA) coupled to a stainless‐steel ferrule (1.25 mm) was implanted 0.1 mm above the injection site (DV −3.90 mm) and secured to the skull with dental cement (Superbond, Sun Medical, Phymep). Post‐operative analgesia consisted of buprenorphine (0.1 mg/kg, s.c.) for 1–2 days, with additional treatment provided as needed. Animals were monitored for pain, distress, infection, weight loss and motor impairment, and were killed if predefined endpoint criteria were met. Mice were allowed to recover for a minimum of 4 weeks prior to any experimental procedure to ensure adequate viral expression. All recordings were performed on awake, freely moving mice.

#### Recordings

Fibre photometry signals were acquired at 100 Hz using a lock‐in detection system (Doric Lenses, Canada) and saved in the proprietary Doric format (HDF5). Two excitation channels were simultaneously recorded: a signal channel (470 nm, reflecting GRAB_DA_‐dependent dopamine fluctuations) and an isosbestic control channel (405 nm, insensitive to dopamine‐dependent fluorescence changes and used to account for motion artifacts and non‐neuronal noise). Raw fluorescence traces and corresponding time vectors were extracted from the Doric files using the h5py library in Python and organized into time‐series data tables. Injection of levodopa (l‐DOPA) was used to assess the specificity of dopamine‐dependent changes in the 470 nm GRAB_DA_ signal relative to the 405 nm control channel. During these experiments, the 470 nm GRAB_DA_ signal and the 405 nm channel were independently processed using the same monoexponential photobleaching correction pipeline and normalized as Δ*F*/*F*. The resulting signals were then directly compared to distinguish DA‐dependent fluorescence changes detected at 470 nm from non‐specific signal fluctuations measured at 405 nm. Because 405 nm does not correspond to a true isosbestic excitation wavelength for the GRAB_DA3m_ sensor, increases in dopamine levels induced by l‐DOPA produced a small decrease in 405 nm fluorescence. Therefore, the 405 nm channel was acquired as a reference to monitor non‐specific fluorescence fluctuations but was not used to normalize the 470 nm signal.

#### Photobleaching correction

The slow fluorescence decay caused by photobleaching was corrected by fitting a monoexponential model of the form:

$$y\left(t\right)=m\cdot e^{-\tau t}+b$$

to the pre‐injection baseline period (2400 s prior to injection) using scipy.optimize.curve_fit. The parameters *m* (initial amplitude), τ (decay constant) and *b* (asymptotic offset) were estimated from this baseline window, and the resulting decay curve was extrapolated over the full recording duration. The corrected signal was then computed as:

$$Fcorr\left(t\right)=Fraw\left(t\right)-y\left(t\right)+Fbaseline$$

Goodness of fit was assessed using the coefficient of determination *R* ^2^ and the Pearson correlation coefficient (*r*) computed between the observed baseline and the fitted curve.

#### Δ*F*/*F* normalization

The normalized fluorescence signal was computed as:

$$\Delta F/F\left(t\right)=\left(F\left(corr\left(t\right)\right)-F_{0}\right)/F_{0}$$

where *F*_0_ is the mean of the corrected signal over a 300 s reference window immediately preceding drug injection (−300 s to 0 s relative to injection onset). All timestamps were re‐centred on the injection time (*t* = 0). Post‐injection analysis windows were defined independently for each pharmacological condition according to the onset of the response observed in *ct* mice and were used to calculate the mean Δ*F*/*F* change relative to baseline: eticlopride [+225 s to +825 s], quinpirole [0 s to +240 s] and levodopa [+200 s to +1200 s].

#### Experimental design

All drugs were administered intraperitoneally (i.p.) in a volume of 0.2 mL per mouse. Sessions were separated by at least several days to minimize carry‐over effects. For each session, mice were connected to the fibre photometry system and allowed a brief habituation period before recording onset. *Session 1: eticlopride*. A saline injection (0.9% NaCl, i.p.) was administered 10 mins after the start of recording, followed by eticlopride (D2/D3 receptor antagonist, 1 mg/kg, i.p., Tocris) 30 mins later. GRAB_DA_ fluorescence was recorded continuously throughout the session. *Session 2: quinpirole/eticlopride*. Following the same saline pre‐injection 10 mins after recording onset, quinpirole (D2/D3 receptor agonist, 1 mg/kg, i.p., Tocris) was administered 30 mins later. At the end of the quinpirole recording window, eticlopride (1 mg/kg, i.p., Tocris) was injected to pharmacologically reverse dopamine receptor activation. GRAB_DA_ fluorescence was recorded continuously throughout the session. *Session 3: carbidopa/l‐DOPA*. Carbidopa (peripheral DOPA decarboxylase inhibitor, 12 mg/kg, i.p., Sigma‐Aldrich) was administered 10 mins after recording onset to prevent peripheral conversion of levodopa. Levodopa (10 mg/kg, i.p., Sigma‐Aldrich) was injected 30 mins later, and the GRAB_DA_ signal was recorded for approximately 40 mins following l‐DOPA administration. At the end of each session, mice were disconnected from the patch cord and returned to their home cage.

### Behaviours

#### Locomotor activity

Horizontal and vertical locomotor activity are measured in a circular corridor (14 cm wide, 18 cm in diameter) with four infrared beams placed at 90° angles (Imetronic, Pessac, France) in a low luminosity environment.

#### Drug administration protocols

*Acute drug administration*. Mice were habituated to the activity box, handling and procedure for 90 or 120 min for two consecutive days before the actual experiment. During habituation, spontaneous locomotor activity was recorded for 30 min before the mice were administered with a saline solution and placed again in the circular corridor for 60 min. The same procedure occurred during the test day, except that saline solution was replaced by either quinpirole, cocaine, methylphenidate, SKF81297, GBR12783 or d‐amphetamine. *Repeated d‐amphetamine administration*. Mice were habituated to locomotor activity boxes, as described above, for 2 days and then treated daily with d‐amphetamine (1.5 mg/kg, i.p.) for five consecutive days. Following 10 days of withdrawal, mice were challenged with d‐amphetamine.

#### Catalepsy

Mice were treated with eticlopride (0.5 and 1 mg/kg, i.p.) and catalepsy was assessed using the bar test. The time spent in a cataleptic position was evaluated at various intervals after administration. Cutoff was fixed at 120 s.

#### Elevated plus maze

This test was performed as previously described (Castell et al., 2024). The number of entries and time spent in the closed arms (four paws within the closed arm) or open arms (four paws within the open arms) were calculated during 5 min.

#### Hotplate test

Thermal nociception was assessed using a hotplate. Mice were place in plexiglass chamber (17 × 17 × 25 cm) on a hotplate at 52°C. The latency to lick the front paws was recorded using a stopwatch.

#### Sugar preference test

Before each preference test, mice were pre‐exposed to solutions containing either sucrose (10% w/v) or saccharin (0.1% w/v) to reduce neophobia to sweetened solutions and delivery systems (lickometers). For the preference test, mice were presented with two pre‐filled lickometers for a period of 120 min, allowing the assessment of sugar preference (consumption) under the following conditions: sucrose *versus* water, saccharin *versus* water, or sucrose *versus* saccharin. Consumption (mL) was measured every 15 min. To control for potential side bias, the position of water, sucrose and saccharin solutions was alternated within and between preference tests.

#### Discriminative auditory threat conditioning

The experiment was carried out in a soundproof fear conditioning apparatus (Imetronic) as previously described (Castell et al., 2024). The protocol spanned 4 days as follows: Day 1, *habituation*: after 5 min of exploration in box A or B, two different tones (CS+ and CS−; 30 s four times each) were randomly presented to the mice; *conditioning*: the same day, mice were placed on the same box as for the habituation and after 5 min of exploration they received five pairings of the CS+ (30 s) with the foot‐shock at the last second of the CS+ (1 s, 0.7 mA), and five presentations of the CS− alone (30 s). Inter‐trial intervals lasted randomly between 20 and 180 s. Days 2–4: animals underwent the test (day 2) and two extinction sessions (days 3–4) in a different box during three consecutive days. During these sessions, animals received 12 presentations of the CS+ (30 s) and four presentations of the CS−. The inter‐trial interval in all sessions was between 20 and 180 s. During the habituation and conditioning phases, cages were cleaned with 70% ethanol while for the test and extinction sessions they were cleaned with 1% acetic acid. The meaning of each tone (pairing or not with the foot‐shock) was counterbalanced between animals and genotypes. Freezing behaviour was recorded using a tight infrared frame. The threshold for considering freezing behaviour was set up at 2 s. The first 10 min of habituation was used to assess the basal freezing. Data are presented as the probability to evoke freezing behaviour during CS+ and CS− presentation.

#### Discriminative active avoidance

The experiment was carried out in a soundproof shuttle box (Imetronic) as previously described (Castell et al., 2024). The apparatus consists of two equal compartments (20 × 20 cm) separated by an opened door. Both compartments have a metal grid on the floor, independent houselights and one infrared beam frame on each compartment. Day 1: mice were subjected to a habituation session consisting of 10 presentations of two different tones, a CS− and CS+ lasting 13 s maximum. Subjects can stop both CSs by shuttling from one compartment to another. Days 2–4: mice were subjected to the training sessions once per day during which they were exposed to CS+/CS− in a pseudo‐random manner. Again, the CS (+ or−) stops whenever the mice cross from one compartment to another. CS− last 8 s maximum. CS+ last 13 s maximum, but after 8 s the unconditioned stimulus (0.4 mA foot‐shock coinciding with the last 5 s of the CS+ presentation) is delivered until the mice shuttle or for a maximum of 5 s. During the inter‐trial intervals, the animal was free to cross between compartments. The probability of avoidances was used as an index of learning.

### Statistical analysis

All statistical analyses for electrophysiological recordings were performed using the R software (version 4.4.1) with home‐made routines. Results were plotted as mean ± SD. The total number (*n*) of observations in each group and the statistical tests used for comparisons between groups or within groups are indicated directly on the figures or provided in figure legends. Comparisons between distributions were performed with the Kolmogorov–Smirnov test. Comparisons between means were performed with parametric tests such as Student's *t* test, or two‐way ANOVA for comparing two groups when parameters followed a normal distribution (Shapiro–Wilk normality test with *P* > 0.05), or Wilcoxon non‐parametric test if the distribution was skewed. Western blots were analysed with an unpaired Student *t* test. Behaviours and microdialysis experiments were analysed with two‐way repeated‐measures ANOVA, and an unpaired or paired Student *t* test. For fibre photometry experiments, individual Δ*F*/*F* traces were aggregated by genotype at each time point and group. Statistical comparisons were performed on the per‐animal mean Δ*F*/*F* averaged over the post‐injection analysis window. Normality of distributions was assessed using the Shapiro–Wilk test. When normality could not be assumed, a non‐parametric Mann–Whitney U test was applied for independent‐group comparisons. For within‐subject comparisons (e.g. saline *vs*. drug within the same animal), a paired *t* test or Wilcoxon signed‐rank test was used depending on the normality of pairwise differences. All statistical analyses were performed in Python using scipy.stats. Statistical significance was set at *P < 0.05, **P < 0.01 and ***P < 0.001. More details are given in the appendix.

## Results

### Midbrain DA‐neurons express the sodium leak channel NALCN

Recent evidence suggests that the sodium leak channel NALCN regulates midbrain DA‐neuron excitability (Cobb‐Lewis et al., 2023; Hahn et al., 2023; Philippart & Khaliq, 2018; Um et al., 2021). Using *in situ* hybridization, we first evaluated the percentage of cells expressing *Nalcn* transcripts in VTA DA‐neurons identified by *Slc6a3* and *Th* transcripts in both male and female C57BL/6J mice (Fig. 1*A*). We found that 100% of *Slc6a3‐* and *Th*‐positive cells expressed *Nalcn* transcripts (Fig. 1*A*). Of note, VTA GABA‐neurons also contained *Nalcn* transcripts, thus illustrating the ubiquitous expression of NALCN in the VTA.

**Figure 1:**
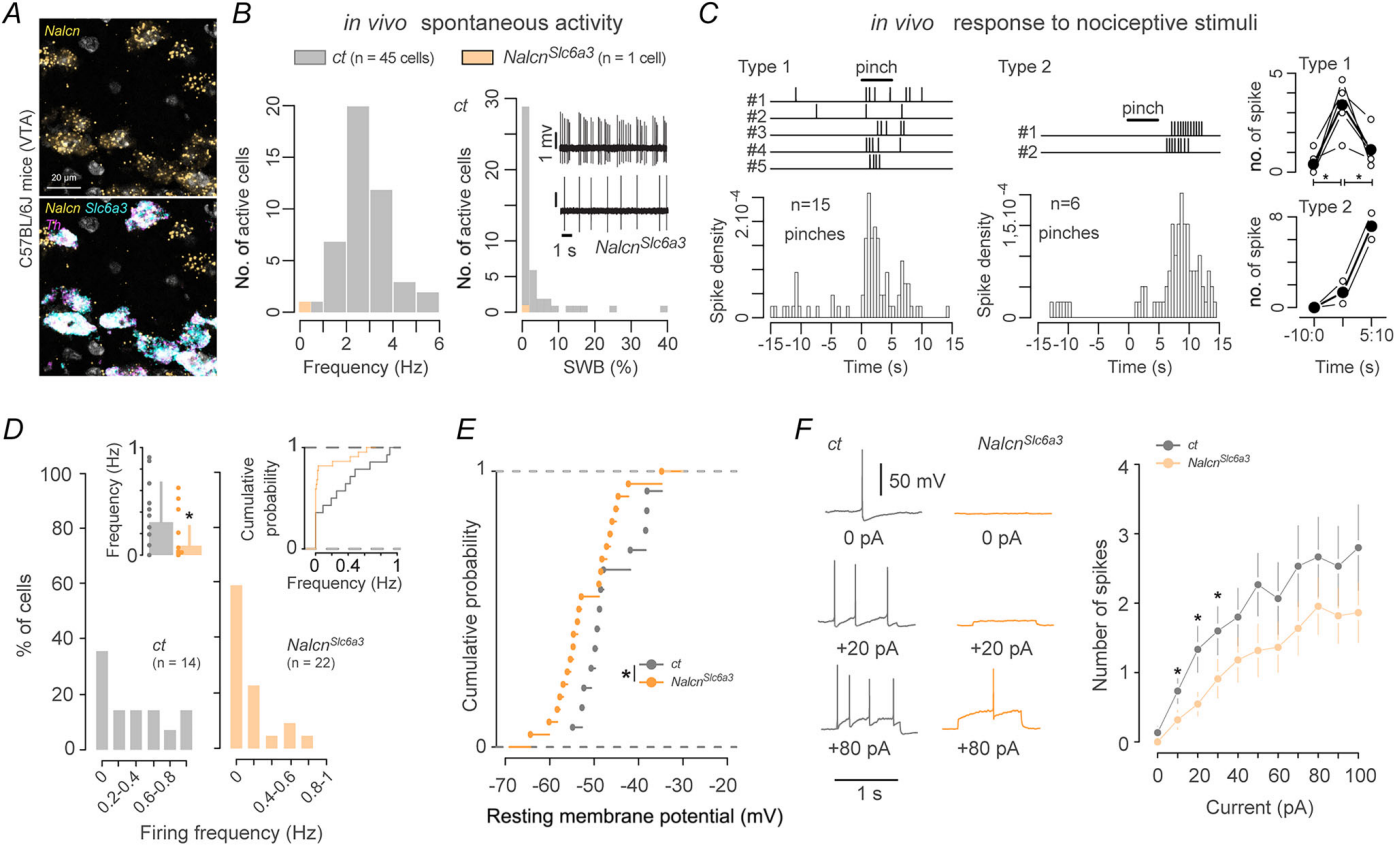
Characterization of electrophysiological properties of VTA DA‐neurons lacking *Nalcn* *A*, high magnification of a representative confocal image of coronal brain section of the VTA from C57BL/6J mice (*n* = 3 mice) showing *Th* (magenta), *Nalcn* (orange) and *Slc6a3* (cyan) mRNA expression detected with single‐molecular fluorescence *in situ* hybridization. Slides were counterstained with DAPI (white). *B*, *in vivo* juxtacellular recordings of putative DA neurons on anaesthetized animals. Distribution of spontaneous firing frequency among active putative DA neurons in the VTA of *ct* (grey, *n* = 45 neurons of 2 mice) and *Nalcn^Slc6a3^* (orange, *n* = 1 neuron of 2 mice) mice. pDA of *Nalcn^Slc6a3^* mice are silent except one neuron, compared to putative DA‐neurons of *ct* mice showing a frequency of up to 6 Hz and bursting activity. *C*, *in vivo* response to nociceptive stimuli of silent putative DA neurons on anaesthetized *Nalcn^Slc6a3^* mice. Left: raster plot of AP evoked during a 5 s tail pinch in five neurons (Type 1) and cumulative density of AP 15 s before and after tail pinch (15 pinches, 3 pinches per neuron). Middle: raster plot of AP evoked at the end a 5 s tail pinch in two neurons (Type 2) and cumulative density of AP 15 s before and after tail pinch (6 pinches, 3 pinches per neuron). Right: mean number of AP, 10 s before, during or 5 s after the pinch on Type 1 (upper) or Type 2 (lower) neurons. White dot: mean number of AP per neuron for three pinches. Black dot: mean number of AP on five Type 1 and two Type 2 neurons. *D*, *in vitro* recordings of *ct* (grey, *n* = 14 neurons of 2 mice) and *Nalcn^Slc6a3^* (orange, *n* = 22 neurons of 3 mice) mice, Wilcoxon test *P* = 0.046. Histogram density of firing rates with corresponding cumulative probability (inset). *E*, cumulative probability of resting membrane potential, Kolmogorov–Smirnov test *P* = 0.028. *F*, left; example traces of firing of VTA DA‐neurons of *ct* and *Nalcn^Slc6a3^* mice after current injections (0, +20, +80 pA). Right: *F–I* curve (frequency–current relationship). Lower excitability of VTA DA neurons of *Nalcn^Slc6a3^* mice for no or small currents (0, 10, 20, 30 pA) compared to DA neurons of *ct* mice, Wilcoxon test: 10 pA, *P* = 0.015; 20 pA, *P* = 0.017; 30 pA, *P* = 0.032. *P < 0.05.

### Selective deletion of *Nalcn* in midbrain DA‐neurons alters pacemaker activity

To explore the role of NALCN in regulating the firing patterns of midbrain DA‐neurons, we generated mice with a selective deletion of *Nalcn* in midbrain DA‐neurons by crossing *Nalcn^loxP/loxP^* mice with the *Slc6a3*‐Cre mouse line (referred to as *Nalcn^Slc6a3^* mice). The *in vivo* electrophysiological activity of VTA DA‐neurons was evaluated using juxtacellular recordings in anaesthetized mice. Strikingly, while we recorded a substantial number of active cells in control (*ct*) mice (*n* = 45, on two male mice), spontaneously active neurons were difficult to observe in *Nalcn^Slc6a3^* male mice (only one active neuron, below 1 Hz, on  2 mice) suggesting that in anesthetized mice, the majority of DA‐neurons lacking *Nalcn* are silent (Fig. 1*B*). Nevertheless, following repeated tail pinches, we were able to evoke excitatory responses in seven previously silent putative DA‐neurons across *Nalcn^Slc6a3^* male mice. Five neurons responded by firing action potentials (APs) during the stimulus (Type 1), while two fired APs at the end of the stimulus (Type 2) indicating that the response of VTA DA‐neurons to aversive stimuli was still present in *Nalcn^Slc6a3 ^* mice (Fig. 1*C*). Because our previous observation precluded a more in‐depth *in vivo* characterization, we then employed *ex vivo* patch‐clamp recordings to compare the pacemaker activity of DA‐neurons between *ct* and *Nalcn^Slc6a3^* male mice. Consistent with previous observations (Cobb‐Lewis et al., 2023), VTA DA‐neurons of *Nalcn^Slc6a3^* male mice exhibited reduced spontaneous activity (mean *ct* = 0.3 Hz, mean *Nalcn^Slc6a3^* = 0.08 Hz, Fig. 1*D*) and a more hyperpolarized resting membrane potential as compared to *ct* (mean *ct* = −46 mV, mean *Nalcn^Slc6a3^* = −51 mV, Fig. 1*E*). Regarding membrane properties, the current–response curve showed a reduced excitability of DA‐neurons in *Nalcn^Slc6a3^* male mice as compared to *ct* (Fig. 1*F*).

### Characterization of striatal DA signalling in *Nalcn^Slc6a3^* mice

To determine whether reduced activity of VTA DA‐neurons translates into altered extracellular DA levels, we performed *in vivo* microdialysis in the Acb of *Nalcn^Slc6a3^* and *ct* mice. Basal accumbal DA levels did not differ between genotypes, although a trend toward reduction was observed in *Nalcn^Slc6a3^* male mice (Fig. 2*A*). Next, we examined the expression of key components of DA signalling within the striatum. We performed immunofluorescence analyses and western blots to measure the expression of the dopamine transporter (DAT), TH, and vesicular monoamine transporter 2 (VMAT2) in the striatum of both sexes and genotypes. DAT, TH and VMAT2 levels remained unchanged in the striatum of *Nalcn^Slc6a3 ^* mice (Fig. 2*B*–*E*). Moreover, analyses of the phosphorylated forms of TH at Ser31 and Ser40 (pS31‐TH and pS40‐TH), used as a proxy of the enzyme's activity (Lindgren et al., 2000, 2001), revealed no differences between the two genotypes, further supporting the idea that DA production is not impaired in *Nalcn^Slc6a3^* mice (Fig. 2*F*). Finally, male and female *Nalcn^Slc6a3^* mice displayed similar spontaneous locomotion and exploratory drive as compared to *ct* (Fig. 2*G*).

**Figure 2:**
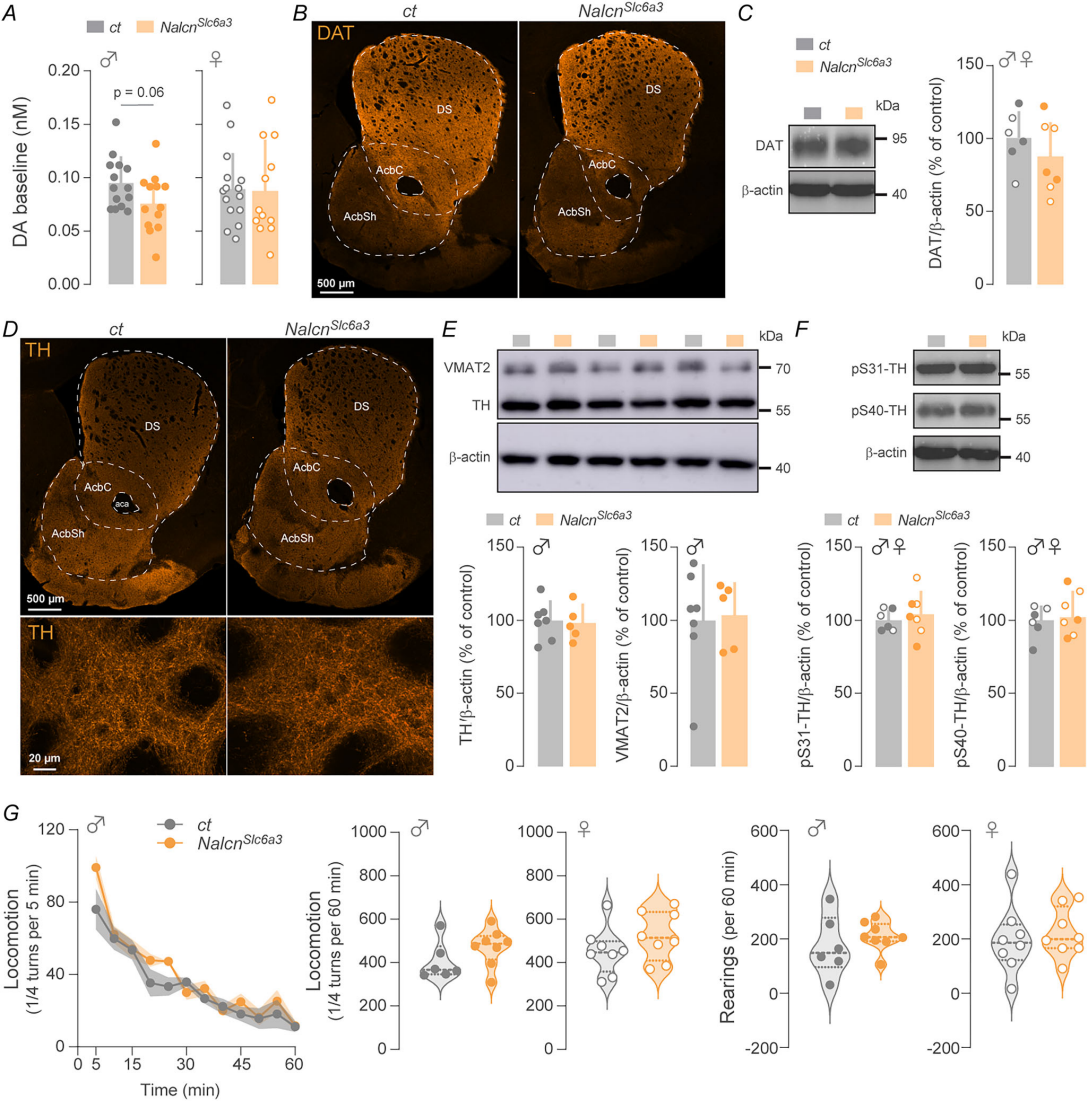
Extracellular DA levels in the nucleus accumbens and characterization of striatal expression of key components of DA signalling in *Nalcn^Slc6a3^* mice *A*, quantifications of extracellular DA levels of both male (full circle) and female (empty circle) *ct* (male, *n* = 14; female, *n* = 15) and *Nalcn^Slc6a3^* mice (male, *n* = 13; female, *n* = 12), male *ct vs*. male cKO: unpaired *t* test *P* = 0.066; female *ct vs*. female cKO unpaired *t* test *P* = 0.862. *B*, immunofluorescence detection of the dopamine transporter (DAT) in striatal sections of *ct* (left) and *Nalcn^Slc6a3^* (right). *C*, representative immunoblots and quantification of DAT expression in *ct* (male, *n* = 3; female, *n* = 3) and *Nalcn^Slc6a3^* (male, *n* = 3; female, *n* = 4) mice, unpaired *t* test *P* = 0.324. *D*, coronal striatal sections showing expression of tyrosine hydroxylase (TH) in *ct* (left panel) and *Nalcn^Slc6a3^* (right panel) mice. *E*, representative immunoblots and quantification of TH, unpaired *t* test *P* = 0.558 and VMAT2 expressions in *ct* (*n* = 7) and *Nalcn^Slc6a3^* (*n* = 5) mice, unpaired *t* test *P* = 0.846. *F*, representative immunoblots and quantification of phosphorylated form of TH on residue Ser31 (pS31‐TH) in *ct* (male, *n* = 3; female, *n* = 4) and *Nalcn^Slc6a3^* (male, *n* = 3; female, *n* = 3) mice, unpaired *t* test *P* = 0.838 and on Ser40 (pS40‐TH) and *ct* (male, *n* = 3; female, *n* = 3) and *Nalcn^Slc6a3^* (male, *n* = 3; female, *n* = 3) mice, unpaired *t* test *P* = 0.787. *G*, horizontal (locomotion) and vertical (rearing) locomotor activity in *Nalcn^Slc6a3^* (male, *n* = 6; female, *n* = 8) mice compared to *ct* mice (male, *n* = 6; female, *n* = 8). Male *ct vs*. male cKO, locomotion: unpaired *t* test *P* = 0.172; rearing: unpaired *t* test *P* = 0.328. Female *ct vs*. female cKO, locomotion: unpaired *t* test *P* = 0.216; rearing: unpaired *t* test *P* = 0.646. Abbreviations: aca, anterior commissure; AcbC, nucleus accumbens core; AcbSh, nucleus accumbens shell; DS, dorsal striatum.

### D2R‐mediated signalling at DA terminals is impaired in *Nalcn^Slc6a3^* mice

A previous study has indicated that NALCN is involved in D2R‐mediated modulation of DA‐neurons (Philippart & Khaliq, 2018). We examined the presynaptic *versus* postsynaptic functions of D2R in response to pharmacological stimulations. First, we analysed the expression levels of D2R by immunofluorescence and found no alterations in the striatum of *Nalcn^Slc6a3^* mice (Fig. 3*A*). We then analyszed the dose‐dependent effects of the D2R agonist quinpirole (0.05, 0.5 and 1 mg/kg, i.p.) on locomotor activity (Fig. 3*B*,*C*). We found that quinpirole was less effective in decreasing the locomotor activity of *Nalcn^Slc6a3^* mice as compared to *ct*, suggesting that the function of presynaptic D2R onto DA‐neurons might have been impaired. To test this hypothesis, we evaluated the ability of the D2R agonist (quinpirole, 1 mg/kg, i.p.) and antagonist (eticlopride, 1 mg/kg, i.p.) to modulate DA dynamics using *in vivo* fibre photometry coupled to the DA biosensor GRAB_DA3m_ following injection of an AAV‐hSyn‐GRAB_DA3m_ virus into the lateral shell of the Acb of *ct* and *Nalcn^Slc6a3^* mice from both sexes (Fig. 3*D*–*F*). To validate the DA specificity of the GRAB_DA3m_ signal, fluorescence recorded at 470 nm excitation was compared with the 405 nm reference channel following l‐DOPA administration. As expected, l‐DOPA induced a significant increase in 470 nm fluorescence, whereas no increase was observed in the 405 nm channel (Supporting information). We first validated that the administration of the D2R agonist quinpirole (1 mg/kg, i.v.), known to induce a marked decrease in the firing frequency of VTA DA‐neurons in anaesthetized mice, was efficient to decrease Acb DA transients assessed in awake mice, whereas subsequent eticlopride administration restored it to baseline levels (Fig. 3*D*). The close correspondence between the effects of D2 receptor pharmacology on DA‐neuron firing and GRAB_DA3m_ signals supports the interpretation that the recorded fluorescence fluctuations reflect changes in DA release rather than a direct interaction of these drugs with the sensor and depend on action potential‐driven neuronal activity. Next, in a separate set of experiments, we confirmed that quinpirole and eticlopride respectively decreased and increased DA release relative to saline injections in both genotypes (Supporting information). Interestingly, the magnitude of the decrease of Acb DA transients induced by quinpirole was significantly reduced in *Nalcn^Slc6a3^* mice as compared to *ct* (Fig. 3*E*). Conversely, eticlopride‐induced DA release in the Acb, evaluated by fibre photometry or microdialysis, was markedly reduced in *Nalcn^Slc6a3^* male and female mice (Fig. 3*F*,*G*). Finally, the ability of a D2R antagonist (eticlopride, 0.5 mg/kg, i.p.) to promote the increase of TH phosphorylation at Ser40 (pS40‐TH) in the Acb was absent in *Nalcn^Slc6a3^* mice compared to *ct* (Fig. 3*H*), suggesting that D2R‐mediated signalling of DA transmission is impaired in *Nalcn^Slc6a3^* mice. Despite these results, the possibility that the altered pacemaker activity previously observed in electrophysiological recordings also contributes indirectly to these effects cannot be excluded. Importantly, quinpirole still induced a significant reduction in the GRAB_DA3m_ signal in *Nalcn^Slc6a3^* mice. Because activation of autoD2R reduces DA release, such an effect could only be observed if DA was being tonically released before drug administration. These results therefore demonstrate the persistence of tonic DA tone in awake *Nalcn^Slc6a3^* mice despite the profound alterations in DA‐neuron activity observed under anaesthetized conditions.

**Figure 3:**
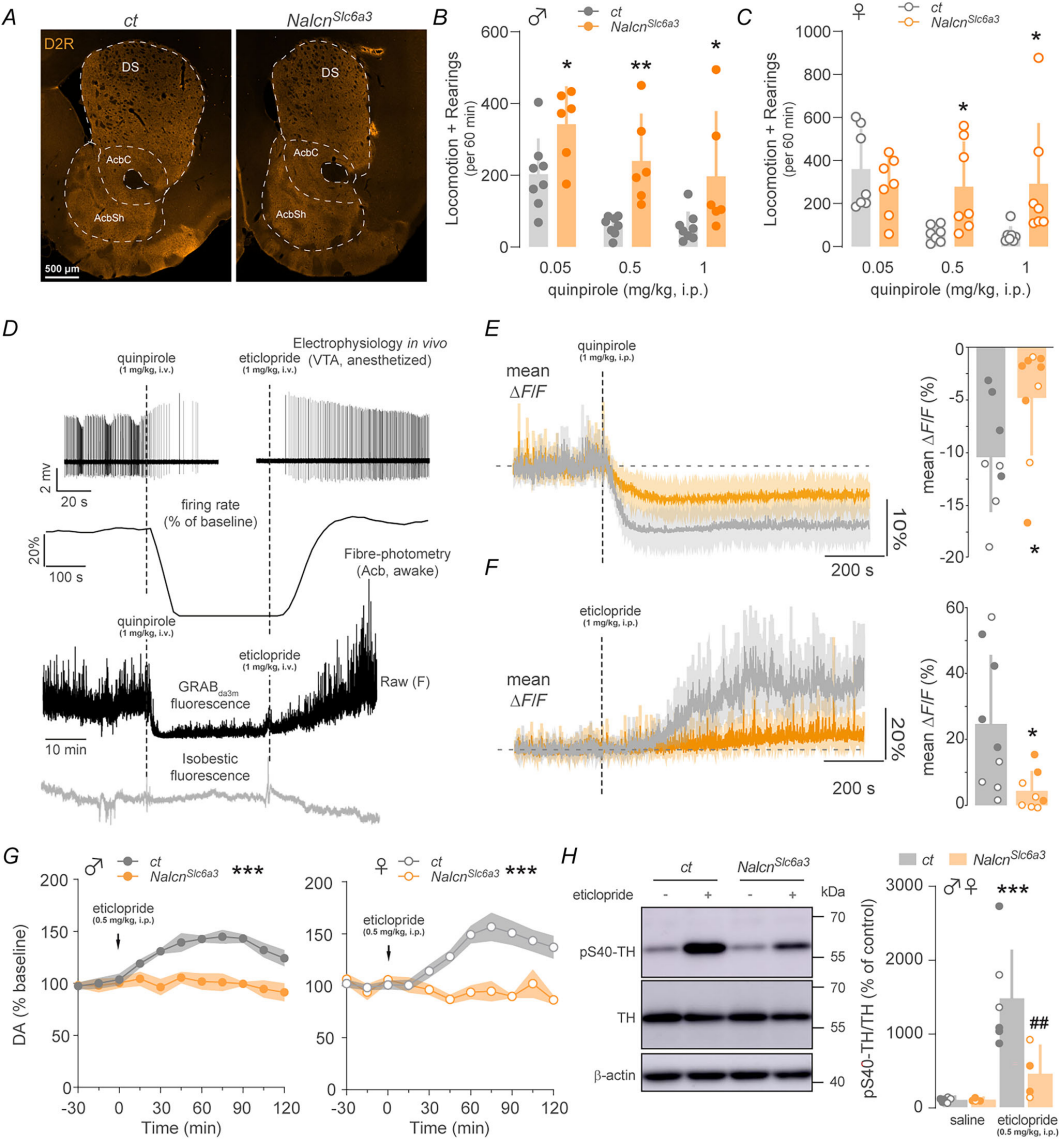
Pharmacological stimulation of D2R in mice lacking *Nalcn* in DA‐neurons *A*, coronal sections at the striatum level showing expression of D2R in *ct* and *Nalcn^Slc6a3^* (mice. *B* and *C*, horizontal locomotor activity after an acute injection of quinpirole (0.05, *P* = 0.036; 0.5, *P* = 0.006 and 1 mg/kg *P* = 0.041) in male (*B*) mice (*ct*, *n* = 8; *Nalcn^Slc6a3^*, *n* = 6) and in female (*C*) mice (*ct*, *n* = 8; *Nalcn^Slc6a3^*, *n* = 7), 0.05, *P* = 0.406; 0.5, *P* = 0.045 and 1 mg/kg *P* = 0.027. *D*, upper: representative *in vivo* juxtacellular recording illustrating changes in the firing activity of a VTA DA‐neuron following administration of quinpirole (1 mg/kg, i.v.) and eticlopride (1 mg/kg, i.v.) in anaesthetized *ct* mice. Lower: representative GRAB_DA3m_ fluorescence recordings at 470 and 405 nm excitation wavelengths obtained by fibre‐photometry in the Acb following administration of quinpirole (1 mg/kg, i.p.) and eticlopride (1 mg/kg, i.p.) in awake *ct* mice. Data are presented as raw fluorescence signals without any post‐processing, normalization or baseline correction. *E*, mean ∆*F*/*F* change of GRAB_DA3m_ fluorescence (470 nm) in the Acb following quinpirole injection (1 mg/kg, i.p) in *ct* (*n* = 9, grey) or *Nalcn^Slc6a3^* (*n* = 8, orange) mice. Mann–Whitney, *P* = 0.027. *F*, mean ∆*F*/*F* change of GRABDA3m fluorescence (470 nm) in the NAc following eticlopride injection (1 mg/kg, i.p) in *ct* (*n* = 9, grey) or *Nalcn^Slc6a3^* (*n* = 8, orange) mice. Unpaired *t* test, *P* = 0.018. *G*, accumbal extracellular level of DA after a single administration of eticlopride (0.5 mg/kg, i.p.) in male mice (left panel) (*ct, n = *5; *Nalcn^Slc6a3^*, *n* = 5). Two‐way repeated‐measures (RM) ANOVA, time *F*_(11, 88)_ = 5.187, *P* < 0.0001; genotype *F*_(1, 8)_ = 67.10, *P* < 0.0001; interaction *F*_(11, 88)_ = 5.026, *P* < 0.0001; and female mice (right panel) (*ct, n *= 4; *Nalcn^Slc6a3^*, *n* = 5). Two‐way RM ANOVA, time *F*_(11, 77)_ = 4.578, *P* < 0.0001; genotype *F*_(1, 7)_ = 24.15, *P* = 0.0017; interaction *F*_(11, 77)_ = 7.491, *P* < 0.0001. *H*, western blot analysis of pS40‐TH in the striata of *ct* (male, saline *n* = 3: eticlopride, *n* = 4; female, saline *n* = 3: eticlopride, *n* = 2) and *Nalcn^Slc6a3^* (male, saline *n* = 3: eticlopride, *n* = 2; female, saline *n* = 1: eticlopride, *n* = 2) mice treated with saline or eticlopride and killed after 15 min. Two‐way RM ANOVA, treatment *F*_(1, 16)_ = 20.41, *P* = 0.0004; genotype *F*_(1, 16)_ = 7.140, *P* = 0.018*; interaction *F*_(1, 16)_ = 7.190, *P* = 0.016. Abbreviations: AcbC, nucleus accumbens core; AcbSh, nucleus accumbens shell; DS, dorsal striatum. *P < 0.05, **P < 0.01, ***P < 0.001, ^##^ *P* < 0.01.

We then complemented our analysis by assessing whether the function of postsynaptic D2R was altered. To do so, we evaluated the cataleptic responses elicited by the D2R antagonism in *Nalcn^Slc6a3^* and *ct* mice (Anzalone et al., 2012). The administration of eticlopride (0.5 or 1 mg/kg, i.p.) induced dose‐dependent and long‐lasting cataleptic effects in *ct* and *Nalcn^Slc6a3^* mice of both sexes (Fig. 4*A*,*B*). Finally, we examined the regulation of the well‐established cellular responses induced by eticlopride (Bertran‐Gonzalez et al., 2009; Valjent et al., 2019). This included the postsynaptic induction of the immediate early gene FOS and the phosphorylation of histone H3 (pS10‐H3). Immunofluorescence analyses revealed that eticlopride (0.5 mg/kg, i.p.) equally increased the number of FOS‐neurons in the striatum of *ct* and *Nalcn^Slc6a3^* mice from both sexes (Fig. 4*C*,*D*). Similar results were obtained for pS10‐H3 analysed by immunofluorescence (Fig. 4*E*,*F*), suggesting that D2R‐mediated signalling in striatal D2R‐containing neurons is not altered.

**Figure 4:**
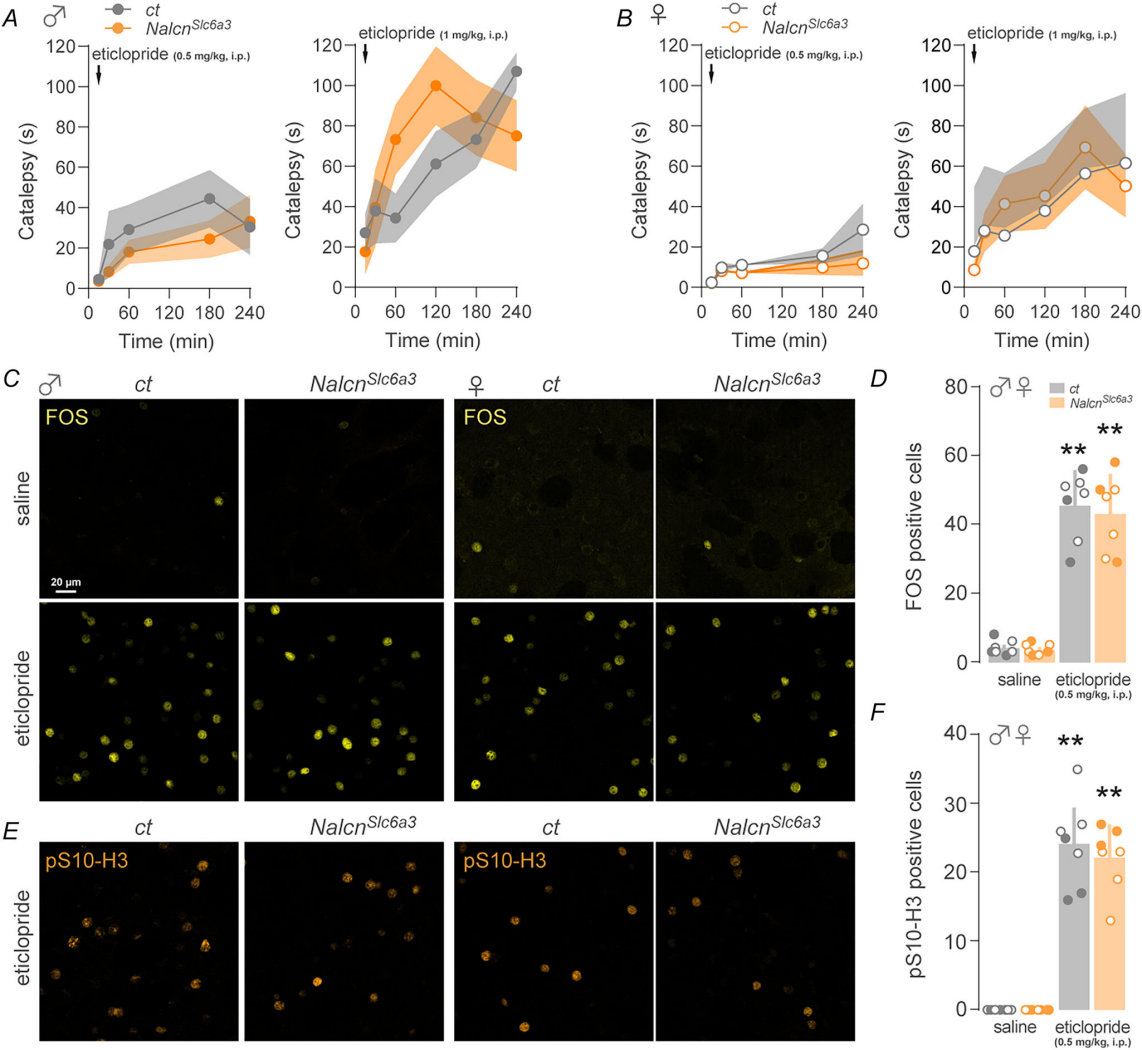
Eticlopride‐mediated motor responses and intracellular signalling in mice lacking *Nalcn* in DA‐neurons *A* and *B*, catalepsy determined 15, 30, 60, 180 and 240 min after administration of eticlopride at 0.5 mg/kg (*A* and *B*, left panels) or 1 mg/kg (*A* and *B*, right panels) in male (*A*) mice (*ct*, *n* = 7 and 7; *Nalcn^Slc6a3^*, *n* = 7 and 6). Two‐way RM ANOVA, 0.5 mg/kg: time *F*_(4, 48)_ = 4.099, *P* = 0.021, genotype *F*_(1, 12)_ = 0.7126, *P* = 0.415, interaction *F*_(4, 48)_ = 0.209, *P* = 0.684; 1 mg/kg: time *F*_(5, 55)_ = 12.17, *P* < 0.0001, genotype *F*_(1, 11)_ = 0.2512, *P* = 0.626, interaction *F*_(5, 55)_ = 3.218, *P* = 0.013; and female (*B*) mice (*ct*, *n* = 9 and 8; *Nalcn^Slc6a3^*, *n* = 7 and 6). Two‐way RM ANOVA, 0.5 mg/kg: time *F*_(4, 55)_ = 2.826, *P* = 0.033, genotype *F*_(1, 14)_ = 2.210, *P* = 0.159, interaction *F*_(4, 55)_ = 0.7210, *P* = 0.581; 1 mg/kg time *F*_(3.060,36.73)_ = 4.714, *P* = 0.007, genotype *F*_(1, 12)_ = 0.02804, *P* = 0.869, interaction *F*_(5, 60)_ = 0.4538, *P* = 0.809. *C* and *E*, confocal sections of the dorsal striata, showing immunofluorescence for FOS (*C*) and the phosphorylated form of histone H3 at Ser10 (*E*) (pS10‐H3) in *ct* and *Nalcn^Slc6a3^* male and female mice treated with saline or eticlopride and 60 min later. *D* and *F*, quantification of FOS (*D*). Two‐way ANOVA, treatment *F*_(1, 24)_ = 200.3, *P* < 0.0001, genotype *F*_(1, 24)_ = 0.2501, *P* = 0.622, interaction *F*_(1, 24)_ = 0.1226, *P* = 0.729; and pS10‐H3 (*F*) immunoreactive neurons in the dorsal striata of saline or eticlopride‐treated *ct* (male, saline *n* = 3: eticlopride, *n* = 3; female, saline *n* = 4: eticlopride, *n* = 4) and *Nalcn^Slc6a3^* (male, saline *n* = 3: eticlopride, *n* = 3; female, saline *n* = 4: eticlopride, *n* = 4) mice. Two‐way ANOVA, treatment *F*_(1, 24)_ = 269, *P* < 0.0001, genotype *F*_(1, 24)_ = 0.3159, *P* = 0.579, interaction *F*_(1, 24)_ = 0.3159, *P* = 0.579. **P < 0.01.

### Risk assessment and reactivity to threat‐predicting cues is unaffected in *Nalcn^Slc6a3^* mice

Midbrain DA‐neurons play a critical role in the attribution of motivational salience to aversive‐related information and the orchestration of aversive behaviours (Bromberg‐Martin et al., 2010; Duvarci, 2024). We first investigated whether NALCN signalling in midbrain DA‐neurons functionally modulates risk assessment. No differences between genotypes were detected in the time spent in the open arms in the elevated plus maze indicating that risk assessment was not impaired in *Nalcn^Slc6a3^* mice (Fig. 5*A*,*B*).

**Figure 5:**
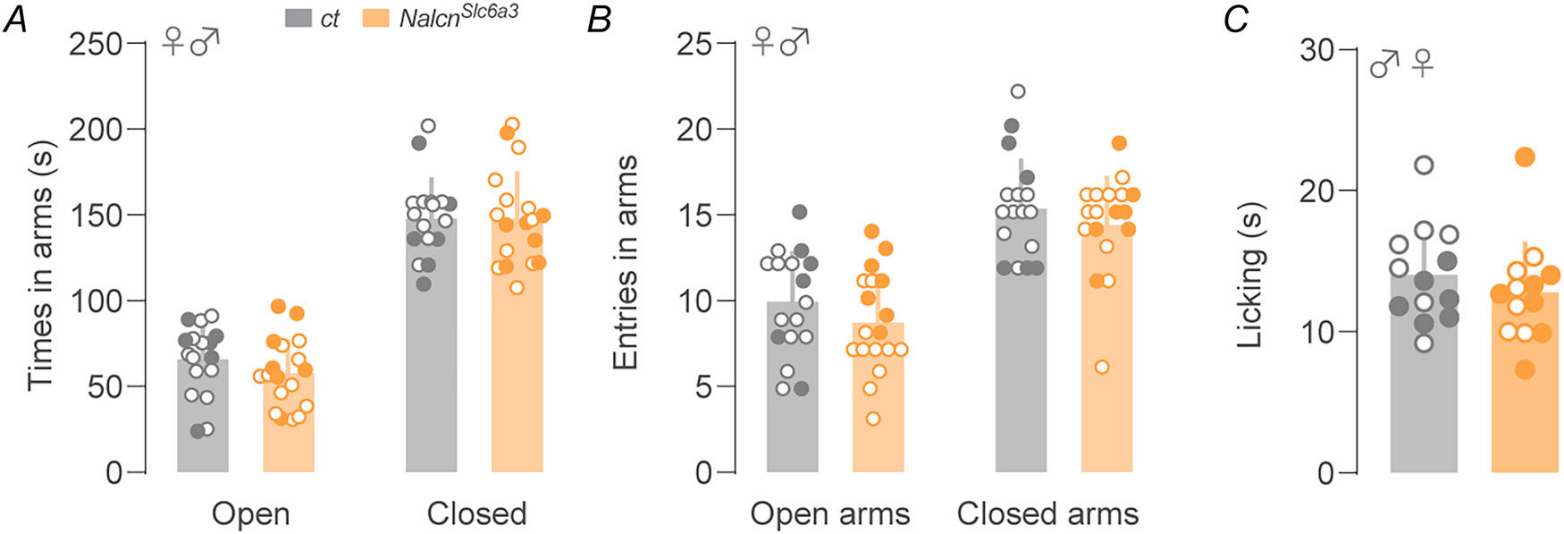
Risk‐assessment and nociceptive thresholds in male and female mice lacking *Nalcn* in DA‐neurons *A*, time spent in open and closed arms. Unpaired *t* test, open *P* = 0.254, closed *P* = 0.980. *B*, number of entries in open and closed arms during 5 mins in the elevated plus maze in females (upper panel*: ct*, *n* = 7, *Nalcn^Slc6a3^, n* = 7) and males (lower panels*: ct*, *n* = 6*, Nalcn^Slc6a3^*, *n* = 7). Unpaired *t* test, open *P* = 0.229, closed *P* = 0.330. *C*, latency to the first hind‐paw licking in the hotplate test in *ct* and *Nalcn^Slc6a3^* mice (female: *ct*, *n* = 7, *Nalcn^Slc6a3^, n* = 6; male: *ct*, *n* = 6*, Nalcn^Slc6a3^, n* = 7). Unpaired *t* test *P* = 0.380.

Before subjecting mice of both sexes and genotypes to discriminative auditory threat conditioning, we first ensured that nocifensive responses were not altered. No differences were found in the response latency of hind‐paw licking in the hotplate test, indicating that nociceptive thresholds were not impaired in *Nalcn^Slc6a3^* mice (Fig. 5*C*). On day 1 (conditioning day), *ct* and *Nalcn^Slc6a3^* mice (both sexes) expressed similar levels of freezing in response to CS+ and CS− (Fig. 6 *A*,*B*). On the test day (day 2), *ct* and *Nalcn^Slc6a3^* mice equally discriminated between auditory cues predicting (CS+) or not (CS−) threat (Fig. 6*C*, left panels). Following repeated CS+ presentation without shock (extinction), male and female mice from both genotypes gradually decreased their freezing responses, indicating that the extinction process was not altered (Fig. 6*C*, right panels).

**Figure 6:**
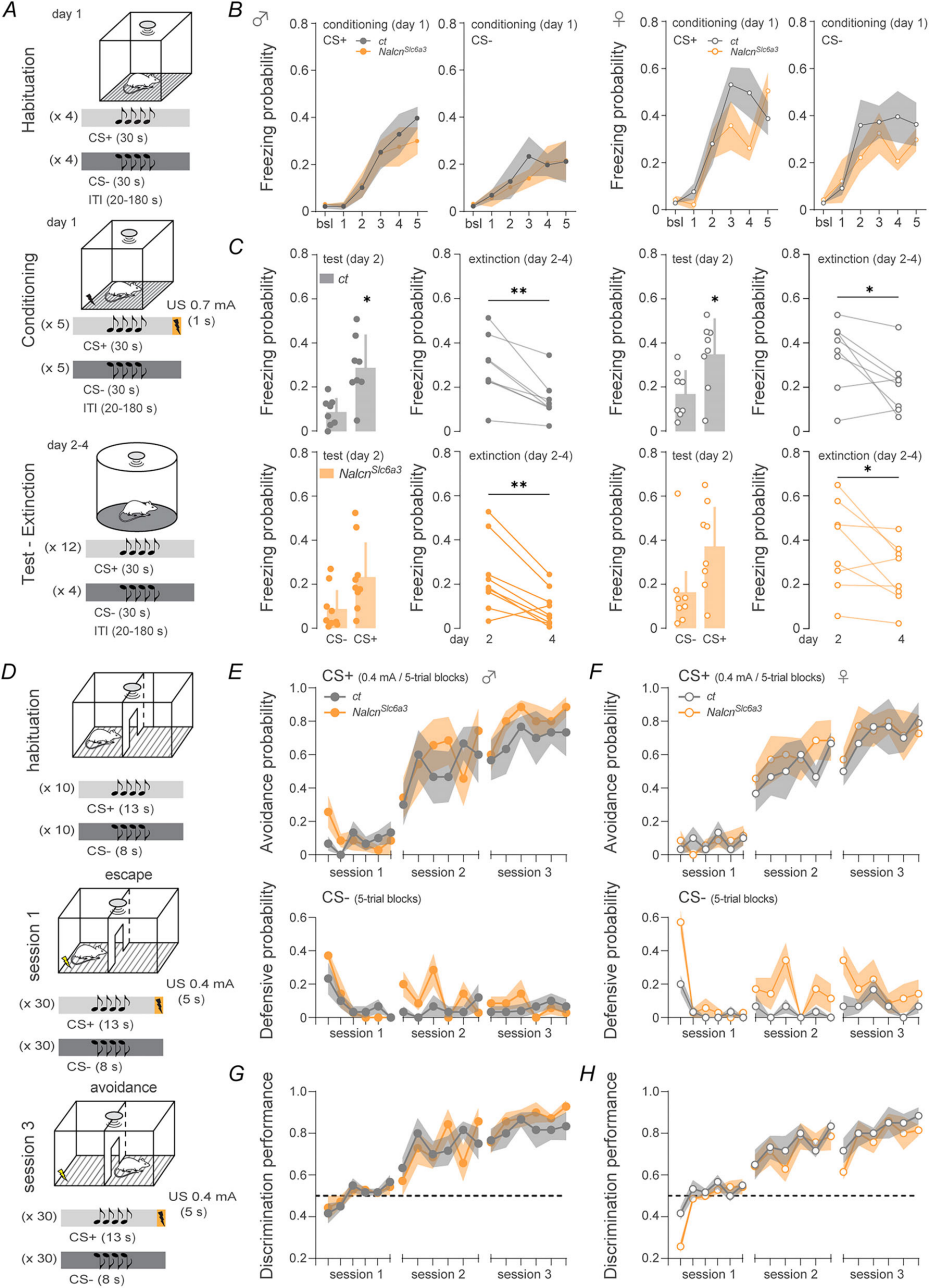
Defensive responses evoked by threat‐predicting auditory cues in mice lacking *Nalcn* in DA‐neurons *A*, schematic cartoon describing the protocol used to evaluate discriminative auditory threat conditioning. *B* and *C*, probability of CS+ and CS− to evoke freezing responses mice during the conditioning (day 1) (*B*). Two‐way RM ANOVA, CS+: *F*_(1, 15)_ = 0.1917, *P* = 0.668 ; CS−: *F*_(1, 90)_ = 0.2960, *P* = 0.588, for male, CS+: *F*_(1, 7)_ = 1.164, *P* = 0.317, CS−: *F*_(1, 7)_ = 0.7740, *P* = 0.408, for female; and during the test (day 2). Paired *t* test, *ct P* = 0.011, cKO *P* = 0.066 for male, *ct P* = 0.003, cKO *P* = 0.078. Freezing responses after extinction (day 2 to day 4) in male (left panels) *ct* (*n* = 8) *P* = 0.003 and *Nalcn^Slc6a3^* (*n* = 9) *P* = 0.004 and female (right panels) *ct* (*n* = 8) *P* = 0.033 and *Nalcn^Slc6a3^* mice (*n* = 8) *P* = 0.047. *D*, schematic cartoon describing the protocol used to evaluate discriminative active avoidance learning. *E* and *F*, avoidance probability (number of times the animal shuttled to the adjacent compartment during the CS+, upper panel), two‐way RM ANOVA, genotype *F*_(1, 11)_ = 0.4234, *P* = 0.529 for male, genotype *F*_(1, 11)_ = 0.2292, *P* = 0.642 for female mice, and defensive probability (number of times the animal shuttled to the adjacent compartment during the CS−, lower panel) among three sessions in male mice (*E*) two‐way RM ANOVA *F*_(1, 11)_ = 2.323, *P* = 0.152, and female (*F*) *ct* and *Nalcn^Slc6a3^* mice *F*_(1, 11)_ = 5.678, *P* = 0.036. *G* and *H*, discrimination performance in male (*G*), two‐way ANOVA, genotype *F*_(1, 11)_ = 0.09917, *P* = 0.759 and female (*H*) *F*_(1, 11)_ = 0.9456, *P* = 0.352, *ct* and *Nalcn^Slc6a3^* mice (female: *ct, n* = 6, *Nalcn^Slc6a3^, n* = 7; male: *ct, n* = *6, Nalcn^Slc6a3^, n* = *7*). *P < 0.05, **P < 0.01.

To complement our analysis, we assessed the reactivity to threat‐predicting cues during dynamic defensive behaviours. *Ct* and *Nalcn^Slc6a3^* mice were subjected to discriminative active avoidance learning (Fig. 6*D*). Male and female mice from both genotypes were trained using low‐intensity shocks (0.4 mA) over three consecutive days (Fig. 6*D*). As expected, during the first session, mice from both sexes and genotypes exhibited escape behaviours, characterized by low avoidance probability (Fig. 6*E*,*F*). Across subsequent sessions, *ct* and *Nalcn^Slc6a3^* mice gradually learned to avoid the threat following CS+ presentation and equally discriminated between CS+ and CS− (Fig. 6*E*–*H*). Together, these findings indicate that intact NALCN signalling in midbrain DA neurons is not necessary for defensive behaviours employed in response to threat‐predicting auditory cues.

### Reward‐related responses to sweet solution are unaltered in *Nalcn^Slc6a3^* mice

We recently found that reward‐seeking behaviours were not impacted in *Nalcn^Slc6a3^* mice during operant conditioning (Castell et al., 2026). To complement this, we sought to determine whether deletion of *Nalcn* in DA‐neurons alters reward‐related responses to sweet solution. To do so, we performed a sugar preference test using sucrose and saccharin, two caloric and non‐caloric sweeteners, respectively. When given a choice between sucrose and water, *ct* and *Nalcn^Slc6a3^* mice, both males and females, displayed a similar preference for the sucrose solution over water (Fig. 7*A*,*D*). Next, mice were exposed to a choice between saccharin and water. Although *Nalcn^Slc6a3^* male mice showed a slight, non‐significant tendency toward reduced saccharin consumption (Fig. 7*B*), no major differences were observed between all experimental groups (Fig. 7*B*,*E*). Because caloric sweeteners are more reinforcing than non‐caloric ones in terms of DA dynamics (Beeler et al., 2012; McCutcheon et al., 2012), we next exposed *ct* and *Nalcn^Slc6a3^* mice to a choice between sucrose and saccharin solutions. As shown in Fig. 7*C*,*F*, all groups, both males and females, exhibited a clear and stronger preference for sucrose over saccharin. Overall, these results indicate that deletion of NALCN in DA‐neurons does not alter the discrimination and preference of sugar‐associated natural stimuli.

**Figure 7:**
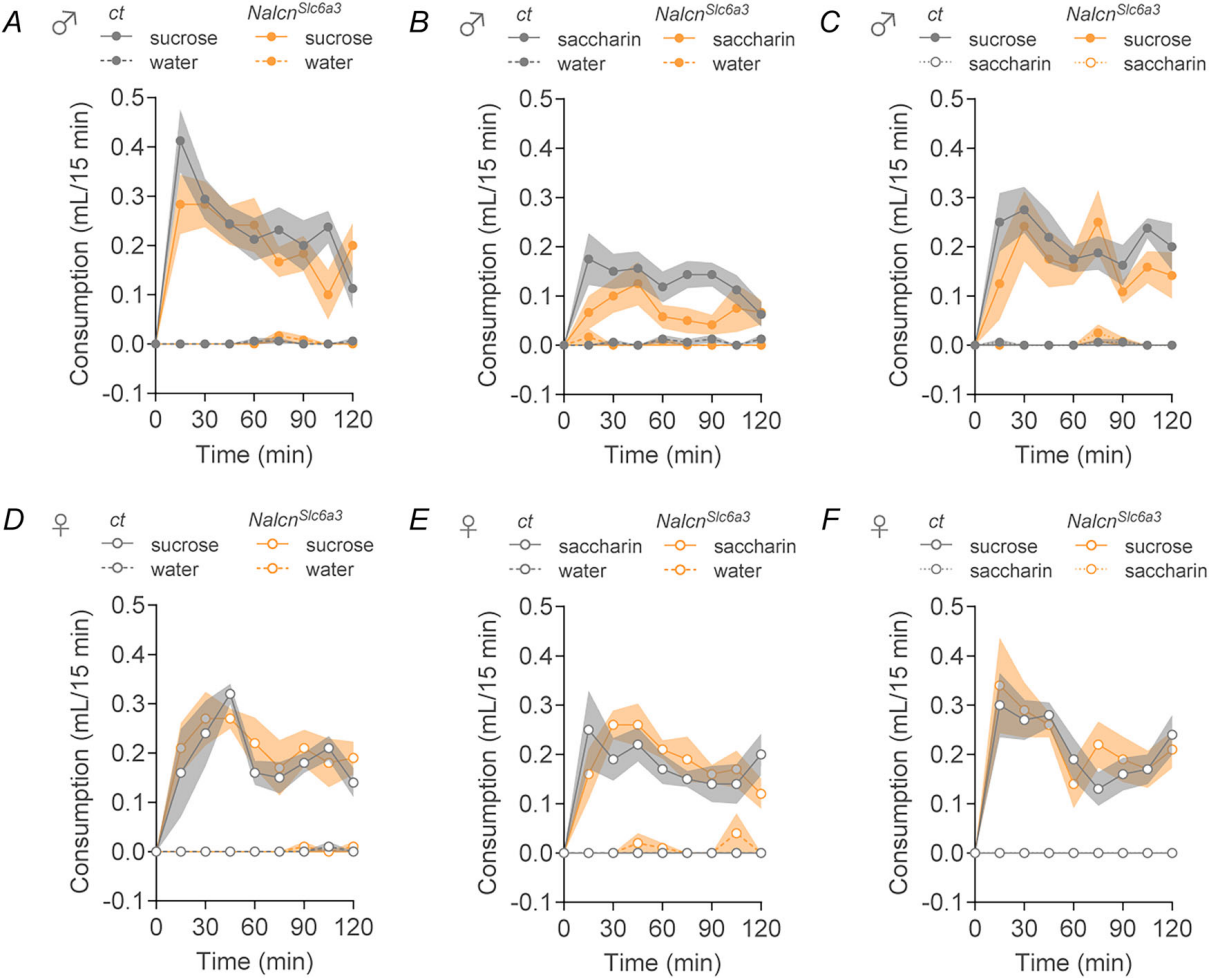
Dynamics of sweet reinforcing liquid consumption in *ct* and *Nalcn^Slc6a3^* mice Graphics depicting the time‐course of liquid intake over a 120 min period, comparing control (*ct*, grey lines/shading) and *Nalcn^Slc6a3^* mice (orange lines/shading) across different rewarding taste stimuli. Top panels (*A*–*C*) represent male mice, and bottom panels (*D*–*F*) represent female mice. *A* and *D*, sucrose *vs*. water consumption. Time‐course of 15 min interval consumption (mL/15 min) of a caloric reinforcing stimulus (sucrose; solid lines) *versus* water (dashed lines) in male (*A*) two‐way RM ANOVA, time *F*_(8, 96)_ = 12.86, *P* < 0.0001, genotype, *F*_(1, 12)_ = 0.5852, *P* = 0.459, interaction *F*_(8, 96)_ = 1.798, *P* = 0.087 and female (*D*) mice, time *F*_(8, 64)_ = 8.417, *P* = 0.0006, genotype *F*_(1, 8)_ = 0.3697, *P* = 0.560, interaction *F*_(8, 64)_ = 0.4559, *P* = 0.709. *B* and *E*, saccharin *vs*. water consumption. Time‐course of 15 min interval consumption of a non‐caloric sweet stimulus (saccharin; solid lines) *versus* water (dashed lines) in male (*B*) two‐way ANOVA, time *F*_(8, 96)_ = 4.732, *P* = 0.0031, genotype *F*_(1, 12)_ = 3.542, *P* = 0.084, interaction *F*_(8, 96)_ = 1.222, *P* = 0.294, and female (*E*), time *F*_(8, 64)_ = 7.407, *P* = 0.001, genotype *F*_(1, 8)_ = 0.1233, *P* = 0.735, interaction *F*_(8, 64)_ = 1.184, *P* = 0.337 mice. *C* and *F*) sucrose *vs*. saccharin consumption. Direct comparison of the consumption kinetics between caloric (sucrose; solid lines) and non‐caloric (saccharin; dotted lines) sweet reinforcing stimuli in male (*C*) two‐way ANOVA, time, *F*_(8, 96)_ = 6.088, *P* = 0.0008, genotype *F*_(1, 12)_ = 1.512, *P* = 0.242, interaction *F*_(8, 96)_ = 0.7951, *P* = 0.608, and female (*F*) mice, time *F*_(8, 64)_ = 9.212, *P* = 0.001, genotype *F*_(1, 8)_ = 0.1340, *P* = 0.724, interaction *F*_(8, 64)_ = 0.4658, *P* = 0.656.

### *Nalcn^Slc6a3^* male mice display enhanced locomotor responsiveness to cocaine

We next examined the ability of a single administration of cocaine (15 mg/kg, i.p.) to induce motor activity in male and female *ct* and *Nalcn^Slc6a3^* mice. Cocaine‐evoked locomotor response was enhanced in *Nalcn^Slc6a3^* male mice as compared to *ct* (Fig. 8*A*). Given that this hyperactivity relies largely on increased dopaminergic tone in the Acb, we performed *in vivo* microdialysis to assess cocaine‐induced increases in extracellular DA levels. Surprisingly, *ct* and *Nalcn^Slc6a3^* male mice displayed similar increased synaptic DA release following cocaine administration (Fig. 8*B*). In contrast, similar psychomotor responses and increased DA release were observed in *ct* and *Nalcn^Slc6a3^* female mice after a single injection of cocaine (Fig. 8*C*,*D*). Of interest, the enhanced locomotor response observed with cocaine was not recapitulated by a single administration of the selective blockade of the DA transporter achieved using GBR12783. A slight increase was observed in the first 30 min at a dose of 15 mg/kg in male but not in female *Nalcn^Slc6a3^* mice. At a higher dose (30 mg/kg), only female *Nalcn^Slc6a3^* mice maintained a stronger locomotor response (Fig. 8*E*,*F*). We therefore assessed the effect of methylphenidate, which blocks DA and noradrenaline transporters (Gatley et al., 1996). At a dose of 7.5 mg/kg (i.p.), methylphenidate increased locomotor activity similarly in both *ct* and *Nalcn^Slc6a3^* mice irrespective of sex, while at 10 mg/kg the psychomotor effects tended to be more pronounced in both male and female *Nalcn^Slc6a3^* mice as compared to controls (Fig. 8*G*,*H*). Together our results reveal that the loss of NALCN in DA‐neurons differentially affects psychomotor responses induced by psychostimulants and suggest that the hyperresponsiveness to cocaine observed in *Nalcn^Slc6a3^* male mice is not a result of enhanced DA levels.

**Figure 8:**
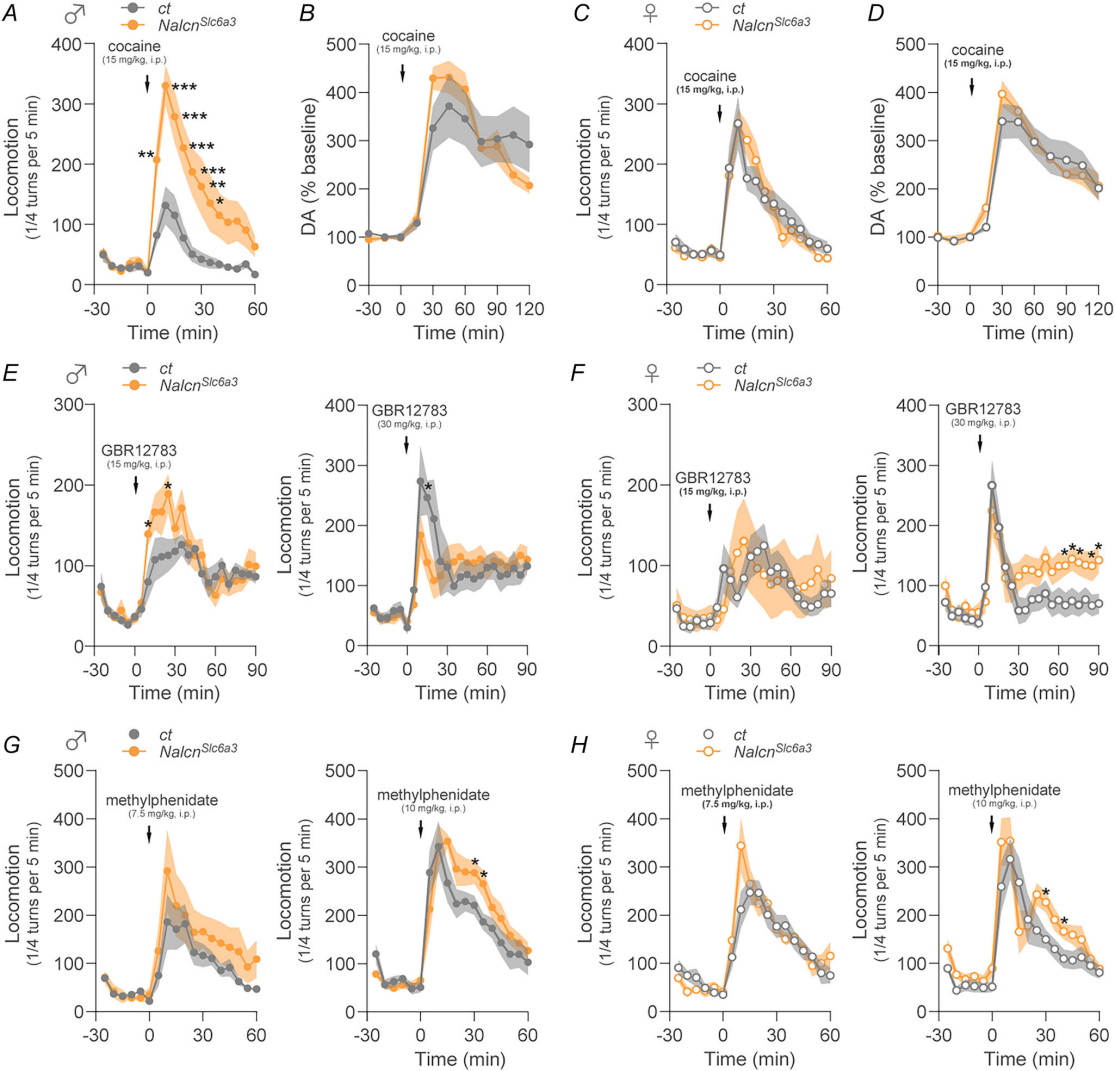
Cocaine‐induced psychomotor effects and DA release in *ct* and *Nalcn^Slc6a3^* mice *A*, horizontal locomotor activity after a single administration of cocaine (15 mg/kg, i.p.) in male mice (*ct, n* = 11; *Nalcn^Slc6a3^*, *n* = 9), two‐way ANOVA, time *F*_(17, 304)_ = 32.66, *P* < 0.0001, genotype *F*_(1, 18)_ = 10.02, *P* = 0.005 interaction, *F*_(17, 304)_ = 9.033, *P* < 0.0001. *B*, accumbal extracellular level of DA after a single cocaine administration (15 mg/kg, i.p.) in male mice (*ct, n* = 5; *Nalcn^Slc6a3^*, *n* = 4). Two way ANOVA, time *F*_(11, 77)_ = 36.22, *P* < 0.0001, genotype *F*_(1, 7)_ = 0.006641, *P* = 0.937, interaction *F*_(11, 77)_ = 1.865, *P* = 0.057. *C* and *D*, similar to *A* and *B* but in female mice (*C*) (*ct, n* = 14; *Nalcn^Slc6a3^*, *n* = 10). Two way ANOVA, time *F*_(17, 374)_ = 35.56, *P* < 0.0001, genotype *F*_(1, 22)_ = 0.01958, *P* = 0.890, interaction *F*_(17, 374)_ = 1.003, *P* = 0.454, and (*D*) (*ct*, *n* = 6, *Nalcn^Slc6a3^ n* = 3) time *F*_(11, 77)_ = 40.40, *P* < 0.0001, genotype *F*_(1, 7)_ = 0,0 5451, *P* = 0.822, interaction *F*_(11, 77)_ = 0.5715, *P* = 0.846. *E* and *F*, horizontal locomotor activity after an acute injection of GBR12783 (15 or 30 mg/kg, i.p.) in male mice (15 mg/kg: *ct, n* = 8; *Nalcn^Slc6a3^*, *n* = 8; 30 mg/kg: *ct, n* = 8; *Nalcn^Slc6a3^*, *n* = 12). Two‐way ANOVA, 15 mg/kg: time *F*_(23, 322)_ = 19.82, *P* < 0.0001, genotype *F*_(1, 14)_ = 0,8593, *P* = 0.370, interaction *F*_(23, 322)_ = 2.563, *P* = 0.0001; 30 mg/kg: time *F*_(23, 414)_ = 9.595, *P* < 0.0001, genotype *F*_(1, 18)_ = 0.08318, *P* = 0.776, interaction *F*_(23, 414)_ = 1.745, *P* = 0.151, and female mice (15 mg/kg: *ct, n* = 10; *Nalcn^Slc6a3^*, *n* = 7; 30 mg/kg: *ct, n* = 11; *Nalcn^Slc6a3^*, *n* = 10), 15 mg: time *F*_(17, 204)_ = 13.54, *P* < 0.0001, genotype *F*_(1, 12)_ = 1,288, *P* = 0.2786, interaction *F*_(17, 204)_ = 0.8244, *P* = 0.664 ; 30 mg/kg: time *F*_(23, 437)_ = 10.24, *P* < 0.0001, genotype *F*_(1, 19)_ = 3.310, *P* = 0.085, interaction *F*_(23, 437)_ = 1.716, *P* = 0.154. *G* and *H*, horizontal locomotor activity after an acute injection of methylphenidate (7.5 or 10 mg/kg, i.p.) in male mice (7.5 mg/kg: *ct, n* = 8; *Nalcn^Slc6a3^*, *n* = 6; 10 mg/kg: *ct, n* = 8; *Nalcn^Slc6a3^*, *n* = 10). Two‐way ANOVA, 7.5 mg/kg: time *F*_(17, 204)_ = 13.54, *P* < 0.0001, genotype *F*_(1, 12)_ = 1.288, *P* = 0.279, interaction *F*_(17, 204)_ = 0.8244, *P* = 0.664; 10 mg/kg: time *F*_(17, 272)_ = 37.73, *P* < 0.0001, genotype *F*_(1, 16)_ = 1.209, *P* = 0.288, interaction *F*_(17, 272)_ = 1.933, *P* = 0.116; and female mice (7.5 mg/kg: *ct, n* = 7; *Nalcn^Slc6a3^*, *n* = 7; 10 mg/kg: *ct, n* = 8; *Nalcn^Slc6a3^*, *n* = 8), 7.5 mg/kg: time *F*_(17, 204)_ = 32.28, *P* < 0.0001, genotype *F*_(1, 12)_ = 0,2359, *P* = 0.636, interaction *F*_(17, 204)_ = 2.032, *P* = 0.0111 ; 10 mg/kg: time *F*_(17, 238)_ = 26.51, *P* < 0.0001, genotype *F*_(1, 14)_ = 1.918, *P* = 0.188, interaction *F*_(17, 238)_ = 1.798, *P* = 0.151. *P < 0.05, **P < 0.01, ***P < 0.0001.

We therefore hypothesized that hypersensitivity of D1R‐mediated signalling could account for this enhanced responsiveness to cocaine. We first examined by immunofluorescence and western blotting the expression levels of striatal proteins identified as important regulators of psychomotor responses induced by cocaine. We found no alteration in the levels of D1R (Fig. 9*A*,*B*), glutamate receptor GluA1 subunit (Fig. 9*B*), dopamine‐ and cAMP‐regulated phosphoprotein, *M*_r_ = 32,000 (DARPP‐32) and G_αolf_ in the striatum of *Nalcn^Slc6a3^* mice as compared to *ct* (Fig. 9*C*,*D*). In addition, the acute locomotor response elicited by the D1R‐like agonist SKF81297 (5 mg/kg, i.p.) was similar between control and *Nalcn^Slc6a3^* mice from both sexes (Fig. 9*E*), suggesting that enhanced response to cocaine observed in *Nalcn^Slc6a3^* male mice do not result from altered D1R‐mediated responsiveness.

**Figure 9:**
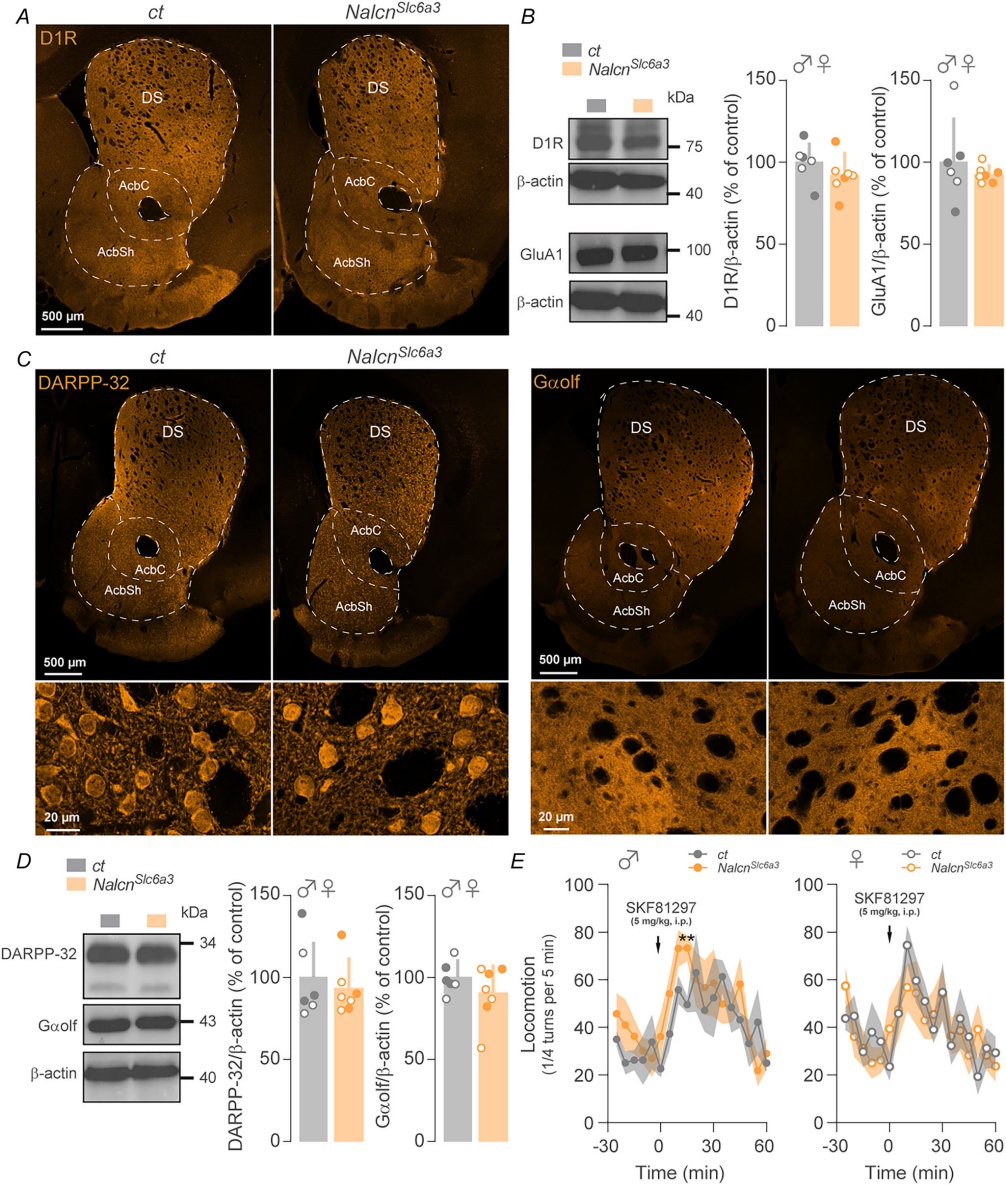
D1R downstream targets in control and *Nalcn^Slc6a3^* mice *A*, immunofluorescence against D1R on striatum sections. *B*, western blot analysis and histograms quantifying D1R and GluA1 on homogenates of striata extract from *ct* (female, *n* = 3; male, *n* = 3) and *Nalcn^Slc6a3^* mice (female, *n* = 4; male, *n* = 3). Unpaired *t* test, *P* = 0.239 and *P* = 0.415 respectively. *C*, immunofluorescence against DARPP‐32 (left panel) and Gαolf (right panel) on striatum sections. *D*, western blot analysis and histograms quantifying DARPP‐32 and Gαolf on homogenates of striata extract from *ct* (female, *n* = 3; male, *n* = 3) and *Nalcn^Slc6a3^* mice (female, *n* = 4; male, *n* = 3). Unpaired *t* test, *P* = 0.591 and *P* = 0,248 respectively. *E*, locomotor activity after an acute injection of SKF811297 (5 mg/kg, i.p.) (female: *ct, n* = 8, *Nalcn^Slc6a3^, n* = 7; male: *ct, n* = 7*, Nalcn^Slc6a3^, n* = 8). Two‐way RM ANOVA, time *F*_(17, 221)_ = 5.944, *P* = 0.0003, genotype *F*_(1, 13)_ = 0.1335, *P* = 0.721, interaction *F*_(13, 221)_ = 7.932, *P* < 0.0001 for male; time *F*_(17, 221)_ = 5.834, *P* < 0.0001, genotype *F*_(1, 13)_ = 0.01665, *P* = 0.899, interaction *F*_(17, 221)_ = 1.133, *P* = 0.352 for female. Abbreviations: AcbC, nucleus accumbens core; AcbSh, nucleus accumbens shell; DS, dorsal striatum. ***P < 0.0001.

### *Nalcn^Slc6a3^* mice display enhanced psychomotor sensitization to d‐amphetamine

To determine whether this effect was specific to cocaine, in a separate cohort of mice we tested the impact of d‐amphetamine on motor activity and synaptic DA release. In male mice from both genotypes, the first administration of d‐amphetamine at a dose of 1.5 mg/kg did not significantly change the amplitude of locomotor response despite the elevation of extracellular DA levels (Fig. 10*A*,*B*). Upon repeated exposure, both genotypes developed psychomotor sensitization, which was more pronounced in *Nalcn^Slc6a3^* male mice (Fig. 10*C*,*D*). In female mice, d‐amphetamine‐evoked motor activity was slightly enhanced in *Nalcn^Slc6a3^* mice (Fig. 10*E*), an effect most probably explained by the enhanced synaptic DA release observed in the Acb of *Nalcn^Slc6a3^* female mice (Fig. 10*F*). Similar to male mice, repeated exposure to d‐amphetamine resulted in a progressive and long‐lasting enhancement of locomotor responses, which was more pronounced in *Nalcn^Slc6a3^* female mice than *ct* (Fig. 10*G*,*H*). Together our results indicate that deletion of *Nalcn* in midbrain DA‐neurons leads to enhanced locomotor responsiveness to psychostimulant drugs.

**Figure 10:**
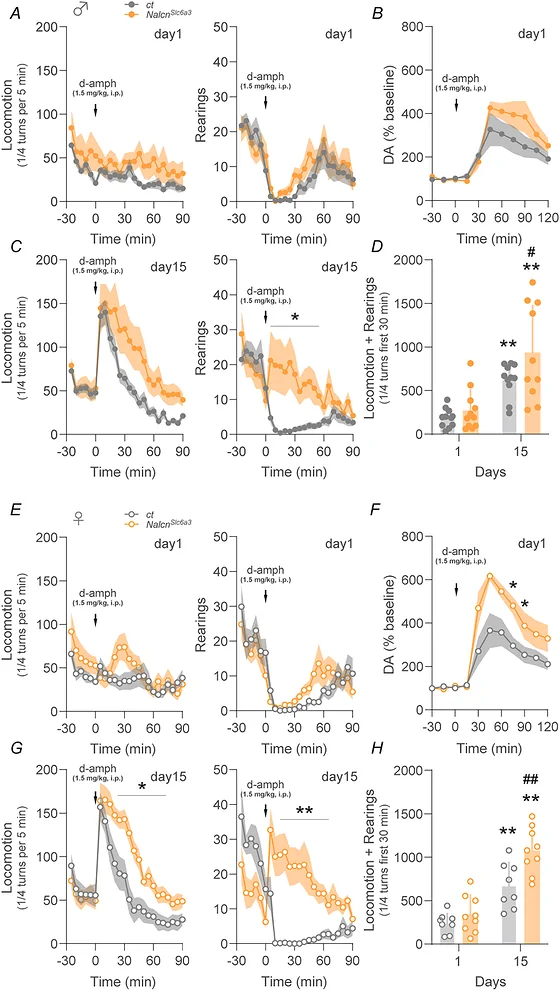
DA release and psychomotor sensitization induced by d‐amphetamine in *ct* and *Nalcn^Slc6a3^* mice *A*, locomotion and rearing after an acute injection of d‐amphetamine (1.5 mg/kg, i.p.) (day 1) in male mice (*ct, n* = 11; *Nalcn^Slc6a3^*, *n* = 11). Two‐way RM ANOVA, locomotion: time *F*_(23, 460)_ = 6.284, *P* = 0.0001, genotype *F*_(1, 20)_ = 1.560, *P* = 0.226, interaction *F*_(23, 460)_ = 0.9528, *P* = 0.527; rearing: time *F*_(23, 460)_ = 14.41, *P* < 0.0001, genotype *F*_(1, 20)_ = 0.4361, *P* = 0.517 interaction *F*_(23, 460)_ = 0.8896, *P* = 0.613. *B*, accumbal extracellular level of DA after an acute injection of d‐amphetamine in male mice (*ct, n* = 4; *Nalcn^Slc6a3^*, *n* = 4). Two‐way RM ANOVA, time *F*_(11, 66)_ = 28.70, *P* < 0.0001, genotype *F*_(1, 6)_ = 1.620, *P* = 0.250, interaction *F*_(11, 66)_ = 2.005, *P* = 0.042. *C*, locomotion and rearing in d‐amphetamine‐treated *ct* (*n* = 11) and *Nalcn^Slc6a3^* (*n* = 10) male mice for five consecutive days and challenged with d‐amphetamine after a 10 day withdrawal period. Two‐way RM ANOVA, locomotion: time *F*_(23, 431)_ = 33.54, *P* < 0.0001, genotype *F*_(1, 19)_ = 3.043, *P* = 0.097, interaction *F*_(23, 431)_ = 3.041, *P* < 0.0001; rearing: time *F*_(23, 431)_ = 10.80, *P* < 0.0001, genotype *F*_(1, 19)_ = 4.460, *P* = 0.048, interaction *F*_(23, 431)_ = 4.524, *P* < 0.0001 *D*, cumulative locomotor activity (locomotion and rearings) over 30 min in day 1 and day 15 in male mice (*ct, n* = 11; *Nalcn^Slc6a3^*, *n* = 10). Two‐way RM ANOVA, day *F*_(1, 19)_ = 63.35, *P* < 0.0001, genotype *F*_(1, 19)_ = 2.807, *P* = 0.110, interaction *F*_(1, 19)_ = 2.518, *P* = 0.129. *E*, similar to *A* in female mice (*ct, n* = 8; *Nalcn^Slc6a3^*, *n* = 9). Two‐way RM ANOVA, locomotion: time *F*_(23, 345)_ = 4.766, *P* = 0.0057, genotype *F*_(1, 15)_ = 1.179, *P* = 0.295, interaction *F*_(23, 345)_ = 1.675, *P* = 0.028 ; rearing: time *F*_(23, 345)_ = 14.56, *P* < 0.0001, genotype *F*_(1, 15)_ = 0.2405, *P* = 0.631, interaction *F*_(23, 345)_ = 1.367, *P* = 0.123. *F*, similar to *B* in female mice (*ct, n* = 4; *Nalcn^Slc6a3^*, *n* = 5). Two‐way RM ANOVA, time *F*_(7, 77)_ = 36.24, *P* < 0.0001, genotype *F*_(1, 7)_ = 8.115, *P* = 0.025, interaction *F*_(7, 77)_ = 5.716, *P* < 0.0001. *G* and *H*, similar to *C* and *D* in female mice (*ct, n* = 8; *Nalcn^Slc6a3^*, *n* = 9). Two way RM ANOVA, time *F*_(23, 321)_ = 30.85, *P* < 0.0001, genotype *F*_(1,15)_ = 7.303, *P* = 0.016, interaction *F*_(23, 321)_ = 3.695, *P* < 0.0001; rearing: time *F*_(23, 321)_ = 7.944, *P* < 0.0001, genotype *F*_(1, 15)_ = 8.965, *P* = 0.009, interaction *F*_(23, 321)_ = 8.868, *P* < 0.0001; day: *F*_(1, 15)_ = 66.93, *P* < 0.0001, genotype *F*_(1, 15)_ = 9.275, *P* = 0.008, interaction *F*_(1, 15)_ = 4.560, *P* = 0.0496. *P < 0.05, **P < 0.01, ***P < 0.0001, ^#^ *P* < 0.05.

## Discussion

The present study identifies the sodium leak channel NALCN as a key contributor to the pacemaker activity of VTA DA‐neurons. Importantly, reduced spontaneous firing in *Nalcn^Slc6a3^* mice does not translate into altered basal levels of extracellular accumbal DA or spontaneous locomotor activity. In addition, we found that behavioural responses driven by negative or positive reinforcement are also preserved in *Nalcn^Slc6a3^* mice. In contrast, *Nalcn^Slc6a3^* mice exhibit impaired D2R‐mediated signalling at DA terminals and sex‐biased altered responsiveness to psychostimulant exposure.

Pacemaker activity is one of the key electrophysiological features of midbrain DA‐neurons (Roeper, 2013). Over the past decades accumulating evidence indicates that distinct ionic conductances sustain the different mechanisms underlying pacemaking of DA‐neurons originating from SNc and VTA, respectively (Gantz et al., 2018; Khaliq & Bean, 2010; Liss & Roeper, 2008). Thus, SNc pacemaking relies primarily on currents mediated by voltage‐gated calcium channels (Gantz et al., 2018), while in VTA DA‐neurons it depends mainly on two sodium‐permeable conductances (Khaliq & Bean, 2010). Our *in vivo* and *ex vivo* results extend previous observations (Cobb‐Lewis et al., 2023; Wang et al., 2024), revealing a role of the non‐selective sodium leak channel NALCN in regulating the pacemaker activity of VTA DA‐neurons. However, contrasting results have been obtained in the SNc depending on the approaches used to assess the role of this channel. In fact, while genetic approaches using mice lacking NALCN in DA‐neurons report that spontaneous activity of SNc DA‐neurons is profoundly altered (Philippart & Khaliq, 2018; Um et al., 2021), pacemaker firing is still present following acute pharmacological inhibition of NALCN (Cobb‐Lewis et al., 2023; Jehasse et al., 2021). Despite these subtle differences, recent evidence indicates that NALCN acts synergistically with other leak channels to sustain the pacemaking activity of both SNc and VTA DA‐neurons. Indeed, the transient receptor potential cation channel subfamily C member 3 (TRPC3) is strongly involved in the pacemaker activity of SNc DA‐neurons (Hahn et al., 2023; Um et al., 2021), while TRPC6 appears to be involved in VTA DA‐neurons (Wang et al., 2024). Given the electrophysiological heterogeneity among DA‐neurons, future studies will be necessary to refine the contribution of NALCN channels within these molecularly and functionally distinct subpopulations.

Although challenging, activity of VTA DA‐neurons can be reliably isolated via extracellular single‐unit recordings in anaesthetized mice (PMID: 2881804). In the present study, classical cell identification parameters normally used to capture their activity were barely detectable in *Nalcn^Slc6a3^* mice, thus precluding an in‐depth *in vivo* characterization of their firing properties. Despite this apparent ‘silent activity’, several lines of evidence indicate that DA‐neurons lacking NALCN are active. First, salient aversive stimuli trigger phasic increases in the firing rate of VTA DA‐neurons, suggesting they are not ‘silent’ and that responses can be evoked. Second, basal extracellular Acb DA levels are not reduced in *Nalcn^Slc6a3^* mice, indicating that even if basal firing properties are altered, DA availability is unchanged. Finally, our fibre photometry experiments indicate that the dynamics of DA transients can be measured, suggesting that the impact of anaesthesia itself on the activity of VTA DA‐neurons lacking NALCN could be one of the major confounding factors. Indeed, under anaesthetized conditions, the tonic firing of DA‐neurons relies predominantly on intrinsic pacemaker mechanisms. In awake animals, however, ongoing network activity and synaptic inputs can sustain DA transmission despite profound alterations in intrinsic excitability.

*In vitro* and *in vivo* evidence indicates that G protein‐coupled receptors can modulate NALCN conductances (Cochet‐Bissuel et al., 2014). Thus, the neuropeptide substance P activates NALCN in VTA DA‐neurons through a neurokinin 1 receptor‐dependent mechanism (Lu et al., 2009). Conversely, activation of D2R negatively regulates the activity of NALCN (Philippart & Khaliq, 2018; Topalidou et al., 2017), a mechanism that contributes to the D2R‐mediated somatodendritic inhibition onto VTA DA‐neurons (Philippart & Khaliq, 2018). Using complementary approaches, we provide evidence that D2R‐mediated regulation of the DA levels and TH phosphorylation in the Acb are strongly impacted in *Nalcn^Slc6a3^* mice. These effects occur independently of altered striatal expression levels of D2R, TH and VMAT2, suggesting that the loss of NALCN may also affect the functions of autoD2R at the presynaptic site of midbrain DA‐neurons through a mechanism not yet established. Interestingly, a recent study revealed that SNARE proteins inhibit NALCN activity, therefore regulating electrical signalling (Usher et al., 2025). Future work will be necessary to determine whether similar regulatory mechanisms occur in the DA terminals, thus sustaining a role of the NALCN channel in the regulation of DA turnover, synthesis and release. Our study also provides evidence that altered presynaptic D2R‐mediated functions translate at a more integrated level. In fact, similarly to mice lacking autoD2R (Anzalone et al., 2012; Bello et al., 2011), the hypolocomotion induced by a low dose of quinpirole was drastically reduced in *Nalcn^Slc6a3^* mice. Although autoD2Rs are functional in both male and female mice, our results suggest that the use of this inhibitory feedback mechanism might be more prominent in male than in female mice. Indeed, our analysis shows that male mice display a lower sensitivity to the inhibitory effects of quinpirole as compared to females, an effect that could be attributed to the level of circulating sex steroid hormones (e.g. oestrogen) known to potently regulate the excitability and somatodendritic inhibition of DA‐neurons (Gordon & Fields, 1989; Thompson & Moss, 1994; Vandegrift et al., 2017).

Increasing evidence suggests that midbrain DA transmission plays a critical role in the processing of threat‐related events (Duvarci, 2024). Notably, circuit‐based manipulations have highlighted that distinct DA‐projecting neurons play complementary roles in associative learning enabling environmental and sensory cues to predict threating situations. Thus, DA‐neurons projecting to the tail of striatum are necessary to form such associative learning (Zafiri et al., 2025). Conversely, DA‐neurons innervating the Acb drive extinction (Luo et al., 2018; Salinas‐Hernández et al., 2023), while those projecting to the central amygdala are critical for fear discrimination (Jo et al., 2018). To date, converging evidence indicates that phasic midbrain DA‐neuron activity evoked by threat‐predictive stimuli (Gore et al., 2014) underlines the contribution of these distinct DA circuits in associative aversive learning. Thus, mice lacking the NR1 subunit of the NMDAR in midbrain DA‐neurons, which are unable to engage phasic increases in DA‐neuron firing, display strong deficits in threat processing (Jones et al., 2015; Zweifel et al., 2009, 2011). In contrast, our study provides evidence that discriminative associative learning of auditory cues predicting aversive outcomes is not impaired in *Nalcn^Slc6a3^* mice, suggesting that the pacemaker activity of midbrain DA‐neurons is not mandatory to sustain threat processing.

Spontaneous locomotor activity is under the tonic control of DA tone. Hence, mice lacking DAT display strong hyperlocomotion directly linked to the heightened levels of DA in the extracellular space (Giros et al., 1996). Similarly, disruption of autoD2R signalling results in enhanced novelty‐induced locomotion (Anzalone et al., 2012; Bello et al., 2011). Here, we show that selective loss of *Nalcn* in DA‐neurons does not affect spontaneous locomotion and/or exploratory drive, suggesting that the pacemaker activity and excitability of DA‐neurons do not play a prominent role in the control of spontaneous motor responses. On the other hand, our results indicate that reduced NALCN conductance in DA‐neurons alters sensitivity to psychostimulant drugs. *Nalcn^Slc6a3^* male mice, but not females, display increased behavioural sensitivity to cocaine that occurs independently of striatal D1R hypersensitivity. Notably, this enhanced psychomotor response is dissociated from DA signalling, as cocaine‐induced DA release remained comparable between *Nalcn^Slc6a3^* and control mice. Although relatively uncommon, this phenotype resembles that reported in mouse models lacking G protein‐coupled receptor kinase 6 (Gainetdinov et al., 2003), Homer1 and Homer2 (Szumlinski et al., 2004), or the norepinephrine transporter (PMID: 10769386). Conversely, reduced cocaine‐induced hyperlocomotion has also been observed without alterations in accumbal DA levels, such as in mice with decreased expression of the NR1 subunit of the NMDAR (Ramsey et al., 2008) or lacking metabotropic glutamate receptor 5 (Chiamulera et al., 2001). Together, these findings support the idea that dissociation between acute psychomotor responses and dopamine release can occur under specific conditions.

Although less pronounced, the behavioural phenotype of *Nalcn^Slc6a3^* mice is also reminiscent of the one previously described in mice displaying altered feedback inhibition onto DA‐neurons. Specifically, genetic or viral‐mediated disruption of autoD2R signalling (Anzalone et al., 2012; Bello et al., 2011; de Jong et al., 2015) or GIRK2 channel activity (Arora et al., 2010; McCall et al., 2017, 2019) leads to an enhanced psychomotor effect of cocaine. However, in contrast to our findings, both males and females are hypersensitive to cocaine through a mechanism probably driven by elevated extracellular DA release in the Acb consequently to the altered presynaptic D2R‐mediated signalling (Arora et al., 2010; Bello et al., 2011). Our work also reveals that enhanced behavioural sensitivity observed in male *Nalcn^Slc6a3^* mice is not generalizable to all classes of psychostimulants since the psychomotor effects induced by methylphenidate or GBR12783 are comparable between both genotypes. On the other hand, only female *Nalcn^Slc6a3^* mice exhibit a modest increase in locomotion following low‐dose d‐amphetamine, which correlates with the enhanced d‐amphetamine‐induced DA release. These findings further extend previous observations highlighting divergent psychostimulant responses observed in mice with disrupted DA transmission. For example, mice expressing half of VMAT2 in DA‐neurons show enhanced psychomotor responses to d‐amphetamine but not cocaine (Isingrini et al., 2016), as also do mice lacking the calcium sensor synaptotagmin‐1 in DA‐neurons (Delignat‐Lavaud et al., 2023). Conversely, mice deficient in all neurexins in DA‐neurons exhibit reduced d‐amphetamine‐induced hyperlocomotion, while cocaine responses remain unaffected (Ducrot et al., 2023). Despite these differences in the acute response to d‐amphetamine, both male and female *Nalcn^Slc6a3^* mice show a facilitated behavioural sensitization akin to that observed in GIRK2‐deficient DA‐neuron models upon cocaine exposure (McCall et al., 2017), indicating that repeated exposure is required to reveal d‐amphetamine supersensitivity. Indeed, further research is needed to determine whether these effects result primarily from disrupted autoD2R‐mediated signalling at both dopaminergic terminals and somatodendritic compartments, and whether altered oestrogen regulation of DA‐neuron excitability contributes to these changes.

In conclusion, the present work reveals the importance of NALCN conductance in regulating the activity of VTA DA‐neurons and presynaptic D2R‐mediated signalling. This study also emphasizes that pacemaker activity is dispensable for sustaining defensive responses evoked by threat‐predictive cues as well as motivation for natural reward while it scales the responsiveness to psychostimulant drugs in a sex‐dependent manner.

## Additional information

## Competing interests

The authors declare no competing interests.

## Author contributions

A.R., F.B., F.M. and E.V. conceived the study and designed the experiments. A.R. performed behavioural experiments. A.C. and J.M.R. performed *in vivo* microdialysis experiments and analysis. C.N., N.M. and G.G. performed and analysed western blot experiments. T.L.B., F.M. and P.F. designed, performed and analysed *in vivo* juxtacellular recordings and *ex vivo* patch‐clamp recordings. A.G. and F.M. performed and analysed *in vivo* fibre photometry experiments. C.N. performed the fluorescence *in situ* hybridization. C.N. and L.C. performed immunofluorescence experiments. L.C. set up protocols to assess defensive behaviours. C.B. and A.M. provided *Nalcn^Slc6a3^* transgenic mice and performed genotyping. A.R., F.B., G.G., F.M. and E.V. wrote the manuscript with inputs from all authors.

## Funding

This work was supported by CNRS, INSERM and Fondation pour la Recherche Médicale. Emmanuel Valjent was supported by Fondation pour la Recherche Médicale (EQU202203014705) and the French National Research Agency (ANR‐20‐CE14‐0020 and ANR‐21‐CE16‐0028). Fabio Marti was supported by the French National Research Agency (ANR‐19‐CE16‐0001). Arnaud Monteil was supported by the French National Research Agency (ANR‐21‐NEU2‐0004‐01) and the Labex ‘Ion Channel Science and Therapeutics’ (ANR‐11‐LABX‐0015). Giuseppe Gangarossa was supported by the Agence Nationale de la Recherche (ANR‐21‐CE14‐0021‐01 and ANR‐23‐CE14‐0014‐02), Fédération pour la Recherche sur le Cerveau and Association France Parkinson, Institut Universitaire de France and Université Paris Cité (Stratex_Idex‐2023‐028 EMERGENCE).

## Author's present address

Laia Castell: Department of Neuroscience, Northwestern University Feinberg School of Medicine, Chicago, IL 60611, USA.

## Generative AI statement

Generative AI was not used.

## Supporting information




Peer Review History

Supporting Information

## Data Availability

Original contributions for this study are included within the article.

## References

[tjp70833-bib-0001] Anzalone, A. , Lizardi‐Ortiz, J. E. , Ramos, M. , De Mei, C. , Hopf, F. W. , Iaccarino, C. , Halbout, B. , Jacobsen, J. , Kinoshita, C. , Welter, M. , Caron, M. G. , Bonci, A. , Sulzer, D. , & Borrelli, E. (2012). Dual control of dopamine synthesis and release by presynaptic and postsynaptic dopamine D2 receptors. Journal of Neuroscience, 32(26), 9023–9034. 10.1523/JNEUROSCI.0918-12.2012 PMC3752062 22745501

[tjp70833-bib-0002] Arora, D. , Haluk, D. M. , Kourrich, S. , Pravetoni, M. , Fernández‐Alacid, L. , Nicolau, J. C. , Luján, R. , & Wickman, K. (2010). Altered neurotransmission in the mesolimbic reward system of Girk mice. Journal of Neurochemistry, 114(5), 1487–1497. 10.1111/j.1471-4159.2010.06864.x PMC2941778 20557431

[tjp70833-bib-0003] Beeler, J. A. , McCutcheon, J. E. , Cao, Z. F. H. , Murakami, M. , Alexander, E. , Roitman, M. F. , & Zhuang, X. (2012). Taste uncoupled from nutrition fails to sustain the reinforcing properties of food. European Journal of Neuroscience, 36(4), 2533–2546. 10.1111/j.1460-9568.2012.08167.x PMC3424366 22712685

[tjp70833-bib-0004] Bello, E. P. , Mateo, Y. , Gelman, D. M. , Noaín, D. , Shin, J. H. , Low, M. J. , Alvarez, V. A. , Lovinger, D. M. , & Rubinstein, M. (2011). Cocaine supersensitivity and enhanced motivation for reward in mice lacking dopamine D2 autoreceptors. Nature Neuroscience, 14(8), 1033–1038. 10.1038/nn.2862 PMC3175737 21743470

[tjp70833-bib-0005] Bertran‐Gonzalez, J. , Håkansson, K. , Borgkvist, A. , Irinopoulou, T. , Brami‐Cherrier, K. , Usiello, A. , Greengard, P. , Hervé, D. , Girault, J.‐A. , Valjent, E. , & Fisone, G. (2009). Histone H3 phosphorylation is under the opposite tonic control of dopamine D2 and adenosine A2A receptors in striatopallidal neurons. Neuropsychopharmacology, 34(7), 1710–1720. 10.1038/npp.2008.228 PMC2758561 19158668

[tjp70833-bib-0006] Bromberg‐Martin, E. S. , Matsumoto, M. , & Hikosaka, O. (2010). Dopamine in motivational control: Rewarding, aversive, and alerting. Neuron, 68(5), 815–834. 10.1016/j.neuron.2010.11.022 PMC3032992 21144997

[tjp70833-bib-0007] Castell, L. , Le Gall, V. , Cutando, L. , Petit, C. P. , Puighermanal, E. , Makrini‐Maleville, L. , Kim, H.‐R. , Jercog, D. , Tarot, P. , Tassou, A. , Harrus, A.‐G. , Rubinstein, M. , Nouvian, R. , Rivat, C. , Besnard, A. , Trifilieff, P. , Gangarossa, G. , Janak, P. H. , Herry, C. , & Valjent, E. (2024). Dopamine D2 receptors in WFS1‐neurons regulate food‐seeking and avoidance behaviours. Progress in Neuro‐Psychopharmacology & Biological Psychiatry, 129, 110883. 10.1016/j.pnpbp.2023.110883 37858736

[tjp70833-bib-0008] Castell, L. , Naon, C. , Rogliardo, A. , Ducrocq, F. , Makrini, L. , Typou, A. , Avrillon, M. , Mignon, A. , Bernat, C. , Lory, P. , Bertaso, F. , Monteil, A. , Bosch‐Bouju, C. , & Valjent, E. (2026). The sodium leak channel NALCN in Drd2+ striatal neurons regulates neuronal excitability, locomotion and food‐seeking in a sex‐dependent manner. Neuropsychopharmacol., 51(7), 1226–1236. 10.1038/s41386-026-02363-9 PMC13213062 41673358

[tjp70833-bib-0009] Cathala, A. , Lucas, G. , López‐Terrones, E. , Revest, J.‐M. , Artigas, F. , & Spampinato, U. (2022). Differential expression of serotonin2B receptors in GABAergic and serotoninergic neurons of the rat and mouse dorsal raphe nucleus. Molecular and Cellular Neuroscience, 121, 103750. 10.1016/j.mcn.2022.103750 35697176

[tjp70833-bib-0010] Chiamulera, C. , Epping‐Jordan, M. P. , Zocchi, A. , Marcon, C. , Cottiny, C. , Tacconi, S. , Corsi, M. , Orzi, F. , & Conquet, F. (2001). Reinforcing and locomotor stimulant effects of cocaine are absent in mGluR5 null mutant mice. Nature Neuroscience, 4(9), 873–874. 10.1038/nn0901-873 11528416

[tjp70833-bib-0011] Cobb‐Lewis, D. E. , Sansalone, L. , & Khaliq, Z. M. (2023). Contributions of the sodium leak channel NALCN to pacemaking of medial ventral tegmental area and Substantia Nigra dopaminergic neurons. Journal of Neuroscience, 43(41), 6841–6853. 10.1523/JNEUROSCI.0930-22.2023 PMC10573758 37640554

[tjp70833-bib-0012] Cochet‐Bissuel, M. , Lory, P. , & Monteil, A. (2014). The sodium leak channel, NALCN, in health and disease. Frontiers in Cellular Neuroscience, 8, 132. 10.3389/fncel.2014.00132 PMC4033012 24904279

[tjp70833-bib-0013] Cutando, L. , Puighermanal, E. , Castell, L. , Tarot, P. , Bertaso, F. , Bonnavion, P. , de Kerchove d'Exaerde, A. , Isingrini, E. , Galante, M. , Dallerac, G. , Pascoli, V. , Lüscher, C. , Giros, B. , & Valjent, E. (2021). Regulation of GluA1 phosphorylation by d‐amphetamine and methylphenidate in the cerebellum. Addiction Biology, 26(4), e12995. 10.1111/adb.12995 33368923

[tjp70833-bib-0014] de Jong, J. W. , Roelofs, T. J. M. , Mol, F. M. U. , Hillen, A. E. J. , Meijboom, K. E. , Luijendijk, M. C. M. , van der Eerden, H. A. M. , Garner, K. M. , Vanderschuren, L. J. M. J. , & Adan, R. A. H. (2015). Reducing ventral tegmental dopamine D2 receptor expression selectively boosts incentive motivation. Neuropsychopharmacology, 40, 2085–2095. 10.1038/npp.2015.60 PMC4613606 25735756

[tjp70833-bib-0015] Delignat‐Lavaud, B. , Kano, J. , Ducrot, C. , Massé, I. , Mukherjee, S. , Giguère, N. , Moquin, L. , Lévesque, C. , Burke, S. , Denis, R. , Bourque, M.‐J. , Tchung, A. , Rosa‐Neto, P. , Lévesque, D. , De Beaumont, L. , & Trudeau, L.‐É. (2023). Synaptotagmin‐1‐dependent phasic axonal dopamine release is dispensable for basic motor behaviours in mice. Nature Communications, 14(1), 4120. 10.1038/s41467-023-39805-7 PMC10336101 37433762

[tjp70833-bib-0016] Ducrot, C. , de Carvalho, G. , Delignat‐Lavaud, B. , Delmas, C. V. L. , Halder, P. , Giguère, N. , Pacelli, C. , Mukherjee, S. , Bourque, M.‐J. , Parent, M. , Chen, L. Y. , & Trudeau, L.‐E. (2023). Conditional deletion of neurexins dysregulates neurotransmission from dopamine neurons. eLife, 12, e87902. 10.7554/eLife.87902 PMC10409506 37409563

[tjp70833-bib-0017] Duvarci, S. (2024). Dopaminergic circuits controlling threat and safety learning. Trends in Neuroscience, 47(12), 1014–1027. 10.1016/j.tins.2024.10.001 39472156

[tjp70833-bib-0018] Flourakis, M. , Kula‐Eversole, E. , Hutchison, A. L. , Han, T. H. , Aranda, K. , Moose, D. L. , White, K. P. , Dinner, A. R. , Lear, B. C. , Ren, D. , Diekman, C. O. , Raman, I. M. , & Allada, R. (2015). A conserved bicycle model for circadian clock control of membrane excitability. Cell, 162(4), 836–848. 10.1016/j.cell.2015.07.036 PMC4537776 26276633

[tjp70833-bib-0018a] Franklin, K. , & Paxinos, G. (2008). The Mouse Brain in Stereotaxic Coordinates. 3rd ed. Academic Press.

[tjp70833-bib-0019] Gainetdinov, R. R. , Bohn, L. M. , Sotnikova, T. D. , Cyr, M. , Laakso, A. , Macrae, A. D. , Torres, G. E. , Kim, K.‐M. , Lefkowitz, R. J. , Caron, M. G. , & Premont, R. T. (2003). Dopaminergic supersensitivity in G protein‐coupled receptor kinase 6‐deficient mice. Neuron, 38(2), 291–303. 10.1016/s0896-6273(03)00192-2 12718862

[tjp70833-bib-0020] Gantz, S. C. , Ford, C. P. , Morikawa, H. , & Williams, J. T. (2018). The evolving understanding of dopamine neurons in the Substantia Nigra and ventral tegmental area. Annual Review of Physiology, 80(1), 219–241. 10.1146/annurev-physiol-021317-121615 28938084

[tjp70833-bib-0021] Gatley, S. J. , Pan, D. , Chen, R. , Chaturvedi, G. , & Ding, Y.‐S. (1996). Affinities of methylphenidate derivatives for dopamine, norepinephrine and serotonin transporters. Life Sciences, 58(12), PL231–PL239. 10.1016/0024-3205(96)00052-5 8786705

[tjp70833-bib-0022] Giros, B. , Jaber, M. , Jones, S. R. , Wightman, R. M. , & Caron, M. G. (1996). Hyperlocomotion and indifference to cocaine and amphetamine in mice lacking the dopamine transporter. Nature, 379(6566), 606–612. 10.1038/379606a0 8628395

[tjp70833-bib-0023] Gordon, J. H. , & Fields, J. Z. (1989). A permanent dopamine receptor up‐regulation in the ovariectomized rat. Pharmacology Biochemistry and Behavior, 33(1), 123–125. 10.1016/0091-3057(89)90440-1 2780771

[tjp70833-bib-0024] Gore, B. B. , Soden, M. E. , & Zweifel, L. S. (2014). Visualization of plasticity in fear‐evoked calcium signals in midbrain dopamine neurons. Learning & Memory, 21(11), 575–579. 10.1101/lm.036079.114 PMC4201808 25320348

[tjp70833-bib-0025] Hahn, S. , Um, K. B. , Kim, S. W. , Kim, H. J. , & Park, M. K. (2023). Proximal dendritic localization of NALCN channels underlies tonic and burst firing in nigral dopaminergic neurons. The Journal of Physiology, 601(1), 171–193. 10.1113/JP283716 36398712

[tjp70833-bib-0026] Isingrini, E. , Perret, L. , Rainer, Q. , Sagueby, S. , Moquin, L. , Gratton, A. , & Giros, B. (2016). Selective genetic disruption of dopaminergic, serotonergic and noradrenergic neurotransmission: Insights into motor, emotional and addictive behaviour. Journal of Psychiatry & Neuroscience, 41(3), 169–181. 10.1503/jpn.150028 PMC4853208 26505143

[tjp70833-bib-0027] Jehasse, K. , Massotte, L. , Hartmann, S. , Vitello, R. , Ringlet, S. , Vitello, M. , Chua, H. C. , Pless, S. A. , Engel, D. , Liégeois, J.‐F. , Lakaye, B. , Roeper, J. , & Seutin, V. (2021). The gating pore blocker 1‐(2,4‐xylyl)guanidinium selectively inhibits pacemaking of midbrain dopaminergic neurons. Neuropharmacology, 197, 108722. 10.1016/j.neuropharm.2021.108722 34273387

[tjp70833-bib-0028] Jo, Y. S. , Heymann, G. , & Zweifel, L. S. (2018). Dopamine neurons reflect the uncertainty in fear generalization. Neuron, 100(4), 916–925.e3. 10.1016/j.neuron.2018.09.028 PMC6226002 30318411

[tjp70833-bib-0029] Jones, G. L. , Soden, M. E. , Knakal, C. R. , Lee, H. , Chung, A. S. , Merriam, E. B. , & Zweifel, L. S. (2015). A genetic link between discriminative fear coding by the lateral amygdala, dopamine, and fear generalization. eLife, 4, e08969. 10.7554/eLife.08969 PMC4621744 26402461

[tjp70833-bib-0030] Juarez, B. , Kong, M.‐S. , Jo, Y. S. , Elum, J. E. , Yee, J. X. , Ng‐Evans, S. , Cline, M. , Hunker, A. C. , Quinlan, M. A. , Baird, M. A. , Elerding, A. J. , Johnson, M. , Ban, D. , Mendez, A. , Goodwin, N. L. , Soden, M. E. , & Zweifel, L. S. (2023). Temporal scaling of dopamine neuron firing and dopamine release by distinct ion channels shape behaviour. Science Advances, 9(32), eadg8869. 10.1126/sciadv.adg8869 PMC10421029 37566654

[tjp70833-bib-0031] Khaliq, Z. M. , & Bean, B. P. (2010). Pacemaking in dopaminergic ventral tegmental area neurons: Depolarizing drive from background and voltage‐dependent sodium conductances. Journal of Neuroscience, 30(21), 7401–7413. 10.1523/JNEUROSCI.0143-10.2010 PMC2892804 20505107

[tjp70833-bib-0032] Khaliq, Z. M. , & Bean, B. P. (2008). Dynamic, nonlinear feedback regulation of slow pacemaking by A‐type potassium current in ventral tegmental area neurons. Journal of Neuroscience, 28(43), 10905–10917. 10.1523/JNEUROSCI.2237-08.2008 PMC2837275 18945898

[tjp70833-bib-0033] Lindgren, N. , Xu, Z.‐Q. D. , Herrera‐Marschitz, M. , Haycock, J. , Hökfelt, T. , & Fisone, G. (2001). Dopamine D(2) receptors regulate tyrosine hydroxylase activity and phosphorylation at Ser40 in rat striatum. European Journal of Neuroscience, 13(4), 773–780. 10.1046/j.0953-816x.2000.01443.x 11207812

[tjp70833-bib-0034] Lindgren, N. , Xu, Z.‐Q. D. , Lindskog, M. , Herrera‐Marschitz, M. , Goiny, M. , Haycock, J. , Goldstein, M. , Hökfelt, T. , & Fisone, G. (2000). Regulation of tyrosine hydroxylase activity and phosphorylation at ser(19) and ser(40) via activation of glutamate NMDA receptors in rat striatum. Journal of Neurochemistry, 74(6), 2470–2477. 10.1046/j.1471-4159.2000.0742470.x 10820208

[tjp70833-bib-0035] Liss, B. , Franz, O. , Sewing, S. , Bruns, R. , Neuhoff, H. , & Roeper, J. (2001). Tuning pacemaker frequency of individual dopaminergic neurons by Kv4.3L and KChip3.1 transcription. EMBO Journal, 20(20), 5715–5724. 10.1093/emboj/20.20.5715 PMC125678 11598014

[tjp70833-bib-0036] Liss, B. , & Roeper, J. (2008). Individual dopamine midbrain neurons: Functional diversity and flexibility in health and disease. Brain Research Reviews, 58(2), 314–321. 10.1016/j.brainresrev.2007.10.004 18023878

[tjp70833-bib-0037] Lu, B. , Su, Y. , Das, S. , Wang, H. , Wang, Y. , Liu, J. , & Ren, D. (2009). Peptide neurotransmitters activate a cation channel complex of NALCN and UNC‐80. Nature, 457(7230), 741–744. 10.1038/nature07579 PMC2810458 19092807

[tjp70833-bib-0038] Luo, R. , Uematsu, A. , Weitemier, A. , Aquili, L. , Koivumaa, J. , McHugh, T. J. , & Johansen, J. P. (2018). A dopaminergic switch for fear to safety transitions. Nature Communications, 9(1), 2483. 10.1038/s41467-018-04784-7 PMC6021378 29950562

[tjp70833-bib-0039] McCall, N. M. , Kotecki, L. , Dominguez‐Lopez, S. , Marron Fernandez de Velasco, E. , Carlblom, N. , Sharpe, A. L. , Beckstead, M. J. , & Wickman, K. (2017). Selective ablation of GIRK channels in dopamine neurons alters behavioural effects of cocaine in mice. Neuropsychopharmacology, 42(3), 707–715. 10.1038/npp.2016.138 PMC5240170 27468917

[tjp70833-bib-0040] McCall, N. M. , Marron Fernandez de Velasco, E. , & Wickman, K. (2019). GIRK channel activity in dopamine neurons of the ventral tegmental area bidirectionally regulates behavioural sensitivity to cocaine. Journal of Neuroscience, 39(19), 3600–3610. 10.1523/JNEUROSCI.3101-18.2019 PMC6510348 30837265

[tjp70833-bib-0041] McCutcheon, J. E. , Beeler, J. A. , & Roitman, M. F. (2012). Sucrose‐predictive cues evoke greater phasic dopamine release than saccharin‐predictive cues. Synapse, 66(4), 346–351. 10.1002/syn.21519 PMC3269555 22170625

[tjp70833-bib-0042] Philippart, F. , & Khaliq, Z. M. (2018). Gi/o protein‐coupled receptors in dopamine neurons inhibit the sodium leak channel NALCN. eLife, 7, e40984. 10.7554/eLife.40984 PMC6305199 30556810

[tjp70833-bib-0043] Puighermanal, E. , Castell, L. , Esteve‐Codina, A. , Melser, S. , Kaganovsky, K. , Zussy, C. , Boubaker‐Vitre, J. , Gut, M. , Rialle, S. , Kellendonk, C. , Sanz, E. , Quintana, A. , Marsicano, G. , Martin, M. , Rubinstein, M. , Girault, J.‐A. , Ding, J. B. , & Valjent, E. (2020). Functional and molecular heterogeneity of D2R neurons along dorsal ventral axis in the striatum. Nature Communications, 11(1), 1957. 10.1038/s41467-020-15716-9 PMC7181842 32327644

[tjp70833-bib-0044] Puopolo, M. , Raviola, E. , & Bean, B. P. (2007). Roles of subthreshold calcium current and sodium current in spontaneous firing of mouse midbrain dopamine neurons. Journal of Neuroscience, 27(3), 645–656. 10.1523/JNEUROSCI.4341-06.2007 PMC6672803 17234596

[tjp70833-bib-0045] Ramsey, A. J. , Laakso, A. , Cyr, M. , Sotnikova, T. D. , Salahpour, A. , Medvedev, I. O. , Dykstra, L. A. , Gainetdinov, R. R. , & Caron, M. G. (2008). Genetic NMDA receptor deficiency disrupts acute and chronic effects of cocaine but not amphetamine. Neuropsychopharmacology, 33(11), 2701–2714. 10.1038/sj.npp.1301663 PMC5698087 18185498

[tjp70833-bib-0046] Roeper, J. (2013). Dissecting the diversity of midbrain dopamine neurons. Trends in Neuroscience, 36(6), 336–342. 10.1016/j.tins.2013.03.003 23582338

[tjp70833-bib-0047] Salinas‐Hernández, X. I. , Zafiri, D. , Sigurdsson, T. , & Duvarci, S. (2023). Functional architecture of dopamine neurons driving fear extinction learning. Neuron, 111(23), 3854–3870.e5. 10.1016/j.neuron.2023.08.025 37741275

[tjp70833-bib-0048] Schultz, W. (2016). Dopamine reward prediction‐error signalling: A two‐component response. Nature Reviews Neuroscience, 17(3), 183–195. 10.1038/nrn.2015.26 PMC5549862 26865020

[tjp70833-bib-0049] Schultz, W. (1998). Predictive reward signal of dopamine neurons. Journal of Neurophysiology, 80(1), 1–27. 10.1152/jn.1998.80.1.1 9658025

[tjp70833-bib-0050] Sun, F. , Zeng, J. , Jing, M. , Zhou, J. , Feng, J. , Owen, S. F. , Luo, Y. , Li, F. , Wang, H. , Yamaguchi, T. , Yong, Z. , Gao, Y. , Peng, W. , Wang, L. , Zhang, S. , Du, J. , Lin, D. , Xu, M. , Kreitzer, A. C. , Cui, G. , & Li, Y. (2018). A genetically encoded fluorescent sensor enables rapid and specific detection of dopamine in flies, fish, and mice. Cell, 174(2), 481–496.e19. 10.1016/j.cell.2018.06.042 PMC6092020 30007419

[tjp70833-bib-0051] Szumlinski, K. K. , Dehoff, M. H. , Kang, S. H. , Frys, K. A. , Lominac, K. D. , Klugmann, M. , Rohrer, J. , Griffin, W. , Toda, S. , Champtiaux, N. P. , Berry, T. , Tu, J. C. , Shealy, S. E. , During, M. J. , Middaugh, L. D. , Worley, P. F. , & Kalivas, P. W. (2004). Homer proteins regulate sensitivity to cocaine. Neuron, 43(3), 401–413. 10.1016/j.neuron.2004.07.019 15294147

[tjp70833-bib-0052] Thompson, T. L. , & Moss, R. L. (1994). Estrogen regulation of dopamine release in the nucleus accumbens: Genomic‐ and nongenomic‐mediated effects. Journal of Neurochemistry, 62(5), 1750–1756. 10.1046/j.1471-4159.1994.62051750.x 8158125

[tjp70833-bib-0053] Topalidou, I. , Cooper, K. , Pereira, L. , & Ailion, M. (2017). Dopamine negatively modulates the NCA ion channels in C. elegans. PLoS Genetics, 13(10), e1007032. 10.1371/journal.pgen.1007032 PMC5638609 28968387

[tjp70833-bib-0054] Um, K. B. , Hahn, S. , Kim, S. W. , Lee, Y. J. , Birnbaumer, L. , Kim, H. J. , & Park, M. K. (2021). TRPC3 and NALCN channels drive pacemaking in substantia nigra dopaminergic neurons. eLife, 10, e70920. 10.7554/eLife.70920 PMC8456572 34409942

[tjp70833-bib-0055] Usher, S. , Toulmé, E. , Florea, R. , Yatskevich, S. , Jao, C. C. , Dijkhof, L. R. H. , Colding, J. M. , Joshi, P. , Zilberleyb, I. , Trimbuch, T. , Brokowski, B. , Hauser, A. S. , Leitner, A. , Rosenmund, C. , Kschonsak, M. , & Pless, S. A. (2025). The sodium leak channel NALCN is regulated by neuronal SNARE complex proteins. Science Advances, 11(11), eads6004. 10.1126/sciadv.ads6004 PMC13109934 40085699

[tjp70833-bib-0056] Valjent, E. , Biever, A. , Gangarossa, G. , & Puighermanal, E. (2019). Dopamine signaling in the striatum. Advances in Protein Chemistry and Structural Biology, 116, 375–396. 10.1016/bs.apcsb.2019.01.004 31036297

[tjp70833-bib-0057] Vandegrift, B. J. , You, C. , Satta, R. , Brodie, M. S. , & Lasek, A. W. (2017). Estradiol increases the sensitivity of ventral tegmental area dopamine neurons to dopamine and ethanol. PLoS ONE, 12(11), e0187698. 10.1371/journal.pone.0187698 PMC5673180 29107956

[tjp70833-bib-0058] Wang, J. , Su, M. , Zhang, D. , Zhang, L. , Niu, C. , Li, C. , You, S. , Sang, Y. , Zhang, Y. , Du, X. , & Zhang, H. (2024). The cation channel mechanisms of subthreshold inward depolarizing currents in the mice VTA dopaminergic neurons and their roles in the chronic‐stress‐induced depression‐like behaviour. eLife, 12, RP88319. 10.7554/eLife.88319 PMC11623934 39642080

[tjp70833-bib-0059] Wolfart, J. , Neuhoff, H. , Franz, O. , & Roeper, J. (2001). Differential expression of the small‐conductance, calcium‐activated potassium channel SK3 is critical for pacemaker control in dopaminergic midbrain neurons. Journal of Neuroscience, 21(10), 3443–3456. 10.1523/JNEUROSCI.21-10-03443.2001 PMC6762487 11331374

[tjp70833-bib-0060] Zafiri, D. , Salinas‐Hernández, X. I. , De Biasi, E. S. , Rebelo, L. , & Duvarci, S. (2025). Dopamine prediction error signaling in a unique nigrostriatal circuit is critical for associative fear learning. Nature Communications, 16(1), 3066. 10.1038/s41467-025-58382-5 PMC11954928 40157963

[tjp70833-bib-0061] Zweifel, L. S. , Fadok, J. P. , Argilli, E. , Garelick, M. G. , Jones, G. L. , Dickerson, T. M. K. , Allen, J. M. , Mizumori, S. J. Y. , Bonci, A. , & Palmiter, R. D. (2011). Activation of dopamine neurons is critical for aversive conditioning and prevention of generalized anxiety. Nature Neuroscience, 14(5), 620–626. 10.1038/nn.2808 PMC3083461 21499253

[tjp70833-bib-0062] Zweifel, L. S. , Parker, J. G. , Lobb, C. J. , Rainwater, A. , Wall, V. Z. , Fadok, J. P. , Darvas, M. , Kim, M. J. , Mizumori, S. J. Y. , Paladini, C. A. , Phillips, P. E. M. , & Palmiter, R. D. (2009). Disruption of NMDAR‐dependent burst firing by dopamine neurons provides selective assessment of phasic dopamine‐dependent behaviour. Proceedings of the National Academy of Sciences of the United States of America, 106(18), 7281–7288. 10.1073/pnas.0813415106 PMC2678650 19342487

