# Supplementary material for: Deletion of the sodium leak channel NALCN in dopamine neurons alters D2R‐mediated signalling and psychostimulant responsiveness
Source: J Physiol. 2026 Oct 6;604(20):8542–68. doi: 10.1113/JP289809 (PMC13674192; doi:10.1113/JP289809)
Supplement: Supplementary file 1 [file TJP-604-8542-s001.pdf]

The Journal of Physiology

Deletion of the sodium leak channel NALCN in dopamine neurons alters D2R-mediated signaling and psychostimulant responsiveness

Angelina Rogliardo, Tinaig Le Borgne, Aylin Gulmez, Adeline Cathala, Claire Naon, Audrey Mignon, Maelle Avrillon, Nejmeh Mashhour, Laia Castell, Claire Bernat, Jean-Michel Revest, Arnaud Monteil, Philippe Faure, Federica Bertaso, Giuseppe Gangarossa, Fabio Marti, and Emmanuel Valjent
DOI: 10.1113/JP289809

Corresponding author(s): Angelina Rogliardo (angelina.rogliardo@inserm.fr)

The following individual(s) involved in review of this submission have agreed to reveal their identity: Gordon W Arbuthnott (Referee #1); Hailin Zhang (Referee #2)

Review Timeline:

Submission Date:	29-Jul-2025
Editorial Decision:	10-Sep-2025
Revision Received:	07-Jul-2026
Accepted:	05-Aug-2026

Senior Editor: Jing-Ning Zhu

Reviewing Editor: Wei-Guang Li

Transaction Report:

(Note: With the exception of the correction of typographical or spelling errors that could be a source of ambiguity, letters and reports are not edited. Depending on transfer agreements, referee reports obtained elsewhere may or may not be included in this compilation. Referee reports are anonymous unless the Referee chooses to sign their reports.)

Dear Dr Rogliardo,

Re: JP-RP-2025-289809 "New insight into the sodium leak channel NALCN : at the crossroad of pacemaker activity, dopamine release and psychostimulant responsiveness" by Angelina Rogliardo, Tinaig Le Borgne, Claire Naon, Adeline Cathala, Nejmeh Mashhour, Laia Castell, Claire Bernat, Jean-Michel Revest, Arnaud Monteil, Philippe Faure, Federica Bertaso, Giuseppe Gangarossa, Fabio Marti, and Emmanuel Valjent

Thank you for submitting your manuscript to The Journal of Physiology. It has been assessed by a Reviewing Editor and by 2 expert referees and we are pleased to tell you that it is potentially acceptable for publication following satisfactory major revision.

Please advise your co-authors of this decision as soon as possible.

The referee reports are copied at the end of this email.

Please address all the points raised and incorporate all requested revisions or explain in your Response to Referees why a change has not been made. We hope you will find the comments helpful and that you will be able to return your revised manuscript within 9 months. If you require longer than this, please contact journal staff: jp@physoc.org. Please note that this letter does not constitute a guarantee for acceptance of your revised manuscript.

Your revised manuscript should be submitted online using the link in your Author Tasks: Link Not Available. This link is accessible via your account as Corresponding Author; it is not available to your co-authors. If this presents a problem, please contact journal staff (jp@physoc.org). Image files from the previous version are retained on the system. Please ensure you replace or remove any files that are being revised.

If you do not wish to submit a revised version of your manuscript, you must inform our journal staff (jp@physoc.org) or reply to this email to request withdrawal. Please note that a manuscript must be formally withdrawn from the peer review process at one journal before it may be submitted to another journal.

TRANSPARENT PEER REVIEW POLICY: To improve the transparency of its peer review process, The Journal of Physiology publishes online as supporting information the peer review history of all articles accepted for publication. Readers will have access to decision letters, including Editors' comments and referee reports, for each version of the manuscript, as well as any author responses to peer review comments. Referees can decide whether or not they wish to be named on the peer review history document.

ABSTRACT FIGURES: Authors are expected to use The Journal's premium BioRender account to create/redraw their Abstract Figures. Information on how to access this account is here: https://physoc.onlinelibrary.wiley.com/journal/14697793/biorender-access.

This will enable Authors to create and download high-resolution figures. If authors have used the free BioRender service, they can use the instructions provided in the link above to download a high-resolution version suitable for publication.

The link provided should only be used for the purposes of this submission. Authors will be charged for figures created on this account if they are not related to this manuscript submission.

LANGUAGE EDITING AND SUPPORT FOR PUBLICATION: If you would like help with English language editing, or other article preparation support, Wiley Editing Services offers expert help, including English Language Editing, as well as translation, manuscript formatting, and figure formatting at www.wileyauthors.com/eeo/preparation. You can also find resources for Preparing Your Article for general guidance about writing and preparing your manuscript at www.wileyauthors.com/eeo/prepresources.

RIGOUR AND REPRODUCIBILITY: We recommend authors consult The Journal's Rigour and Reproducibility requirements to ensure that their manuscript is suitable for publication: https://physoc.onlinelibrary.wiley.com/pb-assets/hub-assets/physoc/documents/TJP-Rigour-and-Reproducibility-Requirements.pdf

REVISION CHECKLIST:

Check that your Methods section conforms to journal policy: https://jp.msubmit.net/cgi-bin/main.plex?form_type=display_requirements#methods.

Check that data presented conforms to the statistics policy: https://jp.msubmit.net/cgi-bin/main.plex?form_type=display_requirements#statistics.

Upload a full Response to Referees file. To create your 'Response to Referees': copy all the reports, including any comments from the Senior and Reviewing Editors, into a Microsoft Word, or similar, file and respond to each point, using font or background colour to distinguish comments and responses and upload as the required file type.

Please upload two versions of your manuscript text: one with all relevant changes highlighted and one clean version with no changes tracked. The manuscript file should include all tables and figure legends, but each figure/graph should be uploaded as separate, high-resolution files.

You may also upload:

- 'Potential Cover Art' for consideration as the issue's cover image
- Appropriate Supporting Information (Video, audio or data set: see https://jp.msubmit.net/cgi-bin/main.plex?form_type=display_requirements#supp).

We look forward to receiving your revised submission.

If you have any queries, please reply to this email and we will be pleased to advise.

Yours sincerely,

Jing-Ning Zhu
Senior Editor
The Journal of Physiology

REQUIRED ITEMS

- Author photo and profile. First or joint first authors are asked to provide a short biography (no more than 100 words for one author or 150 words in total for joint first authors) and a portrait photograph. These should be uploaded and clearly labelled together in a Word document with the revised version of the manuscript. See Information for Authors for further details.

- You must start the Methods section with a paragraph headed Ethical approval (https://jp.msubmit.net/cgi-bin/main.plex?form_type=display_requirements#methods).

Research must comply with The Journal's policies regarding animal experiments (https://physoc.onlinelibrary.wiley.com/hub/animal-experiments) and adherence to these policies must be stated in the manuscript.

Authors should confirm in their Methods section that their experiments were carried out according to the guidelines laid down by their institution's animal welfare committee, including an ethics approval reference number. The Methods section must contain a statement about access to food, water and housing, details of the anaesthetic regime: anaesthetic used, dose and route of administration, and method of killing the experimental animals.

- The reference list must be in alphabetical order, rather than numbered, to comply with our Journal format.

- Please upload separate high-quality figure files via the submission form.

- You must upload original, uncropped western blot/gel images (including controls) if they are not included in the manuscript. This is to confirm that no inappropriate, unethical or misleading image manipulation has occurred. These should be uploaded as 'Supporting information for review process only'. Please label/highlight the original gels so that we can clearly see which sections/lanes have been used in the manuscript figures. For more information, see: https://physoc.onlinelibrary.wiley.com/hub/journal-policies#imagmanip.

- Please ensure that any tables are editable and in Word format, and wherever possible, embedded in the article file itself.

- Please ensure that the Article File you upload is a Word file.

- Your paper contains Supporting Information of a type that we no longer publish, including supplementary tables and figures. Any information essential to an understanding of the paper must be included as part of the main manuscript and figures. The only Supporting Information that we publish are video and audio, 3D structures, program codes and large data files. Your revised paper will be returned to you if it does not adhere to our Supporting Information Guidelines.

- A Data Availability Statement is required for all papers reporting original data. This must be in the Additional Information section of the manuscript itself. It must have the paragraph heading 'Data Availability Statement'. All data supporting the results in the paper must be either: in the paper itself; uploaded as Supporting Information for Online Publication; or archived in an appropriate public repository. The statement needs to describe the availability or the absence of shared data. Authors must include in their statement: a link to the repository they have used, or a statement that it is available as Supporting Information; reference the data in the appropriate sections(s) of their manuscript; and cite the data they have shared in the References section. Whenever possible, the scripts and other artefacts used to generate the analyses presented in the paper should also be publicly archived. If sharing data compromises ethical standards or legal requirements then authors are not expected to share it, but must note this in their statement. For more information, see our Statistics Policy.

- Please include an Abstract Figure file, as well as the Figure Legend text within the main article file. The Abstract Figure is a piece of artwork designed to give readers an immediate understanding of the research and should summarise the main conclusions. If possible, the image should be easily 'readable' from left to right or top to bottom. It should show the physiological relevance of the manuscript so readers can assess the importance and content of its findings. Abstract Figures should not merely recapitulate other figures in the manuscript. Please try to keep the diagram as simple as possible and without superfluous information that may distract from the main conclusion(s). Abstract Figures must be provided by authors no later than the revised manuscript stage and should be uploaded as a separate file during online submission labelled as File Type 'Abstract Figure'. Please also ensure that you include the figure legend in the main article file. All Abstract Figures should be created using BioRender. Authors should use The Journal's premium BioRender account to export high-resolution images. Details on how to use and access the premium account are included as part of this email.

EDITOR COMMENTS

Reviewing Editor:

The author presents an intriguing dissociation-near-silenced VTA dopamine-neuron pacemaking with preserved basal accumbal dopamine and altered psychostimulant responses-that could be influential, but acceptance requires Major Revision: the author should tighten the pacemaker characterization (in vivo loss of both tonic and burst firing), provide direct terminal evidence for impaired auto-D2R signaling (e.g., FSCV/dLight or local microdialysis), reconcile inconsistencies across psychostimulants (KO-male hyperlocomotion without DA elevation vs. methylphenidate/GBR), validate DA-neuron-specific NALCN deletion (e.g., TH/Nalcn RNAscope, Western blot, or rescue), broaden behavior to include a positively reinforced operant assay or temper claims, analyze sex effects, and improve figure quality/annotation while acknowledging the temporal limits of microdialysis.

Please also see 'Required Items' above.

Senior Editor:

The manuscript offers a technically broad investigation into the role of NALCN in midbrain dopamine neuron function, revealing a striking dissociation between disrupted pacemaker activity and preserved basal dopamine release. While the findings are of potential interest to the field, both Reviewers and the Reviewing Editor have identified several substantial concerns that must be addressed before the work can be considered further. These include a need for clearer and more accurate interpretation of the in vivo electrophysiological data, reconciliation of the mechanistic disconnect between neuronal silencing and sustained dopamine output, and clarification of inconsistencies across the psychostimulant response data. Additional validation of conditioning knockout, inclusion of positively reinforced behavioural assays, and improvements in figure presentation and textual clarity are also required.

REFEREE COMMENTS

Referee #1:

This carefully executed investigation reaches some surprising conclusions. It seems that greatly disturbing the resting electrophysiology of the dopamine cells has little effect on normal behaviour. The surprise that a near abolition of resting firing in dopamine cells leads to normal levels of dopamine release, in the forebrain, emphasises the importance of other mechanisms in control of the dopamine release in those terminals. In contrast, but perhaps not quite so surprising is that the pharmacological control of the release is still active although drug by sex contrasts exist in the sensitivity to individual stimulants. Although the authors emphasise the actions of dopamine on D2 autoreceptors, there may be other explanations, some of which they do include in the discussion.

One significant absence in the paper is any attempt to asses the effects on operant behaviours led by positive reinforcement. These escape behaviours are less well associated with direct reinforcing effects on which the role of dopamine in behavioural control have been based. Sure escape from danger is one kind of behaviour that is 'reinforcing' but simple positive reinforcement directly linked to a learned behaviour is not addressed. Of course the results might be the same, but any distinction between positive reinforcement and 'escape' is not discussed.

Some small errors that might distract from the arguments in the paper - but I am no expert in proof reading!

Third last line on the second paragraph on p6...Despite these results ..., OR ..that evidence...

Last line on p16 ...NALCN is required for normal pacemaker activity...

Third last line on p25....delete 'not' the experiments suggest... pacemaking activity is dispensable for...

Third last line on p27 L.C. Set up ...not 'settled up'...

Referee #2:

In this manuscript titled "New insight into the sodium leak channel NALCN: at the crossroad of pacemaker activity, dopamine release and psychostimulant responsiveness", Rogliardo et al., have performed electrophysiology patch-clamp recordings, behavioral analyses, microdialysis, immunological assays, and demonstrated the impact of NALCN deletion on tonic activity of VTA DA neurons, D2R-mediated signalling, reactivity to cues-predicting threat and psychostimulants responsiveness. This study contains some interesting findings and is valuable for understanding the diffenential impact of NALCN channel in midbrain DA neurons at the behavioral level. However, several methodological and interpretational limitations must be addressed to support the conclusions.

Major issues:

1. Via extracellular single-unit recordings, DA cells fired with a continuum of firing patterns between two extreme cases corresponding to tonic or burst firing pattern. In the Fig.1b, all DA neurons in Nalcnslc6a3 mice-except one-are silent, lacking both tonic and burst firing activity. This makes the conclusion drawn from Fig.1 misleading. The data basically demonstrate a near-complete loss of both tonic activity and burs firing, not only tonic firing.

2. In Fig.2a, the authors report no difference in basal DA levels in the nucleus accumben of Nalcnslc6a3 and control mice. This raises a critical question: How do the silent DA neurons in Nalcnslc6a3 mice release DA at levels comparable to controls? The authors must explain this discrepancy between nearly-silent neuronal firing and unaltered basal DA release.

3. It is not sufficient to make the conclusion that D2R-mediated signaling at DA terminals is impaired in Nalcnslc6a3 mice from the indirect experimental data presented in Fig.3. Simuilarlly, it is not convinced that Nalcnslc6a3 male mice display supersensitivity to the psychomotor effects of cocaine via the impairment of autoD2R signaling.

4. A key paradox emerges: Cocaine induces hyperlocomotion in knockout (KO) males (Fig. 5a), yet no increase in DA release is observed (Fig. 5b). The authors attribute this to "altered D2R signaling", but responses to methylphenidate/GBR12783 (Figs. 5e-h) contradict this mechanism. For instance, if D2R dysfunction were the cause,

methylphenidate (which also acts on DA transporters) should elicit a similar hyperlocomotive phenotype-but the data do not support this. The authors should explain this inconsistency.

5. The title positions NALCN at the "crossroad" of pacemaking, DA release, and psychostimulant responses, but pacemaker activity is only superficially characterized. Furthermore, the observed alterations in psychostimulant responses are attributed to D2R dysfunction rather than pacemaker deficits-undermining the "crossroad" framing. The title or data should be revised to align with this mechanistic focus.

Other issues:

1. The manuscript lacks validation data (e.g., Western blot, immunofluorescence) confirming NALCN knockout efficiency in DA neurons. Phenotypic effects cannot be definitively attributed to NALCN loss without this test.

2. Cocaine hyper-locomotion occurs only in KO males (Fig. 5a vs. 5c), but hormonal influences are unexplored. Discuss potential estrogen modulation of DA excitability (e.g., via D2R expression).

3. Microdialysis measures tonic DA (minute-scale resolution), missing phasic signals critical for psychostimulant responses.

4. Several figures are pixelated or poorly annotated.

5. Typos exist (e.g., "VTA= DA-neurons"). Correct grammatical errors and clarify phrasing.

END OF COMMENTS

EDITOR COMMENTS

Reviewing Editor:

The author presents an intriguing dissociation-near-silenced VTA dopamine-neuron pacemaking with preserved basal accumbal dopamine and altered psychostimulant responses-that could be influential, but acceptance requires Major Revision: the author should tighten the pacemaker characterization (in vivo loss of both tonic and burst firing), provide direct terminal evidence for impaired auto-D2R signaling (e.g., FSCV/dLight or local microdialysis), reconcile inconsistencies across psychostimulants (KO-male hyperlocomotion without DA elevation vs. methylphenidate/GBR), validate DA-neuron-specific NALCN deletion (e.g., TH/Nalcn RNAscope, Western blot, or rescue), broaden behavior to include a positively reinforced operant assay or temper claims, analyze sex effects, and improve figure quality/annotation while acknowledging the temporal limits of microdialysis.

Senior Editor:

The manuscript offers a technically broad investigation into the role of NALCN in midbrain dopamine neuron function, revealing a striking dissociation between disrupted pacemaker activity and preserved basal dopamine release. While the findings are of potential interest to the field, both Reviewers and the Reviewing Editor have identified several substantial concerns that must be addressed before the work can be considered further. These include a need for clearer and more accurate interpretation of the in vivo electrophysiological data, reconciliation of the mechanistic disconnect between neuronal silencing and sustained dopamine output, and clarification of inconsistencies across the psychostimulant response data. Additional validation of conditioning knockout, inclusion of positively reinforced behavioural assays, and improvements in figure presentation and textual clarity are also required.

We thank the Reviewing and Senior Editors for their positive and constructive comments. We have extensively revised our manuscript according to their valuable suggestions that we have now incorporated into the revised manuscript. Here is a list of the major additional experiments and modifications made to comply with the reviewers' suggestions:

- We have included new *in vivo* electrophysiological data as well as measures of DA dynamics using fiber photometry experiments (revised Fig. 1 and 3). Together, these new sets of data demonstrate that, despite the profound alterations in DA neuron activity observed under anesthetized conditions, tonic DA tone persists in awake *Nalcn*^{Slc6a3} male and female mice. These data allow us to draw a more accurate interpretation and refine our conclusion.
- We have now carried out a series of experiments that further consolidate that autoD2R signaling is impaired. Additional microdialysis experiments performed in both male and female mice (revised Fig. 3) indicated that the increase of extracellular accumbal DA level induced by a single administration of the D2R antagonist eticlopride is blunted in both *Nalcn*^{Slc6a3} male and female mice. These results were further consolidated by measuring accumbal DA dynamics using fiber photometry following single administration of the D2R agonist, quinpirole and the D2R antagonist, eticlopride. The effects of both quinpirole and eticlopride were markedly reduced in *Nalcn*^{Slc6a3} mice compared with control mice indicating an impairment of D2 receptor-mediated regulation of DA transmission in neurons lacking NALCN channels. These new data have been included and discussed in the revised version.

- Regarding the psychostimulant response data, higher doses of methylphenidate (10 mg/kg) and GBR12783 (30 mg/kg) have been tested (revised Figure 8). Our results revealed that when GBR12783 was administered at 30 mg/kg, only female *Nalcn*^{Slc6a3} mice maintained higher locomotor responses between 30 and 90 min. For methylphenidate, the psychomotor effects induced by methylphenidate at a dose of 10 mg/kg tended to be more pronounced in both male and female *Nalcn*^{Slc6a3} mice as compared to controls. These results confirmed our initial observation that enhanced psychomotor responses induced by cocaine were not recapitulated by a single administration of the selective blockade of the DA transporter or methylphenidate. These data further consolidate the idea that enhanced locomotion induced by cocaine might occur independently of heightened DA release in the nucleus accumbens. As discussed in the revised version, this phenotype closely resembles that reported in mouse models lacking G protein-coupled receptor kinase 6 (PMID: 12718862), Homer1 and Homer2 (PMID: 15294147), or the norepinephrine transporter (PMID: 10769386). Importantly, we also discussed evidence in the literature indicating that hyperlocomotion and DA release can be uncorrelated as illustrated in mice with decreased expression of the NR1 subunit of the NMDAR (PMID: 18185498) or lacking metabotropic glutamate receptor 5 (PMID: 11528416).
- As mentioned in the responses to referee, we did perform operant behaviors in *Nalcn*^{Slc6a3} mice and were able to show that they equally performed the task earning similar amount of rewards under FR1, FR5 and PR reinforcement schedule. However, this set of data cannot be included in the present study as they were already part of another manuscript which has been recently published (PMID: 41673358). Nevertheless, we carried out another set of experiments to assess appetitive responses triggered by natural rewards (revised Figure 7). We performed a 2-h choice test during which control and *Nalcn*^{Slc6a3} mice from both sexes can choose between i) water or sucrose solution, ii) water or saccharin solution or between iii) sucrose and saccharin solution. In both males and females, we found that control and *Nalcn*^{Slc6a3} mice showed a similar preference toward sucrose and saccharin and consumed the same volume of liquid, indicating that the appetence of solutions with high hedonic value is unaltered in NalcnSlc6a3 mice. These new data are now presented and discussed in light of the results of the operant data in the revised manuscript.
- Figures at high resolution have been now provided.

REFEREE COMMENTS

Referee #1:

This carefully executed investigation reaches some surprising conclusions. It seems that greatly disturbing the resting electrophysiology of the dopamine cells has little effect on normal behaviour. The surprise that a near abolition of resting firing in dopamine cells leads to normal levels of dopamine release, in the forebrain, emphasises the importance of other mechanisms in control of the dopamine release in those terminals. In contrast, but perhaps not quite so surprising is that the pharmacological control of the release is still active although drug by sex contrasts exist in the sensitivity to individual stimulants. Although the authors emphasise the actions of dopamine on D2 autoreceptors, there may be other explanations, some of which they do include in the discussion.

We thank the reviewer for his/her positive comments. We have now carried out a series of experiments that further consolidate that auto-D2R signaling is impaired. Additional microdialysis experiments performed in both male and female mice indicated that the increase of extracellular accumbal DA level induced by a single administration of the D2R antagonist eticlopride is blunted in both *Nalcn*^{Slc6a3} male and female mice (**Figure 3g**). These results were further consolidated by measuring accumbal DA dynamics using fiber photometry following single administration of the D2R agonist, quinpirole and the D2R antagonist, eticlopride. As shown now in **Figure 3e-f**, the effects of both quinpirole and eticlopride were markedly reduced in *Nalcn*^{Slc6a3} mice compared with control mice, indicating an impairment of D2 receptor-mediated regulation of DA transmission in neurons lacking NALCN channels. These new results obtained using fiber photometry allowed to strength our conclusion and refine our interpretation. Indeed, quinpirole still induced a significant reduction in the $GRAB_{DA3m}$ signal in *Nalcn*^{Slc6a3} mice. Because activation of autoD2Rs reduces DA release, such an effect could only be observed if dopamine was being tonically released before drug administration. These results therefore clearly demonstrate that despite the profound alterations in DA neuron activity observed under anesthetized conditions, tonic DA tone persists in awake *Nalcn*^{Slc6a3} male and female mice. We therefore agree with the reviewer that mechanisms other than intrinsic pacemaker activity contribute substantially to the control of dopamine release at forebrain terminals. Indeed, under anesthetized conditions, dopamine neuron activity relies predominantly on intrinsic pacemaker mechanisms, whereas in awake animals ongoing network activity and synaptic inputs may sustain dopamine transmission despite marked alterations in intrinsic excitability. This issue is now discussed and clarified in the revised version.

One significant absence in the paper is any attempt to asses the effects on operant behaviours led by positive reinforcement. These escape behaviours are less well associated with direct reinforcing effects on which the role of dopamine in behavioural control have been based. Sure escape from danger is one kind of behaviour that is 'reinforcing' but simple positive reinforcement directly linked to a learned behaviour is not addressed. Of course the results might be the same, but any distinction between positive reinforcement and 'escape' is not discussed.

We thank the reviewer for this interesting point. In fact, we have previously performed operant behavioral tests in *Nalcn*^{Slc6a3} male mice and were able to show that they equally performed the task earning similar amounts of rewards under FR1, FR5 and PR reinforcement schedule (**Figure below**). However, this set of data cannot be included in the present study as they were already part of another

manuscript which has been recently published (PMID: 41673358). These data are now mentioned in the revised version of the present study.

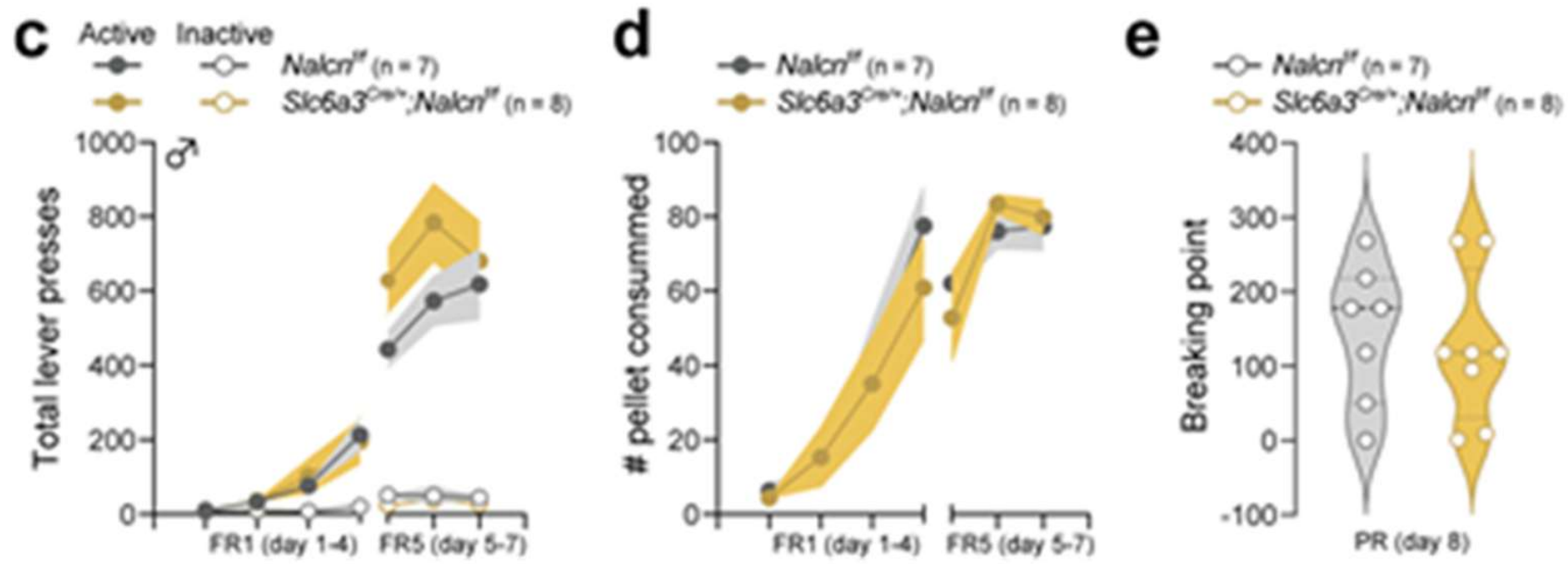

(**c**) Total lever presses for palatable food pellets on the active and inactive levers during FR1 (days 1-4) and FR5 (days 5-7) in *Nalcn$^{f/f}$* and *Slc6a3$^{Cre/+}$;Nalcn$^{f/f}$* mice. (**d**) Daily pellet consumption during FR1 and FR5 in *Nalcn$^{f/f}$* and *Slc6a3$^{Cre/+}$;Nalcn$^{f/f}$* mice. (**e**) Breaking point analysis under a PR reinforcement schedule in *Nalcn$^{f/f}$* and *Slc6a3$^{Cre/+}$;Nalcn$^{f/f}$* mice. FR: fixed-ratio; PR: progressive ratio. Supplemental material (PMID: 41673358)

Nevertheless, we carried out another set of experiments to assess appetitive responses triggered by natural rewards. We performed a 2-h choice test during which exposed control and *Nalcn^{Slc6a3}* mice from both sexes can choose between i) water or sucrose solution, ii) water or saccharin solution or between iii) sucrose and saccharin solution. In both males and females, we found that control and *Nalcn^{Slc6a3}* mice showed a similar preference toward sucrose and saccharin and consumed the same volume of liquid, indicating that the appetence of solutions with high hedonic value is unaltered in *Nalcn^{Slc6a3}* mice. These new data are now presented and discussed in light of the results of the operant data in the revised manuscript (**Figure 7a-f**).

Some small errors that might distract from the arguments in the paper - but I am no expert in proof reading!

Third last line on the second paragraph on p6...Despite these results ..., OR ..that evidence...

Last line on p16 ...NALCN is required for normal pacemaker activity...

Third last line on p25....delete 'not' the experiments suggest... pacemaking activity is dispensable for...

Third last line on p27 L.C. Set up ...not 'settled up'...

The aforementioned errors have been corrected in the revised version of the manuscript.

Referee #2:

In this manuscript titled "New insight into the sodium leak channel NALCN: at the crossroad of pacemaker activity, dopamine release and psychostimulant responsiveness", Rogliardo et al., have performed electrophysiology patch-clamp recordings, behavioral analyses, microdialysis, immunological assays, and demonstrated the impact of NALCN deletion on tonic activity of VTA DA neurons, D2R-mediated signalling, reactivity to cues-predicting threat and psychostimulants responsiveness. This study contains some interesting findings and is valuable for understanding the diffenential impact of NALCN channel in midbrain DA neurons at the behavioral level. However, several methodological and interpretational limitations must be addressed to support the conclusions.

We thank the reviewer for his/her positive comments and constructive insights about our work.

Major issues:

1. Via extracellular single-unit recordings, DA cells fired with a continuum of firing patterns between two extreme cases corresponding to tonic or burst firing pattern. In the Fig.1b, all DA neurons in Nalcnslc6a3 mice-except one-are silent, lacking both tonic and burst firing activity. This makes the conclusion drawn from Fig.1 misleading. The data basically demonstrate a near-complete loss of both tonic activity and burs firing, not only tonic firing.

We thank the referee for his/her remark. We have now corrected this throughout the manuscript. To complement this analysis we have now included *in vivo* recordings showing excitatory responses in seven previously silent putative DA-neurons across *Nalcn*^{Slc6a3} male mice following repeated tail pinches (salient aversive stimulus) (**Figure 1c**). Five neurons responded by firing action potentials during the stimulus (Type 1), while two neurons fired action potentials at the end of the stimulus (Type 2), indicating that the response of VTA DA-neurons to aversive stimuli is still present in *Nalcn*^{Slc6a3} mice (**Figure 1c**). Moreover, our fiber photometry experiments (**Figure 3**) clearly demonstrate that despite the profound alterations in DA neuron activity observed under anesthetized conditions, tonic DA tone persists in awake *Nalcn*^{Slc6a3} male and female mice as. In light of these new results, we have now discussed and clarified this issue in the revised version of the manuscript.

2. In Fig.2a, the authors report no difference in basal DA levels in the nucleus accumben of Nalcnslc6a3 and control mice. This raises a critical question: How do the silent DA neurons in Nalcnslc6a3 mice release DA at levels comparable to controls? The authors must explain this discrepancy between nearly-silent neuronal firing and unaltered basal DA release.

We thank the referee for bringing up this point. This apparent discrepancy no longer exists, as our new results clearly show that the profound alterations in dopamine neuron activity are tightly linked to the experimental conditions, specifically the anesthesia. Indeed, under anesthetized conditions, dopamine neuron activity relies predominantly on intrinsic pacemaker mechanisms, whereas in awake animals ongoing network activity and synaptic inputs may sustain dopamine transmission despite marked alterations in intrinsic excitability. Nevertheless, the inclusion of additional mice in our *in vivo* microdialysis experiments confirmed our initial observations. Although not reaching statistical significance, a trend toward reduced basal extracellular DA levels in the nucleus accumbens was observed in *Nalcn*^{Slc6a3} male mice compared to controls. This new result has been included in the revised **Figure 2a**. Furthermore, in vivo fiber photometry experiments revealed that quinpirole still induced a significant reduction in the GRABDA3m signal in NalcnSlc6a3 mice. Because activation of autoD2R reduces DA release, such an effect could only be observed if DA was being tonically released

before drug administration. These results therefore demonstrate the persistence of tonic DA tone in awake Nalcn^{Slc6a3} mice despite the profound alterations in DA-neuron activity observed under anesthetized conditions. This new result has been included in the revised **Figure 3e**.

3. It is not sufficient to make the conclusion that D2R-mediated signaling at DA terminals is impaired in Nalcnslc6a3 mice from the indirect experimental data presented in Fig.3. Simuilarlly, it is not convinced that Nalcnslc6a3 male mice display supersensitivity to the psychomotor effects of cocaine via the impairment of autoD2R signaling.

We thank the referee for his/her comments. We have performed additional experiments to strength our initial conclusion that D2R-mediated signaling at DA terminals. Notably, our additional microdialysis experiments performed in both male and female mice show that eticlopride-induced increased extracellular DA level in the Acb is blunted in both male and female *Nalcn^{Slc6a3}* mice (**Figure 3g**). Using fiber photometry to assess accumbal DA dynamics we also found that the effects of both quinpirole and eticlopride were markedly reduced in *Nalcn^{Slc6a3}* mice compared with control mice, suggesting an impairment of D2 receptor-mediated regulation of DA transmission in neurons lacking NALCN (**Figure 3e-f**). We agree that our data do not strictly demonstrate that the supersensitivity to the psychomotor effects of cocaine is a direct consequence of the impairment of autoD2R signaling. We have now tuned down this claim in the revised version of the manuscript.

4. A key paradox emerges: Cocaine induces hyperlocomotion in knockout (KO) males (Fig. 5a), yet no increase in DA release is observed (Fig. 5b). The authors attribute this to "altered D2R signaling", but responses to methylphenidate/GBR12783 (Figs. 5e-h) contradict this mechanism. For instance, if D2R dysfunction were the cause, methylphenidate (which also acts on DA transporters) should elicit a similar hyperlocomotive phenotype-but the data do not support this. The authors should explain this inconsistency.
We thank the reviewer for highlighting this point. Acute hyperlocomotion induced by cocaine is commonly associated with an increase DA release in the nucleus accumbens but our results indicate a dissociation between the two events. This phenotype closely resembles that reported in mouse models lacking G protein-coupled receptor kinase 6 (PMID: 12718862), Homer1 and Homer2 (PMID: 15294147), or the norepinephrine transporter (PMID: 10769386). Importantly, evidence in the literature indicate that hyperlocomotion and DA release can be uncorrelated as illustrated in mice with decreased expression of the NR1 subunit of the NMDAR (PMID: 18185498) or lacking metabotropic glutamate receptor 5 (PMID: 11528416) which display a reduced cocaine-induced hyperlocomotion without alterations in accumbal DA levels. These examples have been now discussed in the revised version of the manuscript. Regarding his/her remark related to the responses to methylphenidate/GBR12783, we have performed additional experiments using higher doses of methylphenidate (10 mg/kg) and GBR12783 (30 mg/kg) in male and female from both genotypes. Our results revealed that while a slight increase in the first 30 min at a dose of 15 mg/kg was observed in male but not in female *Nalcn^{Slc6a3}* mice, at 30 mg/kg only female *Nalcn^{Slc6a3}* mice maintained higher locomotor responses between 30 and 90 min (**Figure 8e-f**). For methylphenidate, the psychomotor effects induced at a dose of 10 mg/kg tend to be more pronounced in both male and female *Nalcn^{Slc6a3}* mice as compared to controls (**Figure 8g-h**). These new results have been now included and discussed in the revised version of the manuscript.

5. The title positions NALCN at the "crossroad" of pacemaking, DA release, and psychostimulant responses, but pacemaker activity is only superficially characterized. Furthermore, the observed

alterations in psychostimulant responses are attributed to D2R dysfunction rather than pacemaker deficits-undermining the "crossroad" framing. The title or data should be revised to align with this mechanistic focus.

We thank the reviewer for his/her remark. We have now revised the title and the manuscript accordingly.

Other issues:

1. The manuscript lacks validation data (e.g., Western blot, immunofluorescence) confirming NALCN knockout efficiency in DA neurons. Phenotypic effects cannot be definitively attributed to NALCN loss without this test.

The *Nalcn*^{Slc6a3} mice mouse line has been extensively used independently by multiple research group, including Khaliq's laboratory (PMID: 30556810; PMID: 37640554) and Park's laboratory (PMID: 34409942). The findings from these studies - along with complementary approaches involving pharmacological interference (PMID: 36398712) or cell type–specific deletion (PMID: 39642080) - consistently converge with our results, demonstrating that NALCN channels play a key role in regulating excitability and pacemaker activity in midbrain dopaminergic neurons. We are therefore highly confident that the phenotypic alterations reported in our study are attributed to the loss of NALC in midbrain DA-neurons.

2. Cocaine hyper-locomotion occurs only in KO males (Fig. 5a vs. 5c), but hormonal influences are unexplored. Discuss potential estrogen modulation of DA excitability (e.g., via D2R expression).

We have now highlighted this point in the discussion.

3. Microdialysis measures tonic DA (minute-scale resolution), missing phasic signals critical for psychostimulant responses.

We agree with the reviewer that microdialysis assess tonic but not phasic DA. Our choice of using this approach was dictated by our initial motivation to measure basal levels of DA in the nucleus accumbens, an information we could not obtain by using other techniques as fiberphotometry.

4. Several figures are pixelated or poorly annotated.

We apologize for this. We assume the reviewer is referring to the figures provided in the supplemental material files. We have now included all the results in the main text and high resolution (300 dpi) figures are now provided.

5. Typos exist (e.g., "VTA= DA-neurons"). Correct grammatical errors and clarify phrasing.

The revised version of the manuscript has been proofread.

Dear Dr Rogliardo,

Re: JP-RP-2026-289809R1 "**Deletion of the sodium leak channel NALCN in dopamine neurons alters D2R-mediated signaling and psychostimulant responsiveness**" by Angelina Rogliardo, Tinaig Le Borgne, Aylin Gulmez, Adeline Cathala, Claire Naon, Audrey Mignon, Maelle Avrillon, Nejmeh Mashhour, Laia Castell, Claire Bernat, Jean-Michel Revest, Arnaud Monteil, Philippe Faure, Federica Bertaso, Giuseppe Gangarossa, Fabio Marti, and Emmanuel Valjent

We are pleased to tell you that your paper has been accepted for publication in The Journal of Physiology.

The last Word (or similar) version of the manuscript provided will be used by the Production Editor to prepare your proof. When this is ready you will receive an email containing a link to Wiley's Online Proofing System. The proof should be thoroughly checked and corrected as promptly as possible.

Authors should note that it is too late at this point to offer corrections prior to proofreading. Major corrections at proof stage, such as changes to figures, will be referred to the Editors for approval before they can be incorporated. Only minor changes, such as to style and consistency, should be made at proof stage. Changes that need to be made after proof stage will usually require a formal correction notice.

All queries at proof stage should be sent to: TJP@wiley.com.

Following copyediting and typesetting the accepted version will be published online.

Yours sincerely,

Jing-Ning Zhu
Senior Editor
The Journal of Physiology

IMPORTANT POINTS TO NOTE FOLLOWING ACCEPTANCE OF YOUR PAPER:

• IMPORTANT NOTICE ABOUT OPEN ACCESS: To assist authors whose funding agencies mandate immediate public access to published research findings, The Journal of Physiology allows authors to pay an Open Access (OA) fee to have their papers made freely available immediately on publication.

The Corresponding Author will receive an email from Wiley with details on how to register or log in to Wiley Authors where you will be able to place an order.

• You can check if your funder or institution has a Wiley Open Access Account here:
https://authors.wiley.com/author-resources/Journal-Authors/open-access/author-compliance-tool.html

MEMBER DISCOUNT: Corresponding authors who are Members of the Physiological Society are eligible for a 20% discount on the cost of the Article Processing Charge (APC). To claim this discount, the Corresponding Author will need to log in to the Physiological Society Membership portal and retrieve the discount code (https://www.physoc.org/journals-and-media/journals/journal-discount-for-members/). This code should then be quoted in the billing section of Wiley Authors when selecting the open access option.

• TRANSPARENT PEER REVIEW POLICY: To improve the transparency of its peer review process, The Journal of Physiology publishes online as supporting information the peer review history of all articles accepted for publication. Readers will have access to decision letters, including Editors' comments and referee reports, for each version of the manuscript as well as any author responses to peer review comments. Referees can decide whether or not they wish to be named on the peer review history document.

• You can help your research get the attention it deserves! Check out Wiley's free Promotion Guide for best-practice recommendations for promoting your work at: www.wileyauthors.com/eeo/guide. You can learn more about Wiley Editing Services which offers professional video, design, and writing services to create shareable video abstracts, infographics, conference posters, lay summaries, and research news stories for your research at: www.wileyauthors.com/eeo/promotion.

• If you would like to receive our 'Research Roundup', a monthly newsletter highlighting the cutting-edge research published in The Physiological Society's family of journals (The Journal of Physiology, Experimental Physiology, Physiological Reports, The Journal of Nutritional Physiology and The Journal of Precision Medicine: Health and Disease), please click this link, fill in your name and email address and select 'Research Roundup':

https://www.physoc.org/journals-and-media/membernews

EDITOR COMMENTS

Reviewing Editor:

The authors have satisfactorily addressed the main concerns raised in the first round of review, and both referees now support publication. I am satisfied with the revised manuscript.

Senior Editor:

Thank you for substantially revising the manuscript. Now the reviewers and the reviewing editor support acceptance for publication. Congratulations on your excellent work.

REFEREE COMMENTS

Referee #1:

This new version certainly replies at length to my comments, and there is now enough behavioral data to indicate that there are more than a few further works that need to be done to understand the differences in male and female behavior after the disturbances in NALCN presence.

These results could inform a whole new series of investigations on the roles of NALCN channels in the CNS.

Referee #2:

With the addition of new experimental results and other revisons I have no further comments and recommend accepting this -----------manuscript.

==================
