# Supplementary material for: Deletion of the sodium leak channel NALCN in dopamine neurons alters D2R‐mediated signalling and psychostimulant responsiveness
Source: J Physiol. 2026 Oct 6;604(20):8542–68. doi: 10.1113/JP289809 (PMC13674192; doi:10.1113/JP289809)
Supplement: Supplementary file 2 [file TJP-604-8542-s002.docx]

**Supplemental statistic**

**Disruption of the sodium leak channel NALCN in midbrain dopamine neurons**

**alters D2R-mediated signaling and psychostimulant responsiveness**

Angelina Rogliardo^1^, Tinaig Le Borgne^2^, Aylin Gulmez^2^, Adeline Cathala^3^, Claire Naon^1^, Audrey Mignon^1^, Maëlle Avrillon^1^, Nejmeh Mashhour^4^, Laia Castell^5#^, Claire Bernat^5^, Jean-Michel Revest^3^, Arnaud Monteil^5,6^, Philippe Faure^2^, Federica Bertaso^1^, Giuseppe Gangarossa^4,7^, Fabio Marti^2*^, Emmanuel Valjent^1*^

| **Figure** | **Measure** |  | **Groups** | **Statistical Analysis** |
| --- | --- | --- | --- | --- |
|  |  |  |  |  |
| 1d | Spontaneous activity  Cumulative probability | male | *ct* (n = 14 neurons, N = 2 mice)  *Nalcn^Slc6a3^* (n = 22 neurons, N = 3 mice) | **Wilcoxon test**  W = 212, p = 0.046*  **Kolmogorov-Smirnov test**  D = 0.461, p = 0.053 |
| 1e | Resting membrane potential | male | *ct* (n = 14 neurons, N = 2 mice)  *Nalcn^Slc6a3^* (n = 22 neurons, N = 3 mice) | **Kolmogorov-Smirnov test**  D = 0.47403, p = 0.028* |
| 1f | Current response curve | male | *ct* (n = 14 neurons, N = 2 mice)  *Nalcn^Slc6a3^* (n = 22 neurons, N = 3 mice) | **Two-way ANOVA RM**  Performed on all currents:  genotype  F_(1, 33)_ = 4.057, p = 0.052  current  F_(10, 330)_ = 30.631, p < 2e-16***  interaction  F_(10, 330)_ = 1.518, p = 0.131  Performed only on small currents (0, 10, 20, 30 pA);  genotype  F_(1, 33)_ = 6.691, p = 0.0143  current  F_(3, 99)_ = 26.048, p = 1.67e-12***  interaction  F_(3, 99)_ = 3.491, p = 0.019*  **Wilcoxon test**  *ct* vs. *Nalcn^Slc6a3^*  0 pA: W = 165, p = 0.068  10 pA: W = 205.5, p = 0.015*  20 pA: W = 208, p = 0.017*  30 pA: W = 202, p = 0.032* |
|  |  |  |  |  |
| 2a | Microdialysis DA | male  female | *ct* (mice, n = 14)  *Nalcn^Slc6a3^* (mice, n = 13)  *ct* (mice, n = 15)  *Nalcn^Slc6a3^* (mice, n = 12) | **Unpaired t-test**  t_25_ = 1.923, p = 0.066  **Unpaired t-test**  t_25_ = 0.1755, p = 0.862 |
| 2c | DAT | male and female | *ct* (mice, n = 6)  *Nalcn^Slc6a3^* (mice, n = 7) | **Unpaired t-test**  t_11_ = 1.032, p = 0.324 |
| 2e | TH  VMAT2 | male | *ct* (mice, n = 7)  *Nalcn^Slc6a3^* (mice, n = 5)  *ct* (mice, n = 7)  *Nalcn^Slc6a3^* (mice, n = 5) | **Unpaired t-test**  t_10_ = 0.6042, p = 0.558  t_10_ = 0.1988, p = 0.846 |
| 2f | pS31-TH  pS40-TH | male and female | *ct* (mice, n = 6)  *Nalcn^Slc6a3^* (mice, n = 7)  *ct* (mice, n = 6)  *Nalcn^Slc6a3^* (mice, n = 6) | **Unpaired t-test**  t_11_ = 0.2095, p = 0.838  t_10_ = 0.2765, p = 0.787 |
| 2g | Locomotion  (1/4 turns per 5 min)  Locomotion  (1/4 turns per 60 min)  Locomotion  (1/4 turns per 60 min) | male  female | *ct* (mice, n = 6)  *Nalcn^Slc6a3^* (mice, n = 8)  *ct* (mice, n = 8)  *Nalcn^Slc6a3^* (mice, n = 8) | **Two-way ANOVA RM**  genotype  F_(1, 12)_ = 2.108, p = 0.172  **Unpaired t-test**  t_12_ = 1.452, p = 0.172  **Unpaired t-test**  t_14_ = 1.295, p = 0.216 |
| 2f | Rearings | male  female | *ct* (mice, n = 6)  *Nalcn^Slc6a3^* (mice, n = 8)  *ct* (mice, n = 8)  *Nalcn^Slc6a3^* (mice, n = 8) | **Unpaired t-test**  t_12_ = 1.021, p = 0.3275  **Unpaired t-test**  t_14_ = 0.4702, p = 0.646 |
|  |  |  |  |  |
| 3b | Locomotion + Rearings  (per 60 min) | male | *ct* (mice, n = 8)  *Nalcn^Slc6a3^* (mice, n = 6) | **Unpaired t-test**  0.05 mg/kg  t_12_ = 2.357, p = 0.036*  0.5 mg/kg  t_12_ = 3.310, p = 0.006**  1 mg/kg  t_12_ = 2.285, p = 0.041* |
| 3c | Locomotion + Rearings  (per 60 min) | female | *ct* (mice, n = 7)  *Nalcn^Slc6a3^* (mice, n = 7) | **Unpaired t-test**  0.05 mg/kg  t_12_ = 0.8612, p = 0.406  0.5 mg/kg  t_12_ = 2.236, p = 0.045*  1 mg/kg  t_12_ = 2.512, p = 0.027* |
| 3e | Fiber photometry DA | male and female | *ct* (mice, n = 8)  *Nalcn^Slc6a3^* (mice, n = 9) | **Mann-Withney**  U = 13, p = 0.027* |
| 3f | Fiber photometry DA | male and female | *ct* (mice, n = 9)  *Nalcn^Slc6a3^* (mice, n = 8) | **Unpaired t-test**  T_15_ = 2.649, p = 0.018* |
| 3g | Microdialysis DA | male  female | *ct* (mice, n = 5)  *Nalcn^Slc6a3^* (mice, n = 5)  *ct* (mice, n = 4)  *Nalcn^Slc6a3^* (mice, n = 5) | **Two-way ANOVA RM**  time  F_(11, 88)_ = 5.187, p < 0.0001***  genotype  F_(1, 8)_ = 67.10, p < 0.0001***  interaction  F_(11, 88)_ = 5.026, p < 0.0001***  time  F_(11, 77)_ = 4.578, < 0.0001***  genotype  F_(1, 7)_ = 24.15, p = 0.0017**  interaction  F_(11, 77)_ = 7.491, < 0.0001*** |
| 3h | pS40-TH | male and female | *ct* (mice, n = 6)  *Nalcn^Slc6a3^* (mice, n = 4) | **Two-way ANOVA**  treatment  F_(1, 16)_ = 20.41, p = 0.0004***  genotype  F_(1, 16)_ = 7.140, p = 0.018*  interaction  F_(1, 16)_ = 7.190, p = 0.016* |
|  |  |  |  |  |
| 4a | Catalepsy | male | *ct* (mice, n = 7)  *Nalcn^Slc6a3^* (mice, n = 7)  *ct* (mice, n = 7)  *Nalcn^Slc6a3^* (mice, n = 6) | **Two-way ANOVA RM**  0.5 mg/kg  time  F_(4, 48)_ = 4.099, p = 0.021*  genotype  F_(1, 12)_ = 0.7126, p = 0.415  interaction  F_(4, 48)_ = 0.209, p = 0.684  1 mg/kg  time  F_(5, 55)_ = 12.17, p < 0.0001***  genotype  F_(1, 11)_ = 0.2512, p = 0.626  interaction  F_(5, 55)_ = 3.218, p = 0.013* |
| 4b | Catalepsy | female | *ct* (mice n = 9)  *Nalcn^Slc6a3^* (mice n = 7)  *ct* (mice n = 8)  *Nalcn^Slc6a3^* (mice n = 6) | **Two-way ANOVA RM**  0.5 mg/kg  time  F_(4, 55)_ = 2.826, p = 0.033*  genotype  F_(1, 14)_ = 2.210, p = 0.159  interaction  F_(4, 55)_ = 0.7210, p = 0.581  1 mg/kg  time  F_(3.060, 36.73)_ = 4.714, p = 0.007**  genotype  F_(1, 12)_ = 0.02804, p = 0.869  interaction  F_(5, 60)_ = 0.4538, p = 0.809 |
| 4d | Fos positive cells | male and female | *ct* (mice, n = 7)  *Nalcn^Slc6a3^* (mice, n = 7) | **Two-way ANOVA**  treatment  F_(1, 24)_ = 200.3, p < 0.0001***  genotype  F_(1, 24)_ = 0.2501, p = 0.622  interaction  F_(1, 24)_ = 0.1226, p = 0.729 |
| 4f | pS10-H3 positive cells | male and female | *ct* (mice, n = 7)  *Nalcn^Slc6a3^* (mice, n = 7) | **Two-way ANOVA**  treatment  F_(1, 24)_ = 269, p < 0.0001***  genotype  F_(1, 24)_ = 0.3159, p = 0.579  interaction  F_(1, 24)_ = 0.3159, p = 0.579 |
|  |  |  |  |  |
| 5a | Time in arms | male and female | *ct* (mice, n = 17)  *Nalcn^Slc6a3^* (mice, n = 18) | **Unpaired t-test**  open  t_33_ = 1.160, p = 0.254  closed  t_33_ = 0.02478, p = 0.980 |
| 5b | Entries in arms | male and female | *ct* (mice, n = 17)  *Nalcn^Slc6a3^* (mice, n = 18) | **Unpaired t-test**  open  t_33_ = 1.226, p = 0.229  closed  t_33_ = 0.9890, p = 0.330 |
| 5c | Licking latency | Male and female | *ct* (mice, n = 13)  *Nalcn^Slc6a3^* (mice, n = 13) | **Unpaired t-test**  t_24_ = 0.8938, p = 0.380 |
|  |  |  |  |  |
| 6b  left panel | Freezing Probability 0.7 mA  Conditioning (day 1) | male | *ct* (mice, n = 8)  *Nalcn^Slc6a3^* (mice, n = 9) | **Two-way ANOVA RM**  genotype  CS+ : F_(1, 15)_ = 0.1917, p = 0.668  CS- : F_(1, 90)_ = 0.2960, p = 0.588 |
| 6b  right panel |  | female | *ct* (mice, n = 8)  *Nalcn^Slc6a3^* (mice, n = 8) | CS+ : F_(1, 7)_ = 1.164, p = 0.317  CS- : F_(1, 7)_ = 0.7740, p = 0.408 |
| 6c  left panel | Freezing Probability 0.7 mA  Test (day 2) | male  female | *ct* (mice, n = 8)  *Nalcn^Slc6a3^* (mice, n = 9)  *ct* (mice, n = 8)  *Nalcn^Slc6a3^* (mice, n = 8) | **Paired t-test**  t_7_ = 3.419, p = 0.011*  t_8_ = 2.128, p = 0.066  **Paired t-test**  t_7_ = 4.374, p = 0.003**  **Wilcoxon test**  p = 0.078 |
| 6c  right panel | Freezing probability  (day 2 vs day 4) | male  female | *ct* (mice, n = 8)  *Nalcn^Slc6a3^* (mice, n = 9)  *ct* (mice, n = 8)  *Nalcn^Slc6a3^* (mice, n = 8) | **Paired t-test**  t_7_ = 4.374, p = 0.003**  t_8_ = 4.060, p = 0.004**  t_7_ = 2.650, p = 0.033*  t_7_ = 2.412, p = 0.047* |
| 6e | Avoidance probability  Defensive probability | male | *ct* (mice n = 6)  *Nalcn^Slc6a3^* (mice n = 7) | **Two-way ANOVA RM**  genotype  F_(1, 11)_ = 0.4234, p = 0.529  F_(1, 11)_ = 2.323, p = 0.152 |
| 6f | Avoidance probability  Defensive probability | female | *ct* (mice n = 6)  *Nalcn^Slc6a3^* (mice n = 7) | **Two-way ANOVA RM**  genotype  F_(1, 11)_ = 0.2292, p = 0.642  F_(1, 11)_ = 5.678, p = 0.036* |
| 6g | Discrimination performance | male | *ct* (mice n = 6)  *Nalcn^Slc6a3^* (mice n = 7) | **Two-way ANOVA RM**  genotype  F_(1, 11)_ = 0.09917, p = 0.759 |
| 6h | Discrimination performance | female | *ct* (mice n = 6)  *Nalcn^Slc6a3^* (mice n = 7) | **Two-way ANOVA RM**  genotype  F_(1, 11)_ = 0.9456, p = 0.352 |
|  |  |  |  |  |
| 7a | Sucrose vs water | male | ct (mice n = 8)  *Nalcn^Slc6a3^* (mice n = 6) | **Two-way ANOVA RM**  time  F_(8, 96)_ = 12.86, p < 0.0001***  genotype  F_(1, 12)_ = 0.5852, p = 0.459  interaction  F_(8, 96)_ = 1.798, p = 0.087 |
| 7b | Saccharin vs water | male | ct (mice n = 8)  *Nalcn^Slc6a3^* (mice n = 6) | **Two-way ANOVA**  time  F_(8, 96)_ = 4.732, p = 0.0031**  genotype  F_(1, 12)_ = 3.542, p = 0.084  interaction  F_(8, 96)_ = 1.222, p = 0.294 |
| 7c | Sucrose vs saccharin | male | ct (mice n = 8)  *Nalcn^Slc6a3^* (mice n = 6) | **Two-way ANOVA**  time  F_(8, 96)_ = 6.088, p = 0.0008***  genotype  F_(1, 12)_ = 1.512, p = 0.242  interaction  F_(8, 96)_ = 0.7951, p = 0.608 |
| 7d | Sucrose vs water | female | ct (mice n = 5)  *Nalcn^Slc6a3^* (mice n = 5) | **Two-way ANOVA RM**  time  F_(8, 64)_ = 8.417, p = 0.0006***  genotype  F_(1, 8)_ = 0.3697, p = 0.560  interaction  F_(8, 64)_ = 0.4559, p = 0.709 |
| 7e | Saccharin vs water | female | ct (mice n = 5)  *Nalcn^Slc6a3^* (mice n = 5) | **Two-way ANOVA**  time  F_(8, 64)_ = 7.407, p = 0.001**  genotype  F_(1, 8)_ = 0.1233, p = 0.735  interaction  F_(8, 64)_ = 1.184, p = 0.337 |
| 7f | Sucrose vs saccharin | female | ct (mice n = 5)  *Nalcn^Slc6a3^* (mice n = 5) | **Two-way ANOVA**  time  F_(8, 64)_ = 9.212, p = 0.001**  genotype  F_(1, 8)_ = 0.1340, p = 0.724  interaction  F_(8, 64)_ = 0.4658, p = 0.656 |
|  |  |  |  |  |
| 8a | Locomotion  (1/4 turns per 5 min) | male | *ct* (mice, n = 11)  *Nalcn^Slc6a3^* (mice, n = 9) | **Two-way ANOVA**  time  F_(17, 304)_ = 32.66, p < 0.0001***  genotype  F_(1, 18)_ = 10.02, p = 0.005**  interaction  F_(17, 304)_ = 9.033, p < 0.0001*** |
| 8b | Microdialysis DA | male | *ct* (mice, n = 5)  *Nalcn^Slc6a3^* (mice, n = 4) | **Two-way ANOVA RM**  time  F_(11, 77)_ = 36.22, p < 0.0001***  genotype  F_(1, 7)_ = 0.006641, p = 0.937  interaction  F_(11, 77)_ = 1.865, p = 0.057 |
| 8c | Locomotion  (1/4 turns per 5 min) | female | *ct* (mice, n = 14)  *Nalcn^Slc6a3^* (mice, n = 10) | **Two-way ANOVA RM**  time  F_(17, 374)_ = 35.56, p < 0.0001***  genotype  F_(1, 22)_ = 0.01958, p = 0.890  interaction  F_(17, 374)_ = 1.003, p = 0.454 |
| 8d | Microdialysis DA | female | *ct* (mice, n = 6)  *Nalcn^Slc6a3^* (mice, n = 3) | **Two-way ANOVA RM**  time  F_(11, 77)_ = 40.40, p < 0.0001***  genotype  F_(1, 7)_ = 0,05451, p = 0.822  interaction  F_(11, 77)_ = 0.5715, p = 0.846 |
| 8e | Locomotion  (1/4 turns per 5 min) | male | *ct* (mice, n = 8)  *Nalcn^Slc6a3^* (mice, n = 8)  *ct* (mice, n = 8)  *Nalcn^Slc6a3^* (mice, n = 12) | **Two-way ANOVA RM**  15 mg/kg  time  F_(23, 322)_ = 19.82, p < 0.0001***  genotype  F_(1, 14)_ = 0,8593, p = 0.370  interaction  F_(23, 322)_ = 2.563, p = 0.0001***  30 mg/kg  time  F_(23, 414)_ = 9.595, p < 0.0001***  genotype  F_(1, 18)_ = 0.08318, p = 0.776  interaction  F_(23, 414)_ = 1.745, p = 0.151 |
| 8f | Locomotion  (1/4 turns per 5 min) | female | *ct* (mice, n = 8)  *Nalcn^Slc6a3^* (mice, n = 6)  *ct* (mice, n = 11)  *Nalcn^Slc6a3^* (mice, n = 10) | **Two-way ANOVA RM**  15 mg/kg  time  F_(17, 204)_ = 13.54, p < 0.0001***  genotype  F_(1, 12)_ = 1,288, p = 0.2786  interaction  F_(17, 204)_ = 0.8244, p = 0.664  30 mg/kg  time  F_(23, 437)_ = 10.24, p < 0.0001***  genotype  F_(1, 19)_ = 3.310, p = 0.085  interaction  F_(23, 437)_ = 1.716, p = 0.154 |
| 8g | Locomotion  (1/4 turns per 5 min) | male | *ct* (mice, n = 8)  *Nalcn^Slc6a3^* (mice, n = 6)  *ct* (mice, n = 8)  *Nalcn^Slc6a3^* (mice, n = 10) | **Two-way ANOVA RM**  7.5 mg/kg  time  F_(17, 204)_ = 13.54, p < 0.0001***  genotype  F_(1, 12)_ = 1.288, p = 0.279  interaction  F_(17, 204)_ = 0.8244, p = 0.664  10 mg/kg  time  F_(17, 272)_ = 37.73, p < 0.0001***  genotype  F_(1, 16)_ = 1.209, p = 0.288  interaction  F_(17, 272)_ = 1.933, p = 0.116 |
| 8h | Locomotion  (1/4 turns per 5 min) | female | *ct* (mice, n = 7)  *Nalcn^Slc6a3^* (mice, n = 7)  *ct* (mice, n = 8)  *Nalcn^Slc6a3^* (mice, n = 8) | **Two-way ANOVA RM**  7.5 mg/kg  time  F_(17, 204)_ = 32.28, p < 0.0001***  genotype  F_(1, 12)_ = 0,2359, p = 0.636  interaction  F_(17, 204)_ = 2.032, p = 0.0111*  10 mg/kg  time  F_(17, 238)_ = 26.51, p < 0.0001***  genotype  F_(1, 14)_ = 1.918, p = 0.188  interaction  F_(17, 238)_ = 1.798, p = 0.151 |
|  |  |  |  |  |
| 9b | D1R  GluA1 | male and female | *ct* (mice n = 6)  *Nalcn^Slc6a3^* (mice n = 7) | **Unpaired t-test**  t_11_ = 1.246, p = 0.239  t_11_ = 0.8465, p = 0.415 |
| 9d | DARPP-32  Gαolf | male and female | *ct* (mice n = 6)  *Nalcn^Slc6a3^* (mice n = 7) | **Unpaired t-test**  t_11_ = 0.5534, p = 0.591  t_11_ = 1.22, p = 0.2479 |
| 9e | Locomotion  (1/4 turns per 5 min) | male  female | *ct* (mice n = 7)  *Nalcn^Slc6a3^* (mice n = 8)  *ct* (mice n = 8)  *Nalcn^Slc6a3^* (mice n = 7) | **Two-way ANOVA RM**  time  F_(17, 221)_ = 5.944, p = 0.0003***  genotype  F_(1, 13)_ = 0.1335, p = 0.721  interaction  F_(13, 221)_ = 7.932, p < 0.0001***  time  F_(17, 221)_ = 5.834, p < 0.0001***  genotype  F_(1, 13)_ = 0.01665, p = 0.899  interaction  F_(17, 221)_ = 1.133, p = 0.352 |
|  |  |  |  |  |
| 10a | Locomotion day 1  (1/4 turns per 5 min)  Rearing | male | *ct* (mice, n = 11)  *Nalcn^Slc6a3^* (mice, n = 11) | **Two-way ANOVA RM**  time  F_(23, 460)_ = 6.284, p = 0.0001***  genotype  F_(1, 20)_ = 1.560, p = 0.226  interaction  F_(23, 460)_ = 0.9528, p = 0.527  time  F_(23, 460)_ = 14.41, p < 0.0001***  genotype  F_(1, 20)_ = 0.4361, p = 0.517  interaction  F_(23, 460)_ = 0.8896, p = 0.613 |
| 10b | Microdialysis DA | male | *ct* (mice, n = 4)  *Nalcn^Slc6a3^* (mice, n = 4) | **Two-way ANOVA RM**  Time  F_(11, 66)_ = 28.70, p < 0.0001***  Genotype  F_(1, 6)_ = 1.620, p = 0.250  Interaction  F_(11, 66)_ = 2.005, p = 0.042* |
| 10c | Locomotion day 15  (1/4 turns per 5 min)  Rearing | male | *ct* (mice, n = 11)  *Nalcn^Slc6a3^* (mice, n = 10) | **Two-way ANOVA RM**  time  F_(23, 431)_ = 33.54, p < 0.0001***  genotype  F_(1, 19)_ = 3.043, p = 0.097  interaction  F_(23, 431)_ = 3.041, p < 0.0001***  time  F_(23, 431)_ = 10.80, p < 0.0001***  genotype  F_(1, 19)_ = 4.460 , p = 0.048*  interaction  F_(23, 431)_ = 4.524, p < 0.0001*** |
| 10d | Locomotion + rearing  (day 1 vs day 15)  (1/4 turns per 5 min) | male | *ct* (mice n = 11)  *Nalcn^Slc6a3^* (mice n = 10) | **Two-way ANOVA RM**  day  F_(1, 19)_ = 63.35, p < 0.0001***  genotype  F_(1, 19)_ = 2.807, p = 0.110  interaction  F_(1, 19)_ = 2.518, p = 0.129 |
| 10e | Locomotion day 1  (1/4 turns per 5 min)  Rearing | female | *ct* (mice, n = 8)  *Nalcn^Slc6a3^* (mice, n = 9) | **Two-way ANOVA RM**  time  F_(23, 345)_ = 4.766, p = 0.0057**  genotype  F_(1, 15)_ = 1.179, p = 0.295  interaction  F_(23, 345)_ = 1.675, p = 0.028*  time  F_(23, 345)_ = 14.56, p < 0.0001***  genotype  F_(1, 15)_ = 0.2405, p = 0.631  interaction  F_(23, 345)_ = 1.367, p = 0.123 |
| 10f | Microdialysis DA | female | *ct* (mice, n = 4)  *Nalcn^Slc6a3^* (mice, n = 4) | **Two-way ANOVA RM**  time  F_(7, 77)_ = 36.24, p<0.0001***  genotype  F_(1, 7)_ = 8.115, p = 0.025*  interaction  F_(7, 77)_ = 5.716, p<0.0001*** |
| 10g | Locomotion day 15  (1/4 turns per 5 min)  Rearing | female | *ct* (mice, n = 8)  *Nalcn^Slc6a3^* (mice, n = 9) | **Two-way ANOVA RM**  time  F_(23, 321)_ = 30.85, p < 0.0001***  genotype  F_(1,15 )_ = 7.303, p = 0.016*  interaction  F_(23, 321)_ = 3.695, p < 0.0001***  time  F_(23, 321)_ = 7.944, p < 0.0001***  genotype  F_(1, 15)_ = 8.965, p = 0.009**  interaction  F_(23, 321)_ = 8.868, p < 0.0001*** |
| 10h | Locomotion + rearing  (day 1 vs day 15)  (1/4 turns per 5 min) |  | *ct* (mice, n = 8)  *Nalcn^Slc6a3^* (mice, n = 9) | **Two-way ANOVA RM**  day  F_(1, 15)_ = 66.93, p < 0.0001***  genotype  F_(1, 15)_ = 9.275, p = 0.008**  interaction  F_(1, 15)_ = 4.560, p = 0.0496* |
| S1a | Fiber photometry DA (L-DOPA) | male and female | *ct* (mice, n = 3)  *Nalcn^Slc6a3^* (mice, n = 2) | **Paired t-test: 470 vs 405 nm**  T_4_ = 6.347, p = 0.003157** |
| S1b | Fiber photometry DA (quinpirole) | male and female | *ct* (mice, n = 8)  *Nalcn^Slc6a3^* (mice, n = 9) | **Paired t-test: quinpirole vs saline**  ct: t_7_ = -5.244, p = 0.001194**  **wilcoxon signed-rank test: quinpirole vs saline**  *Nalcn^Slc6a3^*: W = 0.000, p = 0.003906** |
| S1c | Fiber photometry DA (eticlopride) | male and female | *ct* (mice, n = 9)  *Nalcn^Slc6a3^* (mice, n = 8) | **Paired t-test: eticlopride vs saline**  ct: t_8_ = 3.770, p = 0.005466**  *Nalcn^Slc6a3^*: t_7_ = 3.233, p = 0.0144* |
|  |  |  |  |  |
